# Prediction of Spontaneous Protein Deamidation from Sequence-Derived Secondary Structure and Intrinsic Disorder

**DOI:** 10.1371/journal.pone.0145186

**Published:** 2015-12-16

**Authors:** J. Ramiro Lorenzo, Leonardo G. Alonso, Ignacio E. Sánchez

**Affiliations:** 1 Instituto de Investigaciones en Ingeniería Genética y Biología Molecular, Consejo Nacional de Investigaciones Científicas y Técnicas, Buenos Aires, Argentina; 2 Protein Structure-Function and Engineering Laboratory, Fundación Instituto Leloir and IIBBA—CONICET, Buenos Aires, Argentina; 3 Protein Physiology Laboratory, Departamento de Química Biológica, Facultad de Ciencias Exactas y Naturales and IQUIBICEN—CONICET, Universidad de Buenos Aires, Buenos Aires, Argentina; Swiss Institute of Bioinformatics, SWITZERLAND

## Abstract

Asparagine residues in proteins undergo spontaneous deamidation, a post-translational modification that may act as a molecular clock for the regulation of protein function and turnover. Asparagine deamidation is modulated by protein local sequence, secondary structure and hydrogen bonding. We present NGOME, an algorithm able to predict non-enzymatic deamidation of internal asparagine residues in proteins in the absence of structural data, using sequence-based predictions of secondary structure and intrinsic disorder. Compared to previous algorithms, NGOME does not require three-dimensional structures yet yields better predictions than available sequence-only methods. Four case studies of specific proteins show how NGOME may help the user identify deamidation-prone asparagine residues, often related to protein gain of function, protein degradation or protein misfolding in pathological processes. A fifth case study applies NGOME at a proteomic scale and unveils a correlation between asparagine deamidation and protein degradation in yeast. NGOME is freely available as a webserver at the National EMBnet node Argentina, URL: http://www.embnet.qb.fcen.uba.ar/ in the subpage “Protein and nucleic acid structure and sequence analysis”.

## Introduction

Protein deamidation is a post-translational modification in which the side chain amide group of a glutamine or asparagine residue is transformed into an acidic carboxylate group [1]. Non-enzymatic deamidation of asparagine is faster than of glutamine and hence presents higher physiological significance [2], being involved in processes such as apoptosis, brain development and aging [3]. Deamidation often [3], but not always [4], leads to loss of protein function. Deamidation rates in proteins vary widely, with halftimes for particular Asn residues ranging from several days to years. Deamidation may thus act as a molecular clock for protein function and turnover [5] and may take place during recombinant protein purification and during storage of therapeutic proteins.

**Fig 1:**
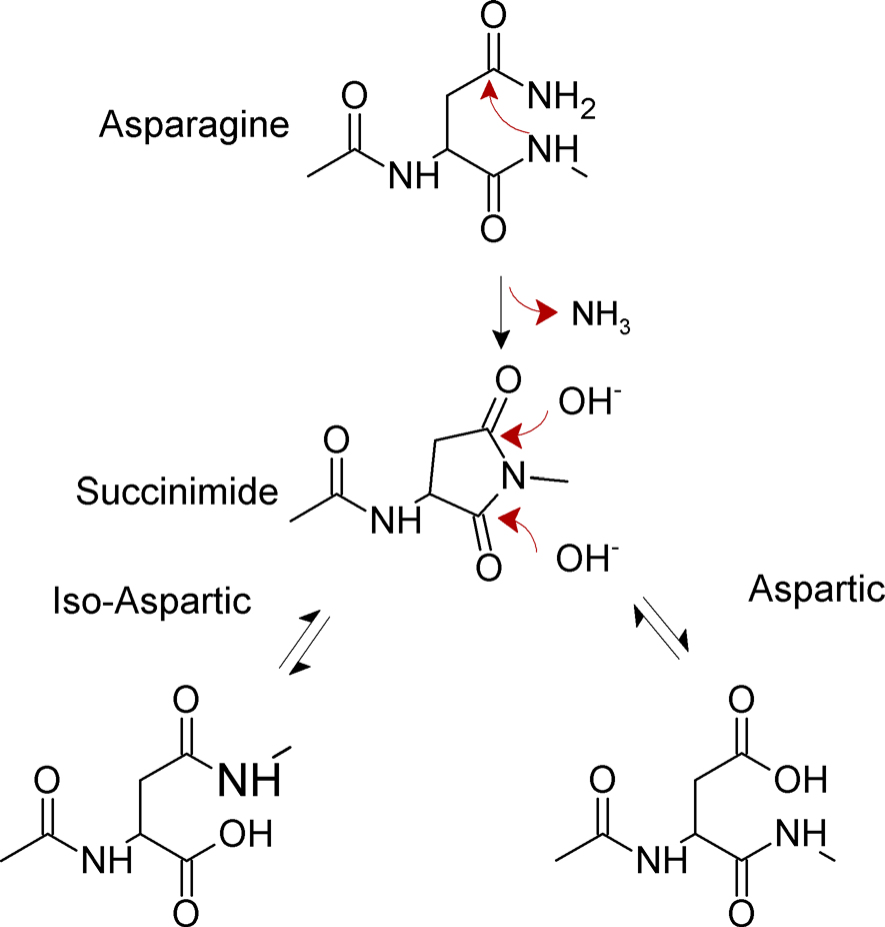
Mechanism for spontaneous deamidation of internal asparagine residues in proteins.

Non-enzymatic deamidation at internal asparagine residues in proteins occurs near neutral pH through an intramolecular rearrangement and involves two steps [1, 3]. In the first, rate-limiting step, the backbone amide nitrogen atom of the first amino acid residue right next to the C-terminal end of the Asn (referred to from now on as the N + 1 amino acid) attacks the carbonyl carbon of the asparagine or glutamine side chain forming a cyclic imide (Fig 1).

In unstructured peptides, the kinetics of succinimide formation is mainly affected by the identity of the N+1 residue, showing an inverse relationship with the bulkiness of the amino acid side chain [6]. When glycine is present in the N+1 position of an asparagine residue, the deamidation half-life is in the order of days while the presence of the phenylalanine slows the deamidation half-life to years. In proteins, structure strongly affects the kinetics of succinimide formation by imposing conformational constraints to both the amide nitrogen and the asparagine side chain [7].

In the second reaction step (Fig 1), the cyclic imide is hydrolyzed at either the alpha or beta carbonyl group, yielding iso-aspartate (isoAsp) and aspartate, in a ratio of approximately 3:1 in model peptides. Most organisms indeed possess the enzyme L-isoAsp-O-methyltransferase (PIMT) that specifically restores aspartic acid residues from iso-aspartic. Loss of this enzyme has harmful consequences [3]. Since the cyclic intermediate can undergo racemization, the final reaction products include both L- and D- Asp and isoAsp. In any case, upon asparagine deamidation a new acidic group appears in the protein. Asparagine glycosylation is a common post-translational modification that can occur in eukaryotes, bacteria and archaea at N[^P][ST] sequences. Glycosylated asparagines are not prone to deamidation due to the attached glycan.

In contrast with the ubiquity and importance of asparagine deamidation, there is currently no publicly available algorithm for the prediction of Asn deamidation in proteins in general. A structure-based algorithm was published [7], showing a good predictive power but limited to those proteins with known three dimensional structure, a serious limitation considering that structure has been resolved only for a small subset of proteins. To our knowledge this algorithm is no longer available online. A second structure-based algorithm was recently reported, but its scope is limited to antibody variable regions [8]. We present NGOME, a sequence-based method for the prediction of asparagine deamidation from predicted structural features. We envision that NGOME will be useful to systematically evaluate whole proteome data and in the study of intrinsically disordered proteins for which the structural data is scarce. The analysis of specific case studies shows that NGOME can give insights into the spontaneous deamidation of individual proteins, as well as link deamidation with protein turnover in whole proteomes.

## Algorithm

### Computational definition of features modulating deamidation

In the absence of secondary and tertiary structure, asparagine deamidation rates are governed by the identity of the N+1 amino acid [7]. In model peptides, the Asn-Gly dipeptide is by far the fastest to deamidate, with bulky N+1 side chains generally slowing down the reaction. On the other hand, many conformational constraints decreasing Asn deamidation rates have also been identified, including alpha helix formation [9] and hydrogen bond formation by the Asn side chain, the N+1 backbone amide and the neighboring residues [7]. These conformational constraints slow down deamidation by restricting the nucleophilic attack of the backbone amide to the side chain carbonyl carbon of the asparagine. During the development of NGOME, we evaluated a larger set of conformational constraints, including the propensity to adopt beta sheet conformation. However, only alpha helix formation and intrinsic disorder were found useful to predict deamidation. In NGOME, conformational constraints are weighted in two main factors, the tendency to adopt alpha helix and intrinsic disorder. The input of NGOME is a protein sequence. NGOME calculates t_50_ for each internal Asn in the sequence as follows:

$$t_{50}=t_{50}(sequence)\cdot exp\left(w_{H}\cdot H+w_{O}\cdot \left(1-D\right)\right)$$ (1)

t_50_(sequence) is the deamidation half-time of the N,N+1 dipeptide in model peptides [7]. Similar to the quantitative analysis of amide hydrogen exchange in proteins, the observed deamidation half time is the product of the intrinsic deamidation half time as observed in unstructured peptides, times a protection factor that describes the slowing down of deamidation by conformational constraints [7]. H is 1 if the Asn residue is in a helix as predicted by JPred [10] and 0 otherwise. D is the disorder score for the Asn residue from the IUPRED algorithm and ranges from 0 (fully ordered) to 1 (fully disordered) [11]. IUPRED scores correlate with backbone dynamics as measured by NMR [12] and are used here as a proxy for local hydrogen bond formation. w_H_ and w_O_ are empirical weights (see below for their estimation).

### Dataset

We compiled a database of 281 asparagine residues (67 positives and 214 negatives) in 39 proteins to train NGOME (see S1 Table in S1 File). We collected from the literature experimental reports of deamidation of Asn residues in proteins using mass spectrometry or Edman sequencing. Since deamidation rates depend strongly on pH and temperature, we only included experiments at neutral or slightly basic pH and up to 313 degrees Kelvin. An Asn residue was considered positive for deamidation if unequivocal change to aspartic or isoaspartic residue was observed. If quantitative data were available, we labeled the Asn residue as positive if conversion was at least 50%, with a half-time <100 days. An Asn residue was considered a negative for deamidation if deamidation was tested for and absent, or had a halftime >100 days. Asparagine residues for which direct experimental evidence was not obtained were not taken into account. Proteins obtained from natural sources were included in the dataset only if sample age was reported.

### Algorithm training

We trained NGOME by randomly splitting the dataset into training and test sets 100 times. The splitting was performed so that both training and test sets had a proportion of experimental positives and negatives as close as possible to the proportion in the full dataset. For each splitting, we selected w_H_ and w_O_ to maximize the area under the receiver operating characteristic (ROC) curve for the training set. For the test set, the area under the ROC curve for NGOME was larger than for sequence-based prediction 97 out of 100 times (average difference 0.0334±0.0096). Finally, we selected the average values of w_H_ (0.571) and w_O_ (2.989) for NGOME.

**Fig 2:**
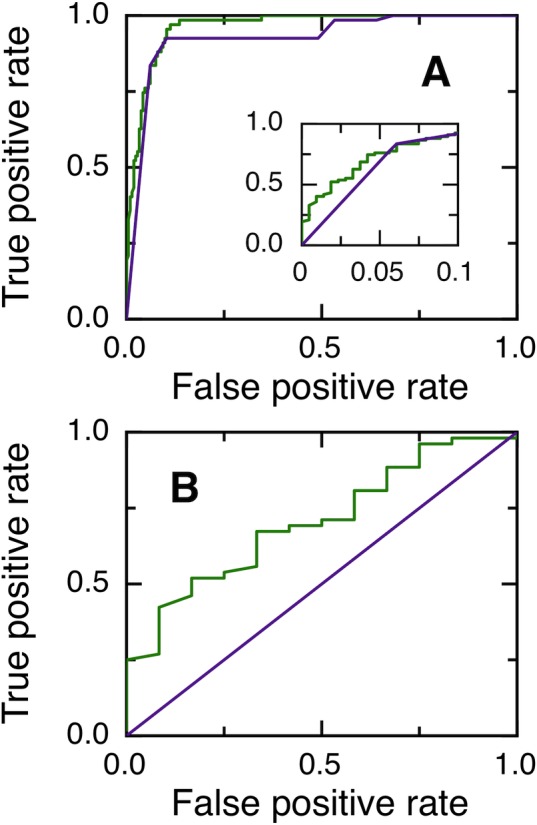
Prediction of asparagine deamidation using NGOME and sequence-only methods. (A) ROC curves for 281 asparagine residues (67 positives and 214 negatives) from 39 proteins. The inset highlights the low false positive rate region of the curve. NGOME: Green line. Sequence only: purple line. (B) ROC curves for 64 Asn-Gly dipeptides residues (52 positives and 12 negatives) from 35 proteins. NGOME: Green line. Sequence only: Purple line.

The performance of NGOME is shown in Fig 2, in comparison with predictions using t_50_(sequence) only. We computed t_50_ for all Asn in the dataset and generated a ROC curve by considering as positives Asn residues with different values of t_50_ (Fig 2A). The area under the ROC curve is larger for the NGOME predictions (green line, 0.9640) than for the sequence-based predictions (purple line, 0.9270) (p-value 6⋅10^−3^ [13]). NGOME also performs better for threshold values yielding few false positives (Fig 2A, inset).

We next considered Asn-Gly dipeptides, which deamidate the fastest in the absence of structure and are thus more likely to be of biological significance. However, only 52 of the 64 Asn-Gly dipeptides in our dataset are positive, confirming that structure plays an important role in determining Asn-Gly deamidation rates. t_50_(sequence) can not discriminate between positives and negatives in Asn-Gly dipeptides because it only takes the identity of the N+1 residue into account. On the other hand, Fig 2B shows that NGOME can discriminate between positive and negative Asn-Gly dipeptides. The area under the ROC curve is 0.7051 for the NGOME predictions (green line), larger than the random value of 0.5 for sequence-based prediction (purple line) (p-value 9⋅10^−3^ [13]).

To sum up, NGOME identifies fast deamidating Asn residues better than sequence-based predictions for both the full database and fast deamidating Asn-Gly dipeptides.

## Online Implementation

We implemented NGOME online at www.embnet.qb.fcen.uba.ar, in the subpage “Protein and nucleic acid structure and sequence analysis”. The user of NGOME provides as query a protein sequence in fasta format. A warning appears if the query sequence has few related sequences and the JPred secondary structure prediction was performed using only the query sequence.

The output of the NGOME server is both numerical and graphical. The first two tables in the output list the following attributes for all Asn residues in the query sequence: (1) Identity of the N+1 residue (2) Predicted status as positive or negative, using a threshold giving a false positive rate of 5% (3) Predicted value of t_50_(NGOME) (4) Predicted value of t_50_(sequence) (5) The corresponding protection factor P = t_50_(NGOME)/t_50_(sequence) (6) Predicted percent deamidation after a user-given time (default: 2 days), assuming an exponential decay (7) Whether the asparagine belongs to a N[^P][ST] sequence and thus may be glycosylated. The first table is sorted by residue number, while the second table is sorted by t_50_(NGOME). The purpose of these tables is to pinpoint deamidation-prone asparagines in the protein sequence of interest.

A second pair of tables include the same data for all positions of the query sequence, in the hypothetical case that they were occupied by an asparagine. The third table is sorted by residue number, while the fourth table is sorted by t_50_(NGOME). This experiment tests whether an asparagine residue introduced by a point mutation at a particular position would deamidate according to NGOME. The secondary structure and disorder predictions are taken from the wild type sequence. The JPred secondary structure predictions are based on an alignment of sequences homologous to the query sequence [10] and are thus unlikely to be strongly affected by a point mutation in the query. The IUPRED predictions are based on predicted residue interactions for a 21-residue window and are also unlikely to be strongly affected by a point mutation in the query. Nevertheless, if the user identifies a sequence position of potential interest, we suggest running NGOME for the mutant sequence.

Two figures show the logarithm of t_50_(NGOME), t_50_(sequence) and the protection factor as a function of residue number (see the case studies section below for representative examples). Sequence positions with an Asn in the query sequence are highlighted. This visualization tool locates regions of the protein where the structure can protect an asparagine from deamidation. Finally, a third figure shows the predicted percent deamidation after a user-given time (default: 2 days), for both individual asparagines and the protein as a whole. This calculation assumes that the deamidation reactions of individual Asn residues are independent. t_50_(Protein) can be useful in tuning the lifetime of a particular protein, rather than focusing on individual asparagines.

The server also includes extensive documentation about protein deamidation, a guide to interpret the results and the analysis of four case studies of medical and biotechnological interest: superoxide dismutase, BCL-xL protein, human interferon beta and Trastuzumab heavy chain.

## Case Studies

### Superoxide dismutase

Recently, a relationship has been established between several missense N to D mutations in the Cu, Zn Superoxide Dismutase (SOD) protein and the onset of amyotrophic lateral sclerosis (ALS) [14]. Interestingly, the N to D replacement can also be obtained by the spontaneous deamidation of an asparagine residue. The SOD protein is expected to endure a lifetime on the order of 1.4 years when traversing an axon that is 1 m in length at a rate of 2 mm/day. This protein lifetime allows slow deamidation events to take place and suggests that deamidation could explain in some cases the sporadic onset of ALS. Mutation of N26, N131 and N139 to D in the recombinant enzyme decreases SOD conformational stability and accelerates SOD fibrillation [14]. This suggests that deamidation of N26, N131 and/or N139 may lead to loss of SOD function and increase the concentration of cytotoxic oligomeric species. N26 is the most deamidation-prone residue in SOD, and 23% of the protein purified from human red blood cell shows aspartate instead of asparagine in the position 26 [14].

**Fig 3:**
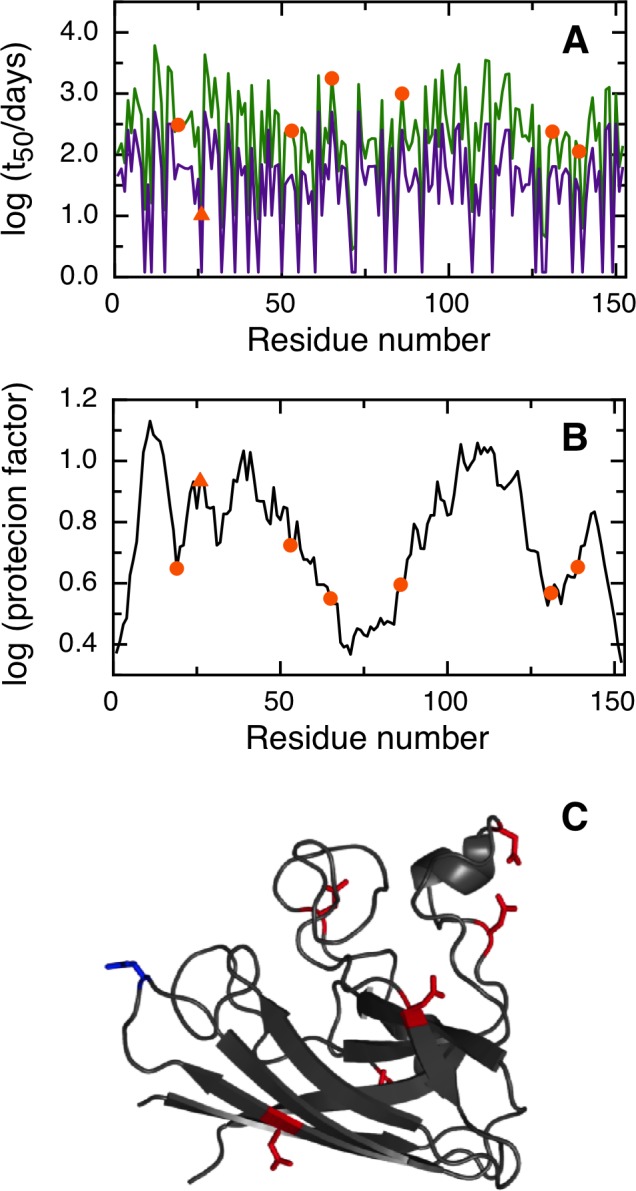
Prediction of asparagine deamidation for the human superoxide dismutase protein using NGOME and sequence-only methods. The symbols highlight asparagine residues, while the lines are the predictions for all positions of the query sequence, in the hypothetical case that they were occupied by an asparagine. The triangles highlight the fastest deamidating asparagine residues according to experiment. (A) t_50_(NGOME) (green line) and t_50_(sequence) (purple line) as a function of residue number. (B) Protection factor as a function of residue number. (C) Cartoon structure representation of the human superoxide dismutase monomer. Asparagine side chains are highlighted (deamidation sites in blue, other sites in red).

NGOME successfully predicts N26 as the fastest deamidating asparagine in SOD, with an estimated t_50_ of 10.3 days (Fig 3A). We would like to remark that N26 shows a relatively large protection factor, indicating that local order in the SOD structure (Fig 3C) leads to nine-folds lower deamidation of this key residue compared to an unstructured peptide (Fig 3B). NGOME also predicts that the second and third-fastest deamidating asparagine residues in SOD are N131 and N139, with estimated t_50_-values of 237 and 113 days respectively. It should be noted that N131 and N139 are predicted to deamidate slowly, yet at a rate that is consistent with the SOD lifecycle and with late onset ALS. We conclude that NGOME is able to pinpoint deamidation-prone asparagines that could be responsible for SOD destabilization and misfolding events often observed in late onset ALS.

### BCL-xL

Deamidation is generally regarded as a degradation process by which a protein loses its function. However, in some cases deamidation regulates key events in living cells. A paradigmatic case of the role of deamidation in the regulation of protein activity is BCL-xL, a component of the apoptotic response to DNA-damaging antineoplastic agents [15]. In basal conditions, the BCL-2 family members (BCL 2 and BCL-xL) block the pro-apoptotic activity of BH3 domain-only proteins. Cis-platin antineoplastic drugs cause DNA damage, which induces deamidation of BCL-xL at conserved asparagine residues 52 and 66 in an unstructured loop of the protein [15]. This overrides the anti-apoptotic activity of BCL-xL, the Bak and Bax proteins are activated and the caspase cascade is initiated with the consequent cellular apoptosis.

**Fig 4:**
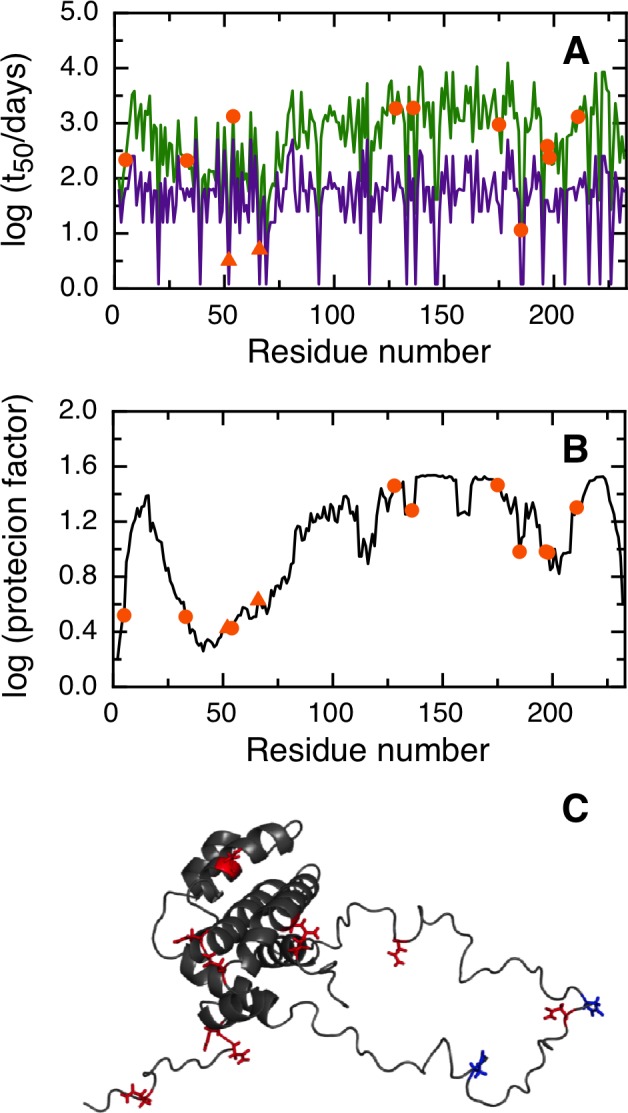
Prediction of asparagine deamidation for the human BCL-xL protein using NGOME and sequence-only methods. The symbols highlight asparagine residues, while the lines are the predictions for all positions of the query sequence, in the hypothetical case that they were occupied by an asparagine. The triangles highlight the fastest deamidating asparagine residues according to experiment. (A) t_50_(NGOME) (green line) and t_50_(sequence) (purple line) as a function of residue number. (B) Protection factor as a function of residue number. (C) Cartoon structure representation of the human BCL-xL protein. Asparagine side chains are highlighted (deamidation sites in blue, other sites in red).

NGOME correctly predicts that the two fastest deamidating asparagines in human BCL-xL are residues 52 and 66 (Fig 4A). In this case, NGOME accurately identified the molecular effectors (N52 and N66) that trigger apoptosis. N52 has an estimated t_50_ of 3.2 days and N66 an estimated t_50_ of 5.1 days. NGOME helps visualize the interplay between structure and sequence in protein deamidation. A sequence-based prediction would suggest three deamidation-prone asparagines in BCL-xL (Fig 4A, purple line). However, structure in the C-terminal half of the molecule (Fig 4B and 4C) slows down N183 from deamidation relative to N52 and N66 (Fig 4A, green line).

### Interferon beta

Recombinant Interferon beta is widely used for the treatment of relapsing-remitting multiple sclerosis [16]. Interferon beta deamidation is a slow process that does not generally impact the activity of the wild type protein under physiological conditions. However, recombinant Interferon beta is a commercial drug that must remain nearly unchanged for a long time, usually two years, during the product lifecycle. In this scenario, slow asparagine deamidation gains critical relevance. It has been experimentally observed that asparagine 46 suffers deamidation in the lapse of days to months [16]. In this case, deamidation increases the biological activity of the drug [16].

**Fig 5:**
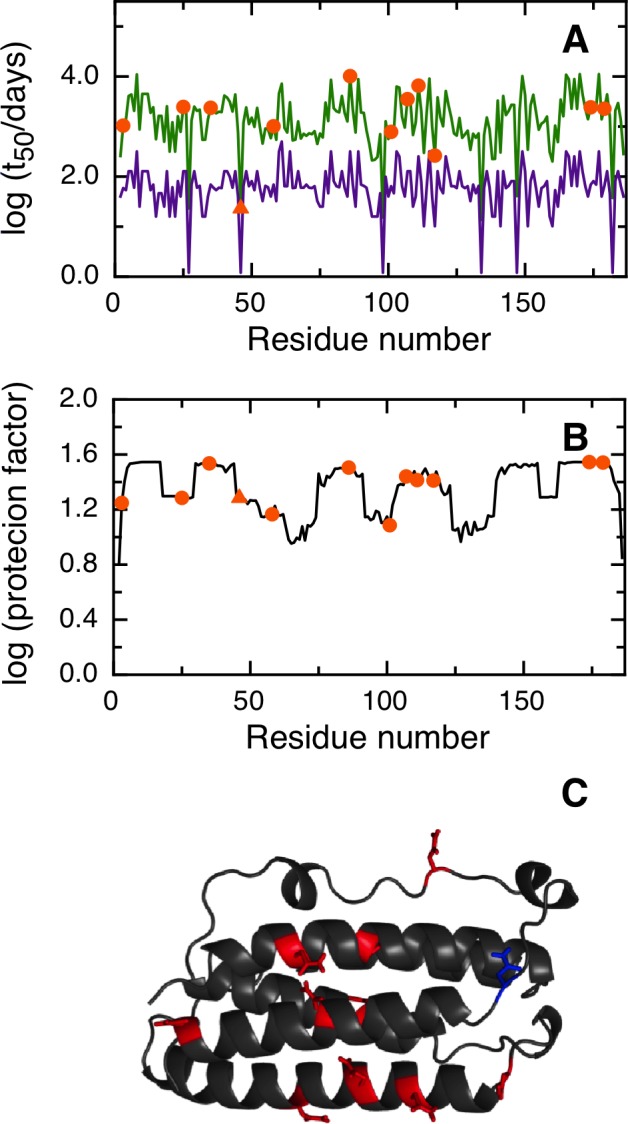
Prediction of asparagine deamidation for the interferon beta protein using NGOME and sequence-only methods. The symbols highlight asparagine residues, while the lines are the predictions for all positions of the query sequence, in the hypothetical case that they were occupied by an asparagine. The triangles highlight the fastest deamidating asparagine residues according to experiment. (A) t_50_(NGOME) (green line) and t_50_(sequence) (purple line) as a function of residue number.(B) Protection factor as a function of residue number. (C) Cartoon structure representation of the interferon beta protein. Asparagine side chains are highlighted (deamidation sites in blue, other sites in red).

NGOME predicts that all asparagines in Interferon beta deamidate slowly (Fig 5). Nevertheless, the top ranked asparagine 46 is predicted to deamidate with a t_50_ of 23.1 days, in agreement with experimental data. This is due to a strong sequence propensity (Fig 5A, purple line) that overrules an overall strong protection from deamidation by conformational constrains (Fig 5B and 5C). The identification of deamidation prone residues in biotechnological products is a useful tool in formulation development.

### Trastuzumab heavy chain

Therapeutic monoclonal antibodies are by far the fastest growing biological drugs segment. As with every biotherapeutic drug, the structural identity of the protein should be preserved within narrow ranges during the whole lifecycle of the product. However, it is not possible to exclude all possible degradation prone sequences, including asparagine deamidation, from the whole protein or even from CDR regions. Trastuzumab is a commercial therapeutic monoclonal antibody used to treat Her2 positive breast cancer. Residues N55 in the CDR2 region and N388, N393 or N394 in the CH3 of Trastuzumab heavy chain have been reported to deamidate [17, 18].

**Fig 6:**
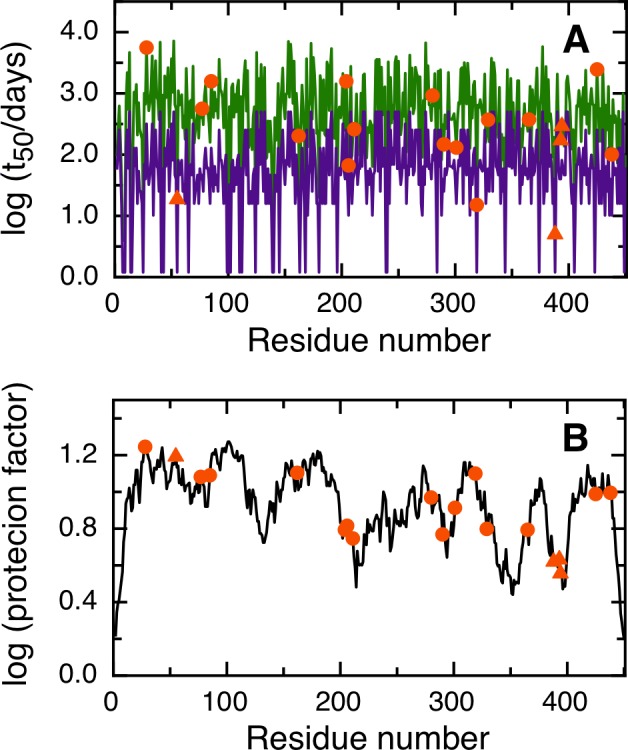
Prediction of asparagine deamidation for the Trastuzumab heavy chain using NGOME and sequence-only methods. The symbols highlight asparagine residues, while the lines are the predictions for all positions of the query sequence, in the hypothetical case that they were occupied by an asparagine. The triangles highlight the fastest deamidating asparagine residues according to experiment. (A) t_50_(NGOME) (green line) and t_50_(sequence) (purple line) as a function of residue number.(B) Protection factor as a function of residue number.

NGOME correctly predicts the deamidation of N55 in the CH3 of the heavy chain of Trastuzumab and points at N388 as the main site of deamidation in the CDR2 region (Fig 6). N319 is also predicted to be deamidation-prone, but has not been reported to deamidate. Monoclonal antibodies are heterodimeric proteins formed by two light chains, linked by intra and interchain disulfide bridges. Since quaternary structure and disulfide bonds are not considered by NGOME, we find the correlation between experiment and prediction encouraging.

### Protein turnover in yeast

Our last case study does not deal with an individual protein, but with protein dynamics at an organismic scale. The turnover rates of over 3700 yeast proteins during exponential growth have been reported [19] and short and long-lived proteins were analyzed for enrichment of various physical attributes. A significant enrichment of serine and asparagine in short-lived proteins was observed [19]. The enrichment of serine in short-lived proteins was rationalized by relating it to serine-rich sequences that target proteins for degradation, such as the PEST sequence. The enrichment of asparagine in short-lived proteins was left unexplained.

**Fig 7:**
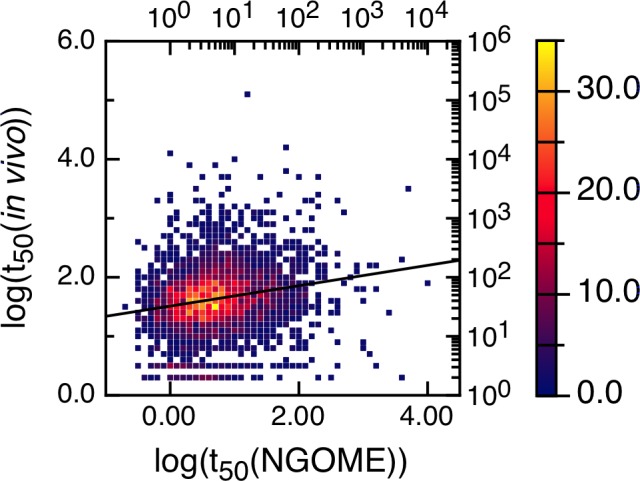
Correlation between experimental yeast protein half-lives (in minutes) and t_50_(Protein)-values (in days) predicted by NGOME. For better visualization, the 3750 data points were binned using half-live intervals of 0.1 log units and colored according to the number of points in each bin (color axis to the right). The line is the best linear fit to all points. The correlation coefficient R is 0.20 (p-value 10^−28^).

We have looked for a correlation between the logarithm of the experimental protein half-lives and the logarithm of the t_50_(Protein)-values provided by our algorithm. We performed NGOME predictions for all proteins in [19] (see S2 Table in S1 File). We used for the correlation only the in vivo t_50_-values with a positive sign [19]. Fig 7 shows that short-lived proteins have shorter t_50_(Protein)-values for deamidation. The R-value is 0.2, indicating that deamidation is not the main determinant of protein turnover in vivo. However, the p-value is 10^−28^, indicating a statistically strong correlation.

We propose two possible explanations for this correlation. The first explanation starts with the fact that deamidation leads in most cases to loss of protein function and decreases organism fitness [20]. Thus, there is a selection pressure against deamidation, which is weaker for short-lived proteins because they degrade faster than they deamidate. A second explanation is that spontaneous asparagine deamidation may act as a degradation signal for a subset of yeast proteins. In the case of yeast, degradation may be mediated by isoaspartyl-specific metalloproteases [21]. These two explanations are not mutually exclusive. In our opinion, the bottom line is that NGOME is a useful tool to study the relationship between spontaneous protein deamidation and in vivo protein turnover.

## Discussion

NGOME is able to predict non-enzymatic asparagine deamidation in proteins with considerable accuracy for a sequence-based method (Fig 2). NGOME development was limited by the presence of only 39 proteins in the training dataset. The identification of deamidated asparagines within complete proteins is elusive because deamidation is a sub-daltonic modification (mass difference 0.98 Da). Moreover, deamidation is a spontaneous process that begins right after translation. Thus, the extraction of bona fide kinetic data is not obvious since the age of proteins obtained from natural sources is hard to evaluate precisely. We expect that future analyses of recombinant protein samples of known age with high resolution mass spectrometers, such as Fourier Transform Mass Spectrometry, Orbitrap Mass Spectrometry and Q-TOF, will increase the size of the training dataset and allow for improved algorithm performance. We envision the use of the current version of NGOME in proteome-wide studies of protein deamidation as well as in suggesting protein point mutations that improve the longevity of therapeutic proteins.

Enzymatic Asn deamidation, non-enzymatic deamidation at amino or carboxyl terminal Asn residues and deamidation at low pH are beyond the scope of NGOME since they take place through different mechanisms. Asparagine deamidation may be modulated by structural factors at the Asn or its proximal residues that are not included in the algorithm, such as dimerization/oligomerization, interactions with other molecules such as cofactors, glycosylation and other post-translational modifications. NGOME predictions should be used with care if such factors are suspect to be present.

Four of our case studies deal with specific proteins and show the relevance of deamidation reactions happening in different timescales and conditions (Figs 3, 4, 5 and 6). For example, superoxide dismutase deamidates slowly in the months time scale, while BCL-xL deamidates much faster in the hours to days timescale. Deamidation of BCL-xL regulates a biological process in the cell, while deamidation of Interferon beta and the Trastuzumab antibody are mainly of interest for biotechnology and in a test tube. Regardless of timescale and milieu, the algorithm yielded a relative ranking of deamidation propensity for the asparagine residues of a sequence that reflects the experimental tendencies. NGOME can also illustrate how the interplay between sequence and structure modulates the process. This valuable information can be gained in the absence of high-resolution structural data, an information that requires considerable experimental work and is absent for many proteins. Since NGOME requires only a protein sequence as an input and not a three-dimensional structure, it can be used to perform predictions at the proteome level (Fig 7). In our fifth case study, this led to testable hypotheses for the role of spontaneous asparagine deamidation in protein turnover and evolution.

## Supporting Information

S1 File File includes S1 Table and S2 Table. S1 Table: Experimental reports of spontaneous deamidation of internal Asn residues in proteins. S2 Table: Prediction of spontaneous deamidation and experimental lifetimes for all proteins in [19].
(DOCX)
