# Supplementary material for: Prediction of Spontaneous Protein Deamidation from Sequence-Derived Secondary Structure and Intrinsic Disorder
Source: PLoS One. 2015 Dec 16;10(12):e0145186. doi: 10.1371/journal.pone.0145186 (PMC4682632; doi:10.1371/journal.pone.0145186)
Supplement: S1 File — S1 Table: Experimental reports of spontaneous deamidation of internal Asn residues in proteins. S2 Table: Prediction of spontaneous deamidation and experimental lifetimes for all proteins in [19]. (DOCX) [file pone.0145186.s001.docx]

**Supplementary table 1.** Experimental reports of spontaneous deamidation of internal Asn residues in proteins.

| Protein | Sequence | Asn position | Positive/Negative | Reference |
| --- | --- | --- | --- | --- |
| Seminal RNAse | P00669 | 17 | N | [1] |
| Seminal RNAse | P00669 | 24 | N | [1] |
| Seminal RNAse | P00669 | 27 | N | [1] |
| Seminal RNAse | P00669 | 44 | N | [1] |
| Seminal RNAse | P00669 | 67 | P | [1] |
| Seminal RNAse | P00669 | 71 | N | [1] |
| Seminal RNAse | P00669 | 94 | N | [1] |
| Hemoglobin subunit alpha D6N | P69905 | 6 | N | [1] |
| Hemoglobin subunit alpha K7N | P69905 | 7 | N | [1] |
| Hemoglobin subunit alpha | P69905 | 9 | N | [1] |
| Hemoglobin subunit alpha K11N | P69905 | 11 | N | [1] |
| Hemoglobin subunit alpha K16N | P69905 | 16 | N | [1] |
| Hemoglobin subunit alpha D47N | P69905 | 47 | N | [1] |
| Hemoglobin subunit alpha H50N | P69905 | 50 | P | [1] |
| Hemoglobin subunit alpha K56N | P69905 | 56 | N | [1] |
| Hemoglobin subunit alpha K60N | P69905 | 60 | N | [1] |
| Hemoglobin subunit alpha K61N | P69905 | 61 | N | [1] |
| Hemoglobin subunit alpha D64N | P69905 | 64 | N | [1] |
| Hemoglobin subunit alpha | P69905 | 68 | N | [1] |
| Hemoglobin subunit alpha D74N | P69905 | 74 | N | [1] |
| Hemoglobin subunit alpha D75N | P69905 | 75 | N | [1] |
| Hemoglobin subunit alpha A79G | P69905 | 78 | P | [1] |
| Hemoglobin subunit alpha | P69905 | 78 | N | [1] |
| Hemoglobin subunit alpha A79T | P69905 | 78 | N | [1] |
| Hemoglobin subunit alpha D85N | P69905 | 85 | N | [1] |
| Hemoglobin subunit alpha H87N | P69905 | 87 | N | [1] |
| Hemoglobin subunit alpha K90N | P69905 | 90 | N | [1] |
| Hemoglobin subunit alpha D94N | P69905 | 94 | N | [1] |
| Hemoglobin subunit alpha | P69905 | 97 | N | [1] |
| Hemoglobin subunit alpha D126N | P69905 | 126 | N | [1] |
| Hemoglobin subunit alpha K127N | P69905 | 127 | N | [1] |
| Hemoglobin subunit alpha S133N | P69905 | 133 | N | [1] |
| Hemoglobin subunit beta K17N | P68871 | 17 | N | [1] |
| Hemoglobin subunit beta V20G | P68871 | 19 | N | [1] |
| Hemoglobin subunit beta V20E | P68871 | 19 | N | [1] |
| Hemoglobin subunit beta V20M | P68871 | 19 | N | [1] |
| Hemoglobin subunit beta | P68871 | 19 | N | [1] |
| Hemoglobin subunit beta D21N | P68871 | 21 | N | [1] |
| Hemoglobin subunit beta T38N | P68871 | 38 | N | [1] |
| Hemoglobin subunit beta D47N | P68871 | 47 | N | [1] |
| Hemoglobin subunit beta D52N | P68871 | 52 | N | [1] |
| Hemoglobin subunit beta | P68871 | 57 | N | [1] |
| Hemoglobin subunit beta P58R | P68871 | 57 | N | [1] |
| Hemoglobin subunit beta K59N | P68871 | 59 | N | [1] |
| Hemoglobin subunit beta K61N | P68871 | 61 | N | [1] |
| Hemoglobin subunit beta H63N | P68871 | 63 | N | [1] |
| Hemoglobin subunit beta K65N | P68871 | 65 | N | [1] |
| Hemoglobin subunit beta D73N | P68871 | 73 | N | [1] |
| Hemoglobin subunit beta D79N | P68871 | 79 | N | [1] |
| Hemoglobin subunit beta L81H | P68871 | 80 | N | [1] |
| Hemoglobin subunit beta | P68871 | 80 | N | [1] |
| Hemoglobin subunit beta K82N | P68871 | 82 | P | [1] |
| Hemoglobin subunit beta S89N | P68871 | 89 | N | [1] |
| Hemoglobin subunit beta D94N | P68871 | 94 | N | [1] |
| Hemoglobin subunit beta K95L | P68871 | 95 | N | [1] |
| Hemoglobin subunit beta D99N | P68871 | 99 | N | [1] |
| Hemoglobin subunit beta | P68871 | 102 | N | [1] |
| Hemoglobin subunit beta F103I | P68871 | 102 | N | [1] |
| Hemoglobin subunit beta F103L | P68871 | 102 | N | [1] |
| Hemoglobin subunit beta | P68871 | 108 | N | [1] |
| Hemoglobin subunit beta V109M | P68871 | 108 | N | [1] |
| Hemoglobin subunit beta V109L | P68871 | 108 | N | [1] |
| Hemoglobin subunit beta H117N | P68871 | 117 | N | [1] |
| Hemoglobin subunit beta K120N | P68871 | 120 | N | [1] |
| Hemoglobin subunit beta K132N | P68871 | 132 | N | [1] |
| Hemoglobin subunit beta A140D | P68871 | 139 | N | [1] |
| Hemoglobin subunit beta A140T | P68871 | 139 | N | [1] |
| Hemoglobin subunit beta A140V | P68871 | 139 | N | [1] |
| Hemoglobin subunit beta | P68871 | 139 | N | [1] |
| Hemoglobin subunit beta H143N | P68871 | 143 | N | [1] |
| Hemoglobin subunit beta K144N | P68871 | 144 | N | [1] |
| Hemoglobin subunit beta Y145N | P68871 | 145 | P | [1] |
| Aldolase | P00883 | 50 | N | [1] |
| Aldolase | P00883 | 54 | N | [1] |
| Aldolase | P00883 | 70 | N | [1] |
| Aldolase | P00883 | 119 | N | [1] |
| Aldolase | P00883 | 166 | N | [1] |
| Aldolase | P00883 | 168 | N | [1] |
| Aldolase | P00883 | 180 | N | [1] |
| Aldolase | P00883 | 231 | N | [1] |
| Aldolase | P00883 | 282 | N | [1] |
| Aldolase | P00883 | 284 | N | [1] |
| Aldolase | P00883 | 287 | N | [1] |
| Aldolase | P00883 | 319 | N | [1] |
| Aldolase | P00883 | 334 | N | [1] |
| Aldolase | P00883 | 360 | P | [1] |
| RNAse A | P61823 | 24 | N | [1] |
| RNAse A | P61823 | 27 | N | [1] |
| RNAse A | P61823 | 34 | N | [1] |
| RNAse A | P61823 | 44 | N | [1] |
| RNAse A | P61823 | 62 | N | [1] |
| RNAse A | P61823 | 67 | P | [1] |
| RNAse A | P61823 | 71 | N | [1] |
| RNAse A | P61823 | 94 | N | [1] |
| RNAse A | P61823 | 103 | N | [1] |
| RNAse A | P61823 | 113 | N | [1] |
| Angiogenin | P03950 | 3 | N | [1] |
| Angiogenin | P03950 | 43 | N | [1] |
| Angiogenin | P03950 | 49 | N | [1] |
| Angiogenin | P03950 | 59 | N | [1] |
| Angiogenin | P03950 | 61 | P | [1] |
| Angiogenin | P03950 | 63 | N | [1] |
| Angiogenin | P03950 | 68 | N | [1] |
| Angiogenin | P03950 | 102 | N | [1] |
| Angiogenin | P03950 | 109 | P | [1] |
| T-cell surface glycoprotein CD4 | P01730 | 30 | N | [1] |
| T-cell surface glycoprotein CD4 | P01730 | 32 | N | [1] |
| T-cell surface glycoprotein CD4 | P01730 | 39 | N | [1] |
| T-cell surface glycoprotein CD4 | P01730 | 52 | N | [1] |
| T-cell surface glycoprotein CD4 | P01730 | 66 | N | [1] |
| T-cell surface glycoprotein CD4 | P01730 | 73 | N | [1] |
| T-cell surface glycoprotein CD4 | P01730 | 103 | N | [1] |
| T-cell surface glycoprotein CD4 | P01730 | 137 | N | [1] |
| T-cell surface glycoprotein CD4 | P01730 | 164 | N | [1] |
| Hen lysozyme | P00698 | 19 | N | [1] |
| Hen lysozyme | P00698 | 27 | N | [1] |
| Hen lysozyme | P00698 | 37 | N | [1] |
| Hen lysozyme | P00698 | 39 | N | [1] |
| Hen lysozyme | P00698 | 44 | N | [1] |
| Hen lysozyme | P00698 | 46 | N | [1] |
| Hen lysozyme | P00698 | 59 | N | [1] |
| Hen lysozyme | P00698 | 65 | N | [1] |
| Hen lysozyme | P00698 | 74 | N | [1] |
| Hen lysozyme | P00698 | 77 | N | [1] |
| Hen lysozyme | P00698 | 93 | N | [1] |
| Hen lysozyme | P00698 | 103 | P | [1] |
| Hen lysozyme | P00698 | 106 | N | [1] |
| Hen lysozyme | P00698 | 113 | N | [1] |
| HPR | P0AA04 | 12 | P | [1] |
| HPR | P0AA04 | 38 | P | [1] |
| HisF | P60714 | 11 | N | [1] |
| HisF | P60714 | 15 | P | [1] |
| HisF | P60714 | 29 | N | [1] |
| HisF | P60714 | 65 | N | [1] |
| HisF | P60714 | 71 | P | [1] |
| HisF | P60714 | 153 | N | [1] |
| HisF | P60714 | 195 | N | [1] |
| HisF | P60714 | 245 | N | [1] |
| FABP | P02692 | 2 | N | [1] |
| FABP | P02692 | 14 | N | [1] |
| FABP | P02692 | 61 | N | [1] |
| FABP | P02692 | 89 | N | [1] |
| FABP | P02692 | 105 | P | [1] |
| FABP | P02692 | 111 | N | [1] |
| RNAse U2 | P00654 | 8 | N | [1] |
| RNAse U2 | P00654 | 12 | N | [1] |
| RNAse U2 | P00654 | 16 | N | [1] |
| RNAse U2 | P00654 | 20 | N | [1] |
| RNAse U2 | P00654 | 32 | P | [1] |
| RNAse U2 | P00654 | 38 | N | [1] |
| RNAse U2 | P00654 | 68 | N | [1] |
| RNAse U2 | P00654 | 77 | N | [1] |
| RNAse U2 | P00654 | 91 | N | [1] |
| FGF | P05230 | 2 | N | [1] |
| FGF | P05230 | 7 | P | [1] |
| FGF | P05230 | 18 | N | [1] |
| CytC | P00004 | 31 | N | [1] |
| CytC | P00004 | 52 | N | [1] |
| CytC | P00004 | 70 | N | [1] |
| CytC | P00004 | 103 | P | [1] |
| Interleukin 1beta | P10749 | 32 | P | [1] |
| Interleukin 1beta | P10749 | 35 | N | [1] |
| Interleukin 1beta | P10749 | 37 | N | [1] |
| Interleukin 1beta | P10749 | 53 | N | [1] |
| Interleukin 1beta | P10749 | 66 | N | [1] |
| Interleukin 1beta | P10749 | 102 | N | [1] |
| Interleukin 1beta | P10749 | 119 | N | [1] |
| Interleukin 1beta | P10749 | 136 | N | [1] |
| Interleukin 1beta | P10749 | 137 | N | [1] |
| Interleukin 2 | P60568 | 26 | N | [1] |
| Interleukin 2 | P60568 | 29 | N | [1] |
| Interleukin 2 | P60568 | 30 | N | [1] |
| Interleukin 2 | P60568 | 33 | N | [1] |
| Interleukin 2 | P60568 | 71 | N | [1] |
| Interleukin 2 | P60568 | 77 | N | [1] |
| Interleukin 2 | P60568 | 88 | N | [1] |
| Interleukin 2 | P60568 | 90 | N | [1] |
| Interleukin 2 | P60568 | 119 | N | [1] |
| Calbindin D9k | P02633 | 21 | N | [1] |
| Calbindin D9k | P02633 | 56 | P | [1] |
| Phenylalanine hydroxylase | P00439 | 32 | P | [2] |
| Phenylalanine hydroxylase | P00439 | 167 | N | [2] |
| Phenylalanine hydroxylase | P00439 | 207 | N | [2] |
| Phenylalanine hydroxylase | P00439 | 393 | N | [2] |
| Phenylalanine hydroxylase | P00439 | 426 | N | [2] |
| Human superoxide dismutase | P00441 | 20 | N | [3] |
| Human superoxide dismutase | P00441 | 27 | P | [3] |
| Human superoxide dismutase | P00441 | 54 | N | [3] |
| Human superoxide dismutase | P00441 | 66 | N | [3] |
| Human superoxide dismutase | P00441 | 87 | N | [3] |
| Human superoxide dismutase | P00441 | 132 | N | [3] |
| Human superoxide dismutase | P00441 | 140 | N | [3] |
| Growth hormone | P01241 | 38 | N | [4] |
| Growth hormone | P01241 | 73 | N | [4] |
| Growth hormone | P01241 | 89 | N | [4] |
| Growth hormone | P01241 | 98 | N | [4] |
| Growth hormone | P01241 | 125 | N | [4] |
| Growth hormone | P01241 | 135 | N | [4] |
| Growth hormone | P01241 | 175 | P | [4] |
| Growth hormone | P01241 | 178 | N | [4] |
| Growth hormone | P01241 | 185 | N | [4] |
| Calbindin D28k | P05937 | 38 | N | [5] |
| Calbindin D28k | P05937 | 87 | N | [5] |
| Calbindin D28k | P05937 | 125 | N | [5] |
| Calbindin D28k | P05937 | 135 | N | [5] |
| Calbindin D28k | P05937 | 157 | N | [5] |
| Calbindin D28k | P05937 | 158 | N | [5] |
| Calbindin D28k | P05937 | 176 | N | [5] |
| Calbindin D28k | P05937 | 192 | N | [5] |
| Calbindin D28k | P05937 | 203 | P | [5] |
| Calbindin D28k | P05937 | 209 | N | [5] |
| Calbindin D28k | P05937 | 222 | N | [5] |
| Calbindin D28k | P05937 | 229 | N | [5] |
| Calbindin D28k | P05937 | 230 | N | [5] |
| Calbindin D28k | P05937 | 237 | N | [5] |
| SHMT1 | P07511 | 6 | P | [6] |
| SHMT1 | P07511 | 221 | P | [6] |
| Mouse superoxide dismutase | P09671 | 91 | P | [7] |
| Gelsolin | P13020 | 303 | P | [7] |
| Protective antigen | P13423 | 10 | N | [8] |
| Protective antigen | P13423 | 150 | N | [8] |
| Protective antigen | P13423 | 162 | P | [8] |
| Protective antigen | P13423 | 306 | N | [8] |
| Protective antigen | P13423 | 705 | N | [8] |
| Protective antigen | P13423 | 709 | N | [8] |
| Protective antigen | P13423 | 713 | P | [8] |
| Protective antigen | P13423 | 719 | P | [8] |
| Glyceraldehyde-3-phosphate dehydrogenase | P16858 | 7 | P | [7] |
| Glyceraldehyde-3-phosphate dehydrogenase | P16858 | 62 | P | [7] |
| Glyceraldehyde-3-phosphate dehydrogenase | P16858 | 68 | P | [7] |
| Mouse triosephosphate isomerase | P17751 | 122 | P | [7] |
| Glutathione S-transferase P1 | P19157 | 114 | P | [7] |
| Glutathione S-transferase P1 | P19157 | 205 | P | [7] |
| Protein disulfide-isomerase A3 | P27773 | 199 | P | [7] |
| Alpha-synuclein | P37840 | 65 | N | [9] |
| Alpha-synuclein | P37840 | 103 | P | [9] |
| Alpha-synuclein | P37840 | 122 | P | [9] |
| Alpha-synuclein L8N | P37840 | 8 | P | [9] |
| Alpha-synuclein Q24N | P37840 | 24 | P | [9] |
| Alpha-synuclein A30N | P37840 | 30 | P | [9] |
| Alpha-synuclein V40N | P37840 | 40 | P | [9] |
| Alpha-synuclein V66G | P37840 | 65 | P | [9] |
| Alpha-synuclein V49N | P37840 | 49 | P | [9] |
| Alpha-synuclein V66H | P37840 | 65 | P | [9] |
| Alpha-synuclein V66S | P37840 | 65 | P | [9] |
| Alpha-synuclein T72N | P37840 | 72 | P | [9] |
| Alpha-synuclein L100N | P37840 | 100 | P | [9] |
| Alpha-synuclein Q109N | P37840 | 109 | P | [9] |
| Alpha-synuclein I112N L113G | P37840 | 112 | P | [9] |
| Alpha-synuclein A140NGA | P37840 | 140 | P | [9] |
| Interleukin-15 | P40933 | 125 | P | [10] |
| Beta-2-microglobulin | P61769 | 37 | P | [11] |
| Beta-2-microglobulin | P61769 | 62 | N | [11] |
| VATB2 | P62814 | 11 | P | [7] |
| Canine lysozyme | P81708 | 27 | N | [12] |
| Canine lysozyme | P81708 | 37 | N | [12] |
| Canine lysozyme | P81708 | 39 | N | [12] |
| Canine lysozyme | P81708 | 44 | P | [12] |
| Canine lysozyme | P81708 | 47 | P | [12] |
| Canine lysozyme | P81708 | 49 | P | [12] |
| Canine lysozyme | P81708 | 60 | N | [12] |
| Canine lysozyme | P81708 | 68 | P | [12] |
| Canine lysozyme | P81708 | 74 | N | [12] |
| Canine lysozyme | P81708 | 77 | N | [12] |
| Canine lysozyme | P81708 | 87 | N | [12] |
| Canine lysozyme | P81708 | 103 | N | [12] |
| Canine lysozyme | P81708 | 128 | N | [12] |
| Ubiquitin-like modifier-activating enzyme 1 | Q02053 | 44 | P | [7] |
| Bcl-2-like protein 1 | Q07817 | 5 | N | [13] |
| Bcl-2-like protein 1 | Q07817 | 33 | N | [13] |
| Bcl-2-like protein 1 | Q07817 | 52 | P | [13] |
| Bcl-2-like protein 1 | Q07817 | 54 | N | [13] |
| Bcl-2-like protein 1 | Q07817 | 66 | P | [13] |
| Bcl-2-like protein 1 | Q07817 | 128 | N | [13] |
| Bcl-2-like protein 1 | Q07817 | 136 | N | [13] |
| Bcl-2-like protein 1 | Q07817 | 175 | N | [13] |
| Bcl-2-like protein 1 | Q07817 | 185 | N | [13] |
| Serpin B6 | Q60854 | 65 | P | [7] |
| Ethanolamine-phosphate cytidyltransferase | Q922E4 | 4 | P | [7] |
| Cytochrome b-c1 complex subunit 1 | Q9CZ13 | 87 | P | [7] |
| Tropomodulin-2 | Q9JKK7 | 155 | P | [7] |

**Supplementary table 2.** Prediction of spontaneous deamidation and experimental lifetimes for all proteins in [14].

| **Protein** | **t_50_(NGOME) (days)** | **t_50_(sequence) (days)** | **t_50_(in vivo) (minutes)** |
| --- | --- | --- | --- |
| YBR215W | 0.9 | 0.2 | 16 |
| YPL268W | 1.4 | 0.2 | 3 |
| YPR124W | 8.9 | 1.7 | 80 |
| YBR105C | 1.5 | 0.3 | -3982 |
| YBR170C | 1.8 | 0.3 | 29 |
| YJR080C | 2.4 | 0.3 | 81 |
| YMR069W | 7.1 | 0.6 | 30 |
| YNL083W | 4.4 | 0.2 | 26 |
| YLL062C | 12.9 | 0.8 | 463 |
| YPL232W | 22.7 | 2.9 | 14 |
| YJR103W | 17.3 | 2.2 | 32 |
| YOR216C | 10 | 1.5 | 8 |
| YOR284W | 10.3 | 0.9 | 97 |
| YDL126C | 2.6 | 0.4 | 34 |
| YDR307W | 8.3 | 0.7 | 29 |
| YPL183W-A | 212.2 | 21 | 2298 |
| YER182W | 7.1 | 0.5 | 71 |
| YGL036W | 0.7 | 0.1 | 6 |
| YNL225C | 1.9 | 0.4 | 29 |
| YBR221C | 5.8 | 0.5 | 72 |
| YDL184C |  |  | 34 |
| YLR245C | 85.6 | 5.1 | -419 |
| YHR039C | 8.4 | 0.5 | 38 |
| YJL011C | 16.5 | 3.2 | 319 |
| YJR101W | 3 | 0.3 | 93 |
| YDR356W | 1.1 | 0.2 | 36 |
| YGR266W | 6.1 | 0.4 | 30 |
| YNL072W | 32.6 | 4.1 | 49 |
| YNL239W | 21.6 | 1.9 | 20 |
| YML094W | 123.4 | 10.9 | 51 |
| YOL096C | 85.6 | 4.9 | 3 |
| YEL022W | 1.4 | 0.3 | 16 |
| YLR163C | 2.5 | 0.3 | 1961 |
| YMR108W | 20.3 | 1.8 | 40 |
| YCR068W | 9.7 | 0.8 | 12 |
| YDR421W | 2.2 | 0.3 | 4 |
| YDR259C | 1.6 | 0.4 | 21 |
| YBR262C | 18.8 | 1.1 | -127 |
| YLR260W | 2.1 | 0.2 | 3 |
| YHR068W | 13.7 | 0.9 | 218 |
| YLR138W | 1.6 | 0.2 | -299 |
| YBR127C | 2.1 | 0.2 | 29 |
| YBR011C | 34.5 | 3.5 | 883 |
| YMR072W | 27.1 | 6.6 | 91 |
| YDR261C | 3.3 | 0.3 | 11 |
| YPR062W | 102.5 | 10.5 | -202 |
| YDR098C | 5.2 | 0.8 | 226 |
| YMR220W | 9.8 | 0.9 | 92 |
| YKR066C | 1.8 | 0.3 | -410 |
| YPR018W | 2.9 | 0.7 | 24 |
| YHR127W | 2.5 | 0.5 | -914 |
| YMR275C | 1.5 | 0.3 | 10 |
| YGL177W | 138.1 | 26.4 | 25 |
| YDR190C | 20.5 | 2.9 | -601 |
| YOL140W | 6.1 | 0.5 | 81 |
| YPL016W | 0.5 | 0.1 | 6 |
| YJR058C | 273.8 | 20.1 | 145 |
| YJL024C | 28.4 | 3.4 | 2108 |
| YBR106W | 36.1 | 7.5 | 101 |
| YGL037C | 29.7 | 4.8 | -97 |
| YMR276W | 2.1 | 0.4 | 50 |
| YJL217W | 11.2 | 1.1 | 99 |
| YMR195W | 4.9 | 0.9 | 42 |
| YDR347W | 18.4 | 2.1 | 44 |
| YIL097W | 1.3 | 0.3 | 59 |
| YLR003C | 10.7 | 0.9 | 31 |
| YGR177C | 6.8 | 0.4 | 118 |
| YKR076W | 3.6 | 0.5 | 39 |
| YPL225W | 44.1 | 8 | 34 |
| YER156C | 5.8 | 0.8 | -185 |
| YEL063C | 2.8 | 0.3 | 29 |
| YDR520C | 1 | 0.2 | 16 |
| YBR007C | 1.1 | 0.3 | 20 |
| YLL010C | 5.4 | 1.7 | 758 |
| YOR004W | 1.5 | 0.8 | -212 |
| YDL168W | 12.1 | 1 | 48 |
| YJR033C | 1.6 | 0.1 | -3757 |
| YOR171C | 2.3 | 0.3 | 47 |
| YMR225C | 45.4 | 6.1 | 51 |
| YGL180W | 2.6 | 0.4 | 47 |
| YPL149W | 5.7 | 0.4 | -629 |
| YFR028C | 0.6 | 0.2 | 20 |
| YLL054C | 3.5 | 0.2 | 45 |
| YOR073W | 1.7 | 0.3 | 11 |
| YLR378C | 7.8 | 1 | 174 |
| YNL138W | 9.5 | 1.7 | 87 |
| YLR253W | 5.5 | 0.3 | 53 |
| YKR079C | 2.8 | 0.3 | 48 |
| YGR072W | 3.2 | 1 | 33 |
| YPL061W | 2.7 | 0.3 | 115 |
| YLR049C | 6.5 | 0.9 | 11 |
| YFL007W | 1.6 | 0.1 | 6 |
| YNR013C | 2.6 | 0.6 | 37 |
| YDR485C | 1.1 | 0.2 | 1855 |
| YJL080C | 2.5 | 0.3 | 183 |
| YGR188C | 1.3 | 0.2 | 22 |
| YLR133W | 2.6 | 0.3 | 64 |
| YJL047C | 2.1 | 0.3 | 7 |
| YGL245W | 3.1 | 0.3 | 53 |
| YKL216W | 4.2 | 0.3 | 203 |
| YML118W | 1.8 | 0.3 | 68 |
| YFL034C-A | 7.6 | 1.1 | -244 |
| YDR005C | 1.2 | 0.4 | 31 |
| YBR135W | 44.2 | 8.4 | 52 |
| YNL265C | 8.8 | 0.8 | 138 |
| YDL112W | 3.5 | 0.2 | 18 |
| YNL162W-A | 201.5 | 9.8 | 17 |
| YEL070W | 3.1 | 0.3 | 227 |
| YDR367W | 71.1 | 7 | 173 |
| YDR293C | 0.6 | 0.2 | 18 |
| YDR116C | 31.4 | 3.8 | 47 |
| YOR342C | 6.9 | 0.7 | 15 |
| YGL181W | 18.6 | 2.4 | 63 |
| YDR469W | 2 | 0.7 | 179 |
| YHR109W | 4.8 | 0.4 | 19 |
| YDR234W | 10.2 | 1 | 40 |
| YCR057C | 1.7 | 0.3 | -332 |
| YJL136C | 46.2 | 10 | 136 |
| YEL052W | 5.2 | 0.5 | 65 |
| YDR266C | 4.4 | 1 | 22 |
| YNL077W | 1.2 | 0.2 | 20 |
| YGR251W | 20.3 | 4.8 | 61 |
| YGR216C | 7.5 | 0.6 | 102 |
| YML116W | 25.3 | 2.7 | 19 |
| YOL108C | 2.9 | 1 | 42 |
| YLR221C | 1.8 | 0.5 | 83 |
| YDR453C | 8.4 | 0.9 | 149 |
| YMR193W | 11.2 | 1 | 433 |
| YIL030C | 2.3 | 0.3 | 6 |
| YNL006W | 19.1 | 2.9 | 37 |
| YFL028C | 4.8 | 0.4 | 219 |
| YGR095C | 9.9 | 0.6 | 101 |
| YGL212W | 9.1 | 2.4 | 61 |
| YDL058W | 1.4 | 0.2 | 66 |
| YKL173W | 1.8 | 0.2 | 52 |
| YDL061C | 328.2 | 26.3 | -183 |
| YPL127C | 8.6 | 1 | 35 |
| YNL053W | 8.3 | 2 | 15 |
| YOL058W | 4.9 | 0.4 | 135 |
| YIL079C | 1 | 0.3 | 29 |
| YML110C | 6.8 | 0.9 | 73 |
| YOL092W | 15.1 | 2.5 | 13 |
| YKL162C | 2 | 0.3 | 16 |
| YPL219W | 1.9 | 0.5 | 14 |
| YLR074C | 52.8 | 7.8 | 49 |
| YLR396C | 3.9 | 0.3 | 34 |
| YDR093W | 0.7 | 0.1 | 19 |
| YMR012W | 1.6 | 0.2 | 44 |
| YOR212W | 7.1 | 0.7 | 74 |
| YMR252C | 37.4 | 5.5 | -157 |
| YIL137C | 3.5 | 0.3 | 36 |
| YOR177C | 7.1 | 1 | 37 |
| YDR147W | 3.5 | 0.4 | 37 |
| YKR027W | 9.2 | 0.5 | 20 |
| YGR287C | 3.3 | 0.4 | 4816 |
| YBL042C | 9.5 | 0.7 | -526 |
| YLL013C | 0.6 | 0.1 | 326 |
| YOL082W | 4.9 | 0.6 | 41 |
| YPR067W | 13.6 | 0.9 | 18 |
| YBR208C | 0.9 | 0.1 | 45 |
| YHL007C | 0.6 | 0.2 | -147 |
| YER126C | 22.7 | 7 | 70 |
| YGL248W | 9.6 | 0.9 | 95 |
| YJR091C | 0.5 | 0.1 | 43 |
| YKL011C | 6.8 | 0.7 | 65 |
| YPR065W | 4.9 | 1.1 | 52 |
| YMR100W | 3.9 | 0.8 | 74 |
| YNR010W | 5.5 | 1 | 30 |
| YNL323W | 9.4 | 0.7 | 17 |
| YNL008C | 3.7 | 0.3 | 44 |
| YDR027C | 1.3 | 0.3 | 29 |
| YPL194W | 2 | 0.3 | 16 |
| YIL115C | 0.7 | 0.2 | 82 |
| YCL016C | 8.2 | 0.8 | 63 |
| YML011C | 172.3 | 10.4 | 4 |
| YMR014W | 5.8 | 1.2 | 98 |
| YJR051W | 1.5 | 0.3 | 18 |
| YPL031C | 10.2 | 0.9 | 217 |
| YBR097W | 1.5 | 0.2 | 2 |
| YMR283C | 22.4 | 2 | 52 |
| YKL208W | 6.3 | 0.8 | 44 |
| YNR046W | 16.1 | 1 | 36 |
| YLR071C | 0.6 | 0.1 | 61 |
| YLR250W | 3.7 | 1 | 26 |
| YOR278W | 14.6 | 1 | 89 |
| YJR075W | 17.7 | 1.8 | 8 |
| YLR068W | 58.6 | 15.5 | 34 |
| YFR050C | 4.1 | 0.3 | -91 |
| YNL079C | 12.7 | 3.4 | 153 |
| YIR034C | 62.9 | 5.9 | -214 |
| YGR124W | 9.3 | 0.8 | 41 |
| YNL158W | 90.1 | 6.5 | 49 |
| YIR008C | 3.1 | 0.7 | 60 |
| YOR193W | 16.6 | 1.7 | 42 |
| YER103W | 1.4 | 0.3 | -186 |
| YGL167C | 1.9 | 0.4 | 57 |
| YLR183C | 6 | 0.6 | 17 |
| YLR278C | 0.3 | 0.1 | 25 |
| YMR098C | 3.7 | 0.4 | -124 |
| YLR375W | 1.6 | 0.4 | 20 |
| YOR237W | 5.9 | 0.5 | 25 |
| YPR063C | 46.9 | 7.7 | 24 |
| YML113W | 2.4 | 0.6 | 42 |
| YGL060W | 4.7 | 0.4 | 45 |
| YBR225W | 1.1 | 0.3 | 9 |
| YNL249C | 4 | 0.3 | 90 |
| YKL134C | 3.4 | 0.3 | 37 |
| YDL130W | 192.1 | 18 | 395 |
| YBR289W | 0.8 | 0.2 | 3 |
| YOR227W | 2.9 | 0.4 | 3 |
| YGL020C | 22 | 0.9 | 18 |
| YLR172C | 69.4 | 4.1 | 403 |
| YLR435W | 79 | 6.2 | 27 |
| YIL039W | 3.3 | 0.3 | 70 |
| YML115C | 1.3 | 0.2 | 2 |
| YNR045W | 4.2 | 0.4 | 68 |
| YPL158C | 1.4 | 0.3 | 2 |
| YOR038C | 1.1 | 0.2 | 29 |
| YGR271W | 2.4 | 0.2 | 48 |
| YDL203C | 1 | 0.2 | 24 |
| YKL116C | 2.3 | 0.4 | 58 |
| YGR122W | 13.9 | 1.4 | 138 |
| YDR257C | 9.7 | 0.7 | -340 |
| YGR094W | 2.4 | 0.3 | 68 |
| YOR245C | 8.2 | 0.8 | 41 |
| YMR169C | 16.8 | 0.8 | 34 |
| YJR049C | 0.7 | 0.2 | 16 |
| YOR213C | 17 | 2.3 | 68 |
| YMR121C | 19.7 | 4.2 | 142 |
| YPL020C | 2.9 | 0.3 | 21 |
| YDR513W | 2.6 | 0.4 | -209 |
| YGL232W | 1.6 | 0.8 | 103 |
| YML093W | 0.6 | 0.2 | 9 |
| YER080W | 5.4 | 0.6 | 40 |
| YHR012W | 2.2 | 0.3 | 87 |
| YER021W | 2.8 | 0.4 | 23 |
| YBL060W | 3.3 | 0.3 | 22 |
| YPL018W | 3.2 | 0.4 | -116 |
| YDL080C | 5.2 | 0.4 | 33 |
| YBL005W | 6.6 | 0.8 | 51 |
| YLR239C | 6.8 | 0.9 | -83 |
| YDL167C | 0.3 | 0.1 | 20 |
| YOR155C | 13.8 | 0.9 | 50 |
| YER068W | 2.6 | 0.4 | 25 |
| YMR308C | 6.4 | 0.4 | 88 |
| YOR136W | 12 | 0.8 | -364 |
| YOR337W | 1.5 | 0.3 | 4 |
| YOR304W | 1.2 | 0.2 | 16 |
| YDL103C | 2 | 0.3 | 60 |
| YNL248C | 23.8 | 2.7 | 113 |
| YGR286C | 4.6 | 0.5 | 31 |
| YGR031W | 6.5 | 0.8 | -1024 |
| YOL090W | 9.4 | 0.8 | 32 |
| YGR280C | 3.5 | 0.5 | 110 |
| YOR069W | 2.2 | 0.3 | 18 |
| YPR079W | 2.2 | 0.3 | 252 |
| YDR055W | 3.7 | 0.3 | 20 |
| YMR168C | 15.7 | 1.4 | 22 |
| YBR061C | 110.7 | 8 | 39 |
| YCL057W | 3.1 | 0.2 | 63 |
| YLR373C | 1.9 | 0.4 | 22 |
| YNL281W | 17 | 1.1 | -443 |
| YOL059W | 9.5 | 1 | 116 |
| YEL008W | 96.7 | 6.3 | 341 |
| YJL087C | 4.2 | 0.3 | 21 |
| YLR387C | 8.5 | 0.7 | 43 |
| YDL135C | 30.5 | 3.3 | -83 |
| YLR298C | 2.7 | 0.8 | 74 |
| YLR328W | 15.6 | 2.7 | 60 |
| YNL141W | 14 | 0.9 | 82 |
| YDR389W | 0.8 | 0.2 | 16 |
| YBR120C | 66 | 5.8 | 935 |
| YMR244C-A | 36.8 | 7.4 | 33 |
| YLR421C | 19.7 | 3.7 | -102 |
| YMR004W | 3.4 | 0.6 | 18 |
| YLR222C | 3 | 0.2 | 53 |
| YLR166C | 4.1 | 0.4 | 58 |
| YOR060C | 51.7 | 3.6 | 21 |
| YDR323C | 2.8 | 0.4 | 3 |
| YLR367W | 14.8 | 1 | 181 |
| YLR177W | 1.1 | 0.2 | 13 |
| YOR374W | 2.2 | 0.3 | 41 |
| YFR005C | 3.3 | 0.4 | 7 |
| YKL080W | 27.2 | 2.1 | 53 |
| YML076C | 1.2 | 0.2 | 3 |
| YOL137W | 14.5 | 2.1 | 129 |
| YIL001W | 7.2 | 0.5 | 24 |
| YAL041W | 1.2 | 0.3 | 73 |
| YDR096W | 0.3 | 0.1 | 28 |
| YOR355W | 0.8 | 0.2 | 5 |
| YEL061C | 0.6 | 0.1 | 25 |
| YJL073W | 2.4 | 0.4 | 21 |
| YHR175W | 16.1 | 0.9 | -261 |
| YLR424W | 5.7 | 0.6 | 28 |
| YGR030C | 2.6 | 0.5 | 27 |
| YER005W | 5.7 | 0.4 | 35 |
| YBR160W | 120.2 | 10.6 | -74 |
| YGL039W | 7.2 | 0.5 | 30 |
| YLL007C | 4.7 | 0.4 | 41 |
| YOR158W | 1.6 | 0.3 | 54 |
| YDL229W | 11.4 | 0.9 | 149 |
| YLR363C | 2.3 | 0.5 | 93 |
| YLL058W | 5.7 | 0.4 | 38 |
| YMR110C | 3.9 | 0.4 | 321 |
| YJL208C | 17.6 | 2.7 | 40 |
| YOR057W | 4.6 | 0.8 | 34 |
| YPL097W | 4.5 | 0.3 | 33 |
| YBL074C | 19.7 | 2 | 250 |
| YLR357W | 2.7 | 0.5 | 23 |
| YLR407W | 13.1 | 2.6 | 46 |
| YLR192C | 3.1 | 1 | 36 |
| YKL079W | 5 | 0.4 | 21 |
| YJL084C | 0.9 | 0.2 | 3 |
| YNL063W | 16.2 | 1 | 92 |
| YMR278W | 1.9 | 0.2 | 26 |
| YAL022C | 1.7 | 0.2 | 3 |
| YOL110W | 21 | 1 | 20 |
| YPL220W | 53.1 | 4.3 | 76 |
| YLR020C | 2.5 | 0.4 | 61 |
| YEL009C | 19.8 | 3.8 | 5 |
| YCL033C | 23.5 | 3.4 | -97 |
| YOR264W | 4.2 | 0.8 | 36 |
| YDR277C | 4.1 | 0.6 | 28 |
| YGR024C | 30.4 | 2.6 | -653 |
| YOL023W | 8.4 | 0.6 | 11 |
| YDR484W | 3.2 | 0.2 | 29 |
| YPR114W | 91.6 | 6.2 | 27 |
| YPL174C | 1.8 | 0.2 | 10 |
| YGR140W | 3.3 | 0.4 | 101 |
| YDL013W | 0.6 | 0.2 | 17 |
| YKR026C | 22.2 | 0.9 | -73 |
| YPL046C | 1241.9 | 49.7 | 5 |
| YLR137W | 8.7 | 1 | 48 |
| YLR104W | 18.6 | 4.4 | 85 |
| YAR002C-A | 57.5 | 4.2 | 33 |
| YBR034C | 3.6 | 0.4 | 43 |
| YJR050W | 7.4 | 0.8 | 17 |
| YLR352W | 2.8 | 0.5 | 7 |
| YHL030W | 3.7 | 0.3 | 19 |
| YOR288C | 8 | 0.8 | 20 |
| YOR130C | 28.7 | 1 | 23 |
| YNL201C | 1.1 | 0.2 | 137 |
| YHR122W | 30.6 | 5.9 | 357 |
| YOR310C | 34.2 | 2.3 | 38 |
| YNL099C | 54.7 | 7.5 | 42 |
| YOR143C | 10.8 | 0.7 | 82 |
| YKL139W | 0.7 | 0.3 | 22 |
| YLR100W | 34.5 | 2.4 | 32 |
| YMR175W | 10.8 | 4.5 | 34 |
| YGR005C | 5.1 | 1.4 | -244 |
| YHR131C | 2.3 | 0.5 | 39 |
| YDL197C | 9.5 | 1.4 | 24 |
| YBL025W | 84.5 | 8 | 411 |
| YMR001C | 4 | 0.3 | 10 |
| YGR129W | 22.4 | 3.9 | 81 |
| YDR480W | 2.4 | 0.5 | 40 |
| YMR111C | 1.8 | 0.7 | 10 |
| YJL061W | 5.6 | 0.4 | 81 |
| YMR125W | 2.8 | 0.3 | 65 |
| YPL216W | 1.4 | 0.1 | 52 |
| YDR111C | 34.4 | 3.5 | 35 |
| YJL049W | 5 | 0.6 | 66 |
| YEL006W | 1.1 | 0.5 | 10 |
| YPL227C | 61.9 | 5.1 | -121 |
| YOR305W | 7.9 | 0.8 | 25 |
| YPL204W | 1.2 | 0.6 | 25 |
| YNL282W | 95.1 | 5.4 | 51 |
| YOR241W | 7.6 | 0.7 | 99 |
| YLR088W | 7.4 | 0.7 | 52 |
| YOR076C | 3.7 | 0.4 | 20 |
| YJL056C | 0.8 | 0.2 | 163 |
| YDL216C | 3.8 | 0.7 | 24 |
| YMR216C | 0.9 | 0.2 | 62 |
| YDR032C | 128.7 | 8.8 | 40 |
| YML026C | 89.7 | 8.8 | 321 |
| YML078W | 3.8 | 0.4 | 58 |
| YER007C-A | 78.1 | 7.6 | 74 |
| YHL001W | 708.7 | 55.8 | 163 |
| YNL035C | 4.6 | 0.5 | 72 |
| YLR336C | 1.2 | 0.3 | 51 |
| YMR172W | 0.6 | 0.2 | 3 |
| YOR196C | 12 | 2.1 | 69 |
| YDL052C | 23.4 | 3.1 | 138 |
| YAL031C | 1.6 | 0.3 | 17 |
| YLL014W | 79.7 | 11.4 | 20 |
| YJR052W | 4.9 | 0.3 | 37 |
| YDR127W | 1.7 | 0.1 | 60 |
| YHR044C | 4.9 | 0.6 | 18 |
| YDL091C | 1 | 0.2 | 7 |
| YOR289W | 10.4 | 1 | -152 |
| YMR142C | 8.8 | 1 | 67 |
| YMR247C | 1.4 | 0.1 | 3 |
| YLR452C | 3.4 | 0.4 | 60 |
| YHR003C | 20.2 | 2.7 | 122 |
| YGL190C | 1 | 0.2 | 80 |
| YGL101W | 15.4 | 1.1 | 51 |
| YOR095C | 42 | 4 | -233 |
| YOR202W | 4.3 | 1.1 | 14 |
| YKL122C | 1.8 | 0.8 | 325 |
| YHR167W | 50.6 | 3.9 | 30 |
| YGL099W | 2.3 | 0.3 | 44 |
| YGL098W | 42.3 | 8.4 | 34 |
| YML062C | 4.2 | 0.8 | 43 |
| YPL133C | 2.1 | 0.7 | 12 |
| YNL185C | 206.2 | 16.6 | 169 |
| YGL049C | 0.5 | 0.2 | 43 |
| YDR329C | 24 | 1.9 | 2 |
| YHR005C | 14.8 | 1.8 | 71 |
| YDL017W | 1.8 | 0.3 | 35 |
| YGL127C | 1.3 | 0.6 | 911 |
| YGR097W | 0.8 | 0.2 | 29 |
| YGL058W | 15.1 | 1.1 | 114 |
| YKR016W | 7.3 | 0.5 | 176 |
| YDL131W | 3.5 | 0.5 | 450 |
| YIL071C | 10.9 | 0.7 | 33 |
| YNR051C | 0.9 | 0.2 | 18 |
| YCL029C | 2.7 | 0.8 | 77 |
| YDR318W | 11.3 | 0.8 | 104 |
| YMR239C | 2.1 | 0.4 | 103 |
| YBL103C | 2.5 | 0.7 | 36 |
| YOR153W | 1.7 | 0.2 | 89 |
| YOR272W | 17 | 1.7 | 52 |
| YJL148W | 5.5 | 1 | -525 |
| YNL287W | 3 | 0.3 | 37 |
| YNL071W | 8.4 | 0.8 | 52 |
| YLR321C | 1 | 0.2 | 5 |
| YAL016W | 19.7 | 1.7 | 180 |
| YLR225C | 2.6 | 0.3 | 167 |
| YML117W | 0.3 | 0.1 | 139 |
| YDL159W | 1.9 | 0.3 | 14 |
| YPR024W | 4.7 | 0.7 | 52 |
| YKL213C | 2.2 | 0.2 | 23 |
| YJL023C | 4.9 | 0.5 | 67 |
| YLR451W | 3.1 | 0.4 | 12 |
| YGR233C | 1.2 | 0.1 | 41 |
| YJR083C | 2.6 | 0.7 | 57 |
| YDR047W | 42.8 | 6.3 | 845 |
| YLR291C | 7.3 | 0.7 | 124 |
| YNL010W | 192 | 12.3 | 97 |
| YPR185W | 0.9 | 0.2 | 14 |
| YIL026C | 2.9 | 0.6 | 42 |
| YML029W | 3.1 | 0.3 | 18 |
| YPL138C | 9 | 1 | 40 |
| YDL113C | 1.1 | 0.2 | 55 |
| YMR316W | 4.1 | 0.5 | 20 |
| YBR016W | 13.4 | 5.5 | 98 |
| YOR175C | 3.5 | 0.3 | -247 |
| YOR140W | 2 | 0.5 | 23 |
| YNL087W | 1.1 | 0.2 | 19 |
| YBR133C | 5.5 | 0.5 | 14 |
| YJR149W | 6.8 | 0.5 | 93 |
| YLR119W | 62.3 | 8.1 | 66 |
| YMR070W | 0.5 | 0.2 | 5 |
| YLR045C | 3.6 | 0.5 | 19 |
| YML052W | 24.8 | 2.9 | 207 |
| YLR326W | 1.1 | 0.5 | 115 |
| YOR361C | 3.4 | 0.4 | 51 |
| YPL089C | 0.2 | 0.1 | 32 |
| YNL236W | 2.3 | 0.2 | -293 |
| YGR167W | 1 | 0.4 | 23 |
| YBR251W | 10.9 | 0.9 | 201 |
| YBR173C | 8.8 | 1.1 | 17 |
| YGL247W | 112.5 | 4.7 | -131 |
| YBR095C | 1.8 | 0.4 | 72 |
| YLR197W | 31.9 | 1.9 | 18 |
| YEL050C | 12.2 | 3.1 | 79 |
| YHR207C | 1 | 0.2 | 793 |
| YJL060W | 15 | 1.6 | 32 |
| YGR235C | 43.4 | 4.2 | 85 |
| YHR194W | 3.3 | 0.2 | 10 |
| YDR408C | 164.5 | 10.9 | 346 |
| YGL129C | 4.3 | 0.3 | 69 |
| YBR271W | 31.2 | 2.2 | 80 |
| YLL061W | 12.4 | 0.8 | 2 |
| YDR422C | 0.8 | 0.2 | 23 |
| YGL057C | 3.1 | 0.5 | 151 |
| YPR188C | 86.6 | 9.1 | 6 |
| YMR205C | 4.1 | 0.4 | 96 |
| YDL171C | 1 | 0.1 | 61 |
| YKL007W | 6.6 | 0.4 | -202 |
| YJR133W | 37.4 | 4.2 | 68 |
| YDR454C | 8 | 1 | -87 |
| YIL014W | 3.2 | 0.3 | 10 |
| YGL092W | 0.5 | 0.1 | 28 |
| YDR303C | 1.5 | 0.2 | 25 |
| YPL059W | 268 | 18.2 | 94 |
| YER157W | 2.2 | 0.2 | 152 |
| YGL233W | 1.2 | 0.2 | 43 |
| YDR461W | 574.2 | 81.2 | 93 |
| YGR292W | 2.9 | 0.4 | 35 |
| YJL192C | 14.3 | 0.9 | 348 |
| YOR273C | 6.4 | 0.8 | -203 |
| YOR353C | 0.8 | 0.2 | 54 |
| YMR129W | 1.6 | 0.1 | 46 |
| YGL050W | 7.7 | 0.9 | 253 |
| YMR010W | 16.2 | 3.8 | -635 |
| YBR131W | 1 | 0.2 | 30 |
| YHR168W | 4.4 | 0.5 | 73 |
| YKL120W | 10.9 | 0.5 | 43 |
| YPR111W | 1.7 | 0.4 | 16 |
| YMR074C | 1.5 | 0.3 | -116 |
| YOR168W | 2 | 0.2 | 25 |
| YKR008W | 2.8 | 0.3 | 55 |
| YOR360C | 3.9 | 0.4 | 45 |
| YBL064C | 33.9 | 4.6 | 117 |
| YDR184C | 8.8 | 2.1 | 2 |
| YKL104C | 5.3 | 0.5 | 31 |
| YDR301W | 1 | 0.1 | -676 |
| YFL027C | 12 | 0.7 | 55 |
| YGR046W | 5.5 | 0.5 | 35 |
| YIL009W | 1.8 | 0.2 | 58 |
| YLR237W | 20.4 | 1.5 | 33 |
| YOR179C | 5.4 | 0.8 | 287 |
| YOR042W | 4.3 | 1.5 | 3 |
| YDR170C | 0.3 | 0.1 | 65 |
| YJL137C | 19.6 | 3 | 6 |
| YLR141W | 5.3 | 1 | 11 |
| YOL097C | 28.3 | 4.8 | 153 |
| YJL002C | 3 | 0.3 | 85 |
| YPL267W | 13.9 | 3.3 | 5 |
| YJL203W | 34.2 | 5.3 | -172 |
| YFL041W | 3.9 | 0.4 | 9 |
| YBR118W | 7.6 | 0.9 | 30 |
| YOL105C | 3.3 | 0.4 | 41 |
| YBR155W | 2.4 | 0.9 | 33 |
| YBR281C | 1.7 | 0.2 | 56 |
| YBR198C | 0.6 | 0.2 | 21 |
| YMR146C | 7.4 | 0.5 | 1250 |
| YDR247W | 2.7 | 0.3 | 28 |
| YJL124C | 10.8 | 5.4 | 76 |
| YDL002C | 5.7 | 1 | 54 |
| YJL207C | 2.1 | 0.2 | 25 |
| YIR021W | 11.3 | 0.9 | 48 |
| YPL206C | 41.1 | 3.9 | 18 |
| YOR319W | 5.5 | 0.5 | 87 |
| YNL168C | 2.4 | 0.5 | 444 |
| YHL025W | 5.9 | 0.9 | 29 |
| YER114C | 0.8 | 0.2 | 22 |
| YIL010W | 27 | 4.4 | 48 |
| YDL157C | 81.8 | 14.9 | 37 |
| YOR326W | 1.5 | 0.2 | 168 |
| YCR011C | 1.4 | 0.2 | -482 |
| YOL011W | 3.1 | 0.3 | 15 |
| YDR050C | 6.3 | 0.5 | 96 |
| YNL040W | 26.9 | 2 | 15 |
| YHR195W | 1.9 | 0.8 | 5 |
| YGL240W | 4.6 | 0.4 | 17 |
| YHR181W | 38.6 | 3.5 | 30 |
| YPL107W | 74.1 | 10.3 | 74 |
| YDL219W | 13.6 | 1.2 | 466 |
| YLR332W | 3.9 | 0.7 | 53 |
| YPL243W | 4.2 | 0.3 | 37 |
| YPL040C | 2.9 | 0.2 | -194 |
| YDR161W | 5.3 | 0.5 | 107 |
| YDR025W | 53.3 | 9.3 | 26 |
| YJR132W | 10 | 0.8 | 61 |
| YEL040W | 2.9 | 0.4 | 22 |
| YGL065C | 3.5 | 0.3 | 20 |
| YPL196W | 2.4 | 0.4 | 411 |
| YOR265W |  |  | 30 |
| YHR009C | 10.3 | 1.5 | 82 |
| YPL176C | 1.5 | 0.2 | 3 |
| YGR128C | 2 | 0.2 | 80 |
| YGL009C | 3 | 0.2 | 29 |
| YHR107C | 4.9 | 0.5 | 121 |
| YGR147C | 3.3 | 1 | 114 |
| YPR141C | 1.6 | 0.5 | 27 |
| YHR215W | 18.1 | 1.3 | 6 |
| YPL212C | 1.5 | 0.5 | 151 |
| YPR107C | 2.4 | 0.5 | 146 |
| YNL137C | 2.4 | 0.5 | -130 |
| YDR233C | 15.9 | 6.8 | -142 |
| YJL099W | 3 | 0.3 | 18 |
| YGL054C | 36.7 | 1.1 | 40 |
| YKR043C | 7.5 | 1.1 | -131 |
| YGR116W | 0.8 | 0.2 | 36 |
| YNL056W | 135.7 | 10 | -137 |
| YGR234W | 10.4 | 0.9 | 83 |
| YLR116W | 2.4 | 0.6 | 39 |
| YLR188W | 8.8 | 0.7 | 22 |
| YOR215C | 70.5 | 4.7 | 100 |
| YLR182W | 0.7 | 0.2 | 17 |
| YER022W | 2 | 0.6 | 39 |
| YKL056C | 112.4 | 12 | 166 |
| YML003W | 11.5 | 0.7 | 27 |
| YKL183W | 3.7 | 0.5 | 114 |
| YOR021C | 192.7 | 15.7 | 129 |
| YKR098C | 2.7 | 0.2 | 5 |
| YKL144C | 49.2 | 6.9 | 142 |
| YDR014W | 5.8 | 0.8 | 15 |
| YBR279W | 10.5 | 1.8 | 117 |
| YKL130C | 48.2 | 3.1 | 2258 |
| YJL001W | 2.7 | 1.1 | 36 |
| YNL329C | 7.1 | 0.5 | 44 |
| YLR377C | 1.7 | 0.5 | 81 |
| YCR026C | 2.4 | 0.3 | 11 |
| YKL027W | 5.8 | 0.5 | 59 |
| YMR052W | 15.7 | 3.2 | 7 |
| YDR539W | 58.4 | 5.3 | 42 |
| YNR036C | 7.5 | 1 | 13 |
| YCR037C | 12.9 | 1.4 | 10 |
| YDR331W | 27.5 | 1.8 | 21 |
| YER019C-A | 22.5 | 1.2 | 20 |
| YKL185W | 2.6 | 0.5 | 2 |
| YDR080W | 1.3 | 0.3 | 124 |
| YKL096W | 12.2 | 1.1 | 25 |
| YIL123W | 5.4 | 0.5 | 23 |
| YLR249W | 2.4 | 0.2 | 191 |
| YKL048C | 1.4 | 0.3 | 42 |
| YMR270C | 3.7 | 0.9 | 9 |
| YGL175C | 4.5 | 0.8 | 16 |
| YNL298W | 0.5 | 0.2 | 105 |
| YJL118W | 200.4 | 8.8 | 30 |
| YLR135W | 1.4 | 0.4 | 31 |
| YDR229W | 0.7 | 0.2 | 36 |
| YKR085C | 5 | 1.1 | 82 |
| YKR044W | 2.5 | 0.3 | 68 |
| YBR172C | 1.5 | 0.3 | 19 |
| YPR103W | 44.4 | 4.8 | -182 |
| YGR080W | 10.8 | 0.8 | 150 |
| YMR288W | 3.7 | 0.4 | 27 |
| YFL008W | 2.2 | 0.2 | 25 |
| YBR068C | 12.5 | 0.8 | 18 |
| YGR060W | 34.2 | 4.3 | -646 |
| YGL107C | 2.3 | 0.3 | 55 |
| YLL038C | 9.7 | 0.9 | 55 |
| YDR099W | 70.4 | 5.7 | -300 |
| YJL201W | 10.5 | 1.3 | 78 |
| YNL246W | 97.8 | 16 | -65 |
| YOR141C | 1.6 | 0.4 | 199 |
| YIL129C | 1.1 | 0.1 | 299 |
| YPL047W | 2.7 | 0.4 | 21 |
| YOR373W | 0.8 | 0.1 | 32 |
| YOR083W | 1.9 | 0.5 | 71 |
| YGL164C | 6.3 | 0.7 | 59 |
| YIL127C | 1.8 | 0.4 | 60 |
| YNL200C | 12.5 | 1.1 | 141 |
| YGR088W | 2.8 | 0.4 | 33 |
| YDR444W | 1.8 | 0.4 | 4 |
| YNL005C | 11.4 | 0.8 | 26 |
| YKL176C | 2.1 | 0.3 | 6 |
| YNL148C | 7.2 | 0.9 | 2 |
| YJR121W | 69.1 | 6.5 | 6627 |
| YEL019C | 21.5 | 2.9 | 121 |
| YLR323C | 14.1 | 3 | 45 |
| YDR183W | 5.5 | 0.5 | 20 |
| YKL051W | 24.4 | 2.8 | 57 |
| YML060W | 9.2 | 0.8 | 36 |
| YDR224C | 174.3 | 9 | -1052 |
| YNL256W | 3.9 | 0.3 | 13 |
| YLR193C | 77.3 | 8.1 | 6 |
| YLR175W | 28.6 | 4 | 30 |
| YJR008W | 3.6 | 0.5 | 19 |
| YBL010C | 6.4 | 0.8 | 111 |
| YGR195W | 13.7 | 0.9 | -308 |
| YBL040C | 229.7 | 10.9 | 96 |
| YER014W | 14.6 | 1.2 | 151 |
| YBR092C | 9.2 | 0.7 | 9 |
| YMR233W | 20 | 4.7 | 106 |
| YER031C | 0.8 | 0.3 | 77 |
| YOR035C | 3.7 | 0.4 | 38 |
| YOR189W | 18.7 | 5.4 | -230 |
| YDR021W | 55 | 3.9 | 46 |
| YPR040W | 3 | 0.4 | 129 |
| YOL042W | 26.2 | 2.5 | 47 |
| YER038C | 7 | 1.2 | 172 |
| YJL092W | 1.2 | 0.2 | 42 |
| YML046W | 13.5 | 1 | 62 |
| YPR171W | 2.2 | 0.6 | 65 |
| YGR112W | 2.6 | 0.5 | 29 |
| YML070W | 6.7 | 0.4 | 42 |
| YDR036C | 7.6 | 0.6 | 67 |
| YNL155W | 12.9 | 1.9 | 78 |
| YOR219C | 1 | 0.1 | 15 |
| YKL155C | 3 | 0.4 | 47 |
| YFR049W | 9.2 | 1 | 1628 |
| YER040W | 0.7 | 0.2 | 3 |
| YDL045C | 8.3 | 0.5 | 1197 |
| YNL308C | 3.1 | 0.5 | 31 |
| YOL056W | 4 | 0.5 | -8292 |
| YNR034W | 1.4 | 0.5 | 37 |
| YHR163W | 94.4 | 7.1 | 396 |
| YPL111W | 10.1 | 0.9 | 69 |
| YJR060W | 1 | 0.5 | 38 |
| YDR384C | 31 | 3 | 36 |
| YBR048W | 53.3 | 9.3 | 19 |
| YJL050W | 1.4 | 0.3 | 47 |
| YKL137W | 14 | 1.1 | 5 |
| YKR007W | 13.7 | 2.6 | 88 |
| YKL193C | 10.2 | 1.7 | 89 |
| YGR052W | 11.4 | 0.8 | 35 |
| YBR121C | 4.6 | 0.5 | 44 |
| YNL295W | 14 | 1.5 | 15 |
| YNL213C | 12.8 | 2.3 | 62 |
| YPL184C | 1.8 | 0.3 | 65 |
| YDR296W | 5.9 | 0.9 | 1417 |
| YER082C | 0.8 | 0.2 | 54 |
| YBR185C | 7.1 | 0.8 | 187 |
| YBL023C | 1.1 | 0.2 | 124 |
| YBR112C | 0.8 | 0.3 | 13 |
| YAL056W | 2 | 0.2 | 8 |
| YCR035C | 14.5 | 2.4 | 98 |
| YHL014C | 13.2 | 0.8 | -305 |
| YKL191W | 20.6 | 2.3 | 448 |
| YGL221C | 10.6 | 0.9 | 161 |
| YBR195C | 3.3 | 0.3 | 31 |
| YFL001W | 5.4 | 0.5 | 78 |
| YKL159C | 2.3 | 0.5 | 45 |
| YPL120W | 1.4 | 0.3 | 24 |
| YPR046W | 19.8 | 3.5 | 104 |
| YNL107W | 1.2 | 0.5 | 92 |
| YIL128W | 5.2 | 0.3 | 24 |
| YNL119W | 8 | 0.6 | 51 |
| YDR169C | 0.4 | 0.2 | 39 |
| YKL210W | 1.2 | 0.2 | 114 |
| YGL163C | 1.1 | 0.3 | 14 |
| YKL127W | 2.4 | 0.2 | 23 |
| YHR173C | 3 | 0.5 | 4 |
| YHR059W | 44.1 | 5.2 | 11 |
| YIL101C | 3.3 | 0.5 | 19 |
| YMR235C | 2.8 | 0.3 | 34 |
| YLR086W | 2.7 | 0.3 | 36 |
| YBR022W | 8.7 | 0.5 | 65 |
| YIL007C | 7.3 | 0.8 | 674 |
| YKL058W | 4.3 | 1 | 170 |
| YGR165W | 17.7 | 2.4 | 48 |
| YGL226W | 6.3 | 1.1 | 23 |
| YIL159W | 0.7 | 0.2 | 20 |
| YIL034C | 11.5 | 0.9 | 68 |
| YHR201C | 29.5 | 2.8 | 139 |
| YMR159C | 23.5 | 3.2 | 56 |
| YMR226C | 13.5 | 0.9 | 47 |
| YDR092W | 84.3 | 10 | -350 |
| YFR025C | 12.5 | 0.8 | 119 |
| YIL094C | 68.2 | 4.5 | 18 |
| YHR119W | 0.6 | 0.2 | 1814 |
| YDR450W | 89.7 | 8.8 | 63 |
| YKL170W | 49.6 | 4.9 | -122 |
| YKL117W | 10.3 | 1.1 | 280 |
| YML014W | 9.2 | 1 | 47 |
| YPL115C | 2.1 | 0.4 | 60 |
| YCR088W | 4.4 | 1.4 | 18 |
| YNL183C | 0.7 | 0.2 | 7 |
| YOR039W | 202.4 | 22.9 | -1054 |
| YJR143C | 3 | 0.3 | 19 |
| YOR307C | 25.6 | 2.3 | 90 |
| YLR383W | 1.9 | 0.3 | 57 |
| YER051W | 1.8 | 0.2 | 38 |
| YBR081C | 0.6 | 0.1 | 193 |
| YDR462W | 52.3 | 9.2 | 8 |
| YGR185C | 9.1 | 0.8 | 121 |
| YDR016C | 12.3 | 0.6 | 80 |
| YLR025W | 11.7 | 2.3 | 324 |
| YDR020C | 26.8 | 2.6 | 27 |
| YHR067W | 66.4 | 5.6 | -187 |
| YBR231C | 23.2 | 3.8 | 22 |
| YLR226W | 9.3 | 0.8 | 115 |
| YGL095C | 7 | 0.6 | 68 |
| YMR241W | 11.9 | 0.9 | 21 |
| YKL112W | 0.4 | 0.2 | 2 |
| YKL166C | 1.8 | 0.3 | 24 |
| YNL258C | 1.9 | 0.2 | 29 |
| YMR048W | 3.2 | 0.9 | 2 |
| YOL052C-A | 11.6 | 1 | -475 |
| YBR137W | 14.5 | 1.1 | 16 |
| YCR079W | 4.3 | 0.4 | 67 |
| YDR101C | 2.6 | 0.5 | 96 |
| YCR008W | 2.2 | 0.4 | 12 |
| YNL118C | 0.8 | 0.2 | 36 |
| YDR379C-A | 143.8 | 14 | 11 |
| YLR271W | 8.5 | 2 | 19 |
| YBR200W | 1.2 | 0.2 | 19 |
| YAL011W | 6.8 | 1.3 | 56 |
| YCR030C | 1.4 | 0.2 | -2326 |
| YPL011C | 7 | 0.8 | 40 |
| YNL268W | 11.2 | 0.7 | 21 |
| YLR420W | 34.4 | 2.9 | 94 |
| YKR067W | 4.2 | 0.4 | 24 |
| YOL016C | 4.7 | 0.8 | -70 |
| YNR009W | 11.1 | 2.8 | 19 |
| YMR137C | 3.3 | 0.4 | 16 |
| YOR223W | 0.8 | 0.3 | 22 |
| YJL213W | 6.1 | 0.5 | 26 |
| YPR070W | 1.4 | 0.2 | 25 |
| YOR154W | 2.5 | 0.4 | 21 |
| YNR014W | 17.4 | 4.2 | 109 |
| YLR194C | 3.9 | 0.5 | 2 |
| YMR115W | 12.5 | 2.2 | 156 |
| YHR200W | 3.4 | 0.5 | 46 |
| YOL135C | 3.3 | 0.9 | 37 |
| YDR118W | 8.3 | 0.6 | 100 |
| YKR017C | 9.5 | 0.6 | 23 |
| YGL137W | 1.5 | 0.1 | 53 |
| YJL048C | 0.8 | 0.2 | 38 |
| YLR107W | 2.6 | 0.3 | 80 |
| YGR026W | 99 | 5.2 | 63 |
| YDR515W | 0.5 | 0.2 | 24 |
| YNL133C | 4 | 0.9 | 19 |
| YGR206W | 11.1 | 5.7 | 16 |
| YNL310C | 5.3 | 1 | 101 |
| YOR161C | 3 | 0.3 | 43 |
| YBR015C | 4.6 | 0.4 | 15 |
| YMR179W | 1.9 | 0.3 | 45 |
| YAL059W | 6.1 | 0.9 | 36 |
| YGR007W | 3.9 | 0.4 | 173 |
| YER018C | 38.5 | 4.3 | 167 |
| YDL138W | 1.4 | 0.2 | 3 |
| YNR054C | 1.9 | 0.5 | 94 |
| YCR087C-A | 4.3 | 0.9 | 117 |
| YOR336W | 1.5 | 0.1 | 7 |
| YNL325C | 3 | 0.4 | 18 |
| YLR361C | 6.3 | 0.7 | 10 |
| YEL005C | 9 | 0.7 | 75 |
| YMR021C | 1.3 | 0.3 | 93 |
| YGR012W | 7.4 | 0.8 | -373 |
| YJR032W | 5.3 | 0.5 | 41 |
| YPR002W | 7.4 | 0.8 | 34 |
| YNL020C | 3.9 | 0.5 | 37 |
| YMR071C | 21.2 | 1.1 | 44 |
| YPL214C | 6.4 | 0.5 | 77 |
| YPL247C | 0.8 | 0.2 | 60 |
| YGR253C | 63.1 | 6.8 | -198 |
| YMR236W | 1.3 | 1 | 93 |
| YDL238C | 3.4 | 0.3 | 172 |
| YER118C | 2.1 | 0.4 | 95 |
| YNR016C | 0.6 | 0.1 | 233 |
| YCR093W | 3 | 0.2 | 53 |
| YPR174C | 17.6 | 4.5 | 139 |
| YKL014C | 1.8 | 0.1 | 45 |
| YDR376W | 4.5 | 0.3 | 78 |
| YJR119C | 3.3 | 0.3 | 36 |
| YJL062W-A | 264.4 | 20.3 | 120 |
| YPR180W | 17.3 | 1 | 85 |
| YPR072W | 4.7 | 0.7 | 26 |
| YPR071W | 1.2 | 0.5 | 165 |
| YOR385W | 26 | 5.3 | 55 |
| YDL190C | 5.9 | 0.3 | -781 |
| YCR075C | 95 | 3.7 | 7 |
| YCR066W | 2.2 | 0.4 | 22 |
| YGL244W | 8.2 | 1.6 | 23 |
| YER100W | 3.4 | 0.5 | 26 |
| YLR275W | 71 | 5.2 | 44 |
| YKR059W | 85.3 | 5.4 | -1505 |
| YOR007C | 14.6 | 2.6 | 1551 |
| YLR035C | 3.6 | 0.4 | 21 |
| YDL139C | 2.8 | 0.5 | 28 |
| YOL098C | 3.3 | 0.3 | 24 |
| YIL047C | 6.1 | 0.4 | 22 |
| YFL034C-B | 5.1 | 0.8 | 18 |
| YMR003W | 43.2 | 5.7 | 21 |
| YDR167W | 7.6 | 0.9 | -67 |
| YGR162W | 0.7 | 0.2 | 42 |
| YJR078W | 11.7 | 1.7 | 20 |
| YBR158W | 2.6 | 0.5 | 17 |
| YDR179W-A | 11.9 | 1.1 | 24 |
| YPL037C | 37.3 | 7.1 | -103 |
| YLR145W | 19.6 | 0.9 | -327 |
| YDR333C | 1.1 | 0.3 | 19 |
| YOR132W | 1 | 0.3 | 20 |
| YER151C | 0.5 | 0.1 | 45 |
| YPL013C | 43.2 | 4.1 | 59 |
| YPL110C | 0.7 | 0.1 | -202 |
| YMR173W | 1.1 | 0.4 | 63 |
| YDR451C | 8.1 | 2 | 3 |
| YPL071C | 1.6 | 0.5 | 61 |
| YBL002W | 174.3 | 9 | -1503 |
| YJL183W | 2.3 | 0.5 | 8 |
| YNL024C | 16.6 | 1.1 | 13 |
| YLR462W | 53.6 | 5.4 | 2 |
| YML037C | 3.3 | 0.5 | 2 |
| YHR141C | 438.8 | 253 | 2222 |
| YER027C | 1.2 | 0.4 | 34 |
| YHL020C | 4.1 | 0.8 | 76 |
| YGL256W | 9.7 | 0.8 | -1042 |
| YPL084W | 3.5 | 0.4 | 27 |
| YNL227C | 7.4 | 1 | 35 |
| YDL119C | 33.5 | 3.2 | 429 |
| YJR056C | 4.2 | 0.7 | 2 |
| YMR309C | 3.2 | 0.6 | -1457 |
| YJL071W | 7.1 | 0.6 | 8 |
| YNL234W | 4.9 | 0.7 | 25 |
| YLR103C | 1.3 | 0.4 | 78 |
| YDL143W | 4.9 | 0.7 | 125 |
| YBR046C | 13.1 | 0.9 | 2 |
| YDR071C | 60.5 | 4.5 | 47 |
| YKL053C-A | 4.9 | 1.1 | 40 |
| YNL136W | 0.6 | 0.2 | 11 |
| YER143W | 10.9 | 0.8 | 16 |
| YBR103W | 3.4 | 0.4 | 39 |
| YOR357C | 5.1 | 1 | 29 |
| YDL008W | 12.3 | 1 | 11 |
| YKR094C | 153.5 | 16.4 | -77 |
| YIL084C | 0.7 | 0.3 | 27 |
| YDL161W | 1.9 | 0.6 | 39 |
| YGR043C | 28.9 | 4.5 | 61 |
| YJL089W | 3.6 | 0.3 | 20 |
| YOR321W | 4 | 0.4 | 9 |
| YJL127C | 1.6 | 0.3 | 22 |
| YPL014W | 2.7 | 0.5 | 11 |
| YPL015C | 2.7 | 0.9 | 33 |
| YIL056W | 0.6 | 0.2 | 26 |
| YOR274W | 4.9 | 0.5 | 12 |
| YGL161C | 2.6 | 0.4 | 9 |
| YIR006C | 0.3 | 0.1 | 3 |
| YGR133W | 14.9 | 3.4 | 82 |
| YGR175C | 9.4 | 0.8 | 40 |
| YMR116C | 9.9 | 0.9 | 157 |
| YPL125W | 9.2 | 0.5 | 214 |
| YMR080C | 2.4 | 0.2 | 18 |
| YJL102W | 2.3 | 0.3 | 40 |
| YGR229C | 1 | 0.2 | 39 |
| YDR400W | 10.1 | 0.8 | 106 |
| YBR177C | 2 | 0.3 | -779 |
| YCR071C | 11.3 | 0.9 | 190 |
| YOL107W | 46.2 | 3 | 32 |
| YBR067C | 51.3 | 4.9 | 233 |
| YDL074C | 2.2 | 0.5 | 61 |
| YNL241C | 11.8 | 0.8 | -117 |
| YJL039C | 2 | 0.2 | 194 |
| YNL121C | 2 | 0.3 | 29 |
| YDR538W | 362.2 | 20.3 | 27 |
| YDR164C | 3.6 | 0.6 | 2 |
| YDL170W | 22.3 | 1.4 | 22 |
| YCR020W-B | 18.6 | 5.7 | 30 |
| YJR084W | 35.2 | 2.5 | 25 |
| YHR147C | 27.5 | 3.1 | 73 |
| YLR108C | 3.2 | 0.3 | 18 |
| YNL032W | 7.6 | 0.8 | 516 |
| YKL043W | 1.9 | 0.3 | 14 |
| YPR020W | 343 | 16.4 | 25 |
| YHR138C | 97.7 | 9.8 | 87 |
| YOL027C | 9.3 | 0.8 | 43 |
| YDL220C | 2.3 | 0.2 | 42 |
| YBR189W | 110.2 | 10 | 29 |
| YBR057C | 1.4 | 0.3 | 40 |
| YIL103W | 3.9 | 0.8 | 38 |
| YGR123C | 1.8 | 0.3 | 125 |
| YGR210C | 12.6 | 0.9 | 209 |
| YGR041W | 0.8 | 0.2 | 40 |
| YKR080W | 4 | 0.3 | 197 |
| YDR035W | 3.1 | 0.3 | 61 |
| YML092C | 4.7 | 0.4 | 7987 |
| YCR073W-A | 37.4 | 5.7 | 45 |
| YKL212W | 4 | 0.3 | 57 |
| YPL008W | 2.9 | 0.5 | 67 |
| YPR183W | 50.8 | 5 | 3 |
| YDR395W | 12 | 0.6 | 196 |
| YER147C | 48.7 | 2.5 | 136 |
| YCR043C | 189.1 | 18.8 | 27 |
| YER161C | 1.2 | 0.4 | 26 |
| YPR003C | 3.3 | 0.8 | 35 |
| YFL060C | 10.7 | 1 | 73 |
| YLR344W | 9.2 | 1.2 | 358 |
| YER164W | 0.6 | 0.2 | 19 |
| YOL149W | 1 | 0.3 | 23 |
| YGL141W | 4.2 | 0.3 | 28 |
| YIR007W | 4.3 | 0.4 | 270 |
| YLR196W | 6.5 | 0.7 | 46 |
| YOR377W | 5.2 | 0.7 | 103 |
| YDR437W | 183.6 | 14.7 | -109 |
| YNL330C | 19.1 | 2.6 | 1168 |
| YMR044W | 6.8 | 0.8 | 16 |
| YKR037C | 5.1 | 0.5 | 172 |
| YDR087C | 16.2 | 2.5 | 51 |
| YMR120C | 5.4 | 0.5 | 39 |
| YNL085W | 3.3 | 0.3 | 28 |
| YPL057C | 6.6 | 0.8 | 33 |
| YNL286W | 2.6 | 0.8 | 3 |
| YKL052C | 1.7 | 0.8 | 3 |
| YJR005W | 7.6 | 1 | 34 |
| YFL017C | 98.4 | 5.8 | 25 |
| YDR039C | 2 | 0.3 | 288 |
| YFL062W | 8.3 | 0.9 | 115 |
| YLR148W | 4.5 | 0.3 | 52 |
| YLR203C | 34.3 | 4.4 | 87 |
| YGL035C | 1.6 | 0.5 | 35 |
| YBR072W | 6 | 0.8 | -113 |
| YDR377W | 83.9 | 9.2 | 50 |
| YDR240C | 6.9 | 1.6 | 268 |
| YDL089W | 10.1 | 1.6 | 27 |
| YBR259W | 8.1 | 0.6 | 15 |
| YOL136C | 2.3 | 0.4 | 52 |
| YDR185C | 45.2 | 4.2 | 25 |
| YOL032W | 2.7 | 0.5 | -377 |
| YFL029C | 23 | 2.3 | 141 |
| YNL116W | 1.6 | 0.4 | 23 |
| YJR117W | 40.2 | 2.1 | 24 |
| YKL017C | 2.9 | 0.3 | 35 |
| YLR094C | 4.3 | 0.7 | 2 |
| YNR024W | 1 | 0.5 | 2 |
| YHL048W | 7.8 | 0.8 | 19 |
| YDL010W | 4.5 | 0.9 | 2 |
| YDL209C | 4.9 | 0.8 | 75 |
| YGR118W | 10.1 | 0.9 | 265 |
| YDR363W-A | 6.3 | 1.1 | 37 |
| YDR276C | 10533 | 318 | 77 |
| YOL057W | 6.6 | 0.6 | 56 |
| YDR487C | 58.4 | 5.9 | 20 |
| YDL064W | 40.5 | 7.9 | 86 |
| YHR094C | 4.2 | 0.7 | -221 |
| YHR042W | 4.3 | 0.3 | 61 |
| YDR109C | 2.4 | 0.3 | 3 |
| YIL156W | 2.6 | 0.2 | 48 |
| YNL229C | 0.7 | 0.3 | 39 |
| YLR154C | 5.3 | 1 | 18 |
| YNL220W | 4.4 | 0.4 | 57 |
| YMR174C | 41.5 | 19.7 | -178 |
| YCL056C | 15.1 | 1.1 | 37 |
| YIR033W | 0.7 | 0.1 | 31 |
| YPR128C | 8.1 | 0.5 | 39 |
| YGL119W | 5.2 | 0.5 | 25 |
| YIL155C | 5.6 | 0.4 | 51 |
| YNL153C | 58.2 | 11.1 | -2214 |
| YNR033W | 1.5 | 0.1 | 28 |
| YOR014W | 1.6 | 0.2 | 18 |
| YPL069C | 26.5 | 1.6 | 95 |
| YHR025W | 12 | 0.9 | 73 |
| YDR465C | 2.5 | 0.4 | 69 |
| YDR361C | 1.5 | 0.5 | 169 |
| YIL107C | 2.3 | 0.5 | 25 |
| YIR004W | 2.3 | 0.9 | 167 |
| YLR398C | 0.6 | 0.1 | 40 |
| YDL222C | 2.5 | 0.5 | 74 |
| YPL079W | 9.6 | 1.1 | 84 |
| YCR067C | 2.9 | 0.3 | 62 |
| YPR133C | 1.7 | 0.7 | 86 |
| YLR209C | 3.3 | 0.4 | 31 |
| YOL069W | 22.4 | 1.7 | 114 |
| YGL133W | 1 | 0.2 | 116 |
| YER081W | 3 | 0.4 | 192 |
| YLR115W | 1.6 | 0.3 | 12 |
| YLR333C |  |  | 427 |
| YKL206C | 12.7 | 0.8 | -647 |
| YOR147W | 5.5 | 0.4 | 32 |
| YDL095W | 14.4 | 1.4 | 171 |
| YBR218C | 1.7 | 0.2 | 40 |
| YER120W | 7.8 | 0.9 | 553 |
| YDR330W | 4 | 0.6 | 41 |
| YLR144C | 3.1 | 0.3 | 22 |
| YBR302C | 7.9 | 0.8 | 8 |
| YMR152W | 8.5 | 0.7 | 27 |
| YJL209W | 5.5 | 0.4 | 43 |
| YNR052C | 1.4 | 0.3 | 48 |
| YGR260W | 23.3 | 2.7 | 31 |
| YDR121W | 79.4 | 6.7 | 137 |
| YKR029C | 1.1 | 0.3 | 3 |
| YOL129W | 138.7 | 5.7 | -2417 |
| YOR324C | 1.8 | 0.4 | 9 |
| YBL051C | 0.4 | 0.1 | 20 |
| YKL016C | 67.8 | 8.1 | 16 |
| YOL145C | 3.1 | 0.4 | 24 |
| YGR093W | 6 | 0.6 | 70 |
| YJR130C | 2.7 | 0.4 | 45 |
| YPL195W | 3.9 | 0.5 | 331 |
| YCL050C | 4.8 | 0.5 | 122 |
| YPR032W | 1.5 | 0.1 | 46 |
| YDR211W | 6.9 | 0.9 | -302 |
| YLR199C | 8.2 | 1 | 95 |
| YOR111W | 18.8 | 4.5 | 42 |
| YIL027C | 10.9 | 0.9 | 35 |
| YER062C | 6.6 | 0.9 | -948 |
| YGL004C | 4.7 | 0.4 | 53 |
| YPL239W | 5.6 | 0.5 | 25 |
| YBR248C | 8.8 | 0.7 | 56 |
| YDR135C | 1.8 | 0.2 | 56 |
| YML008C | 19.7 | 2.5 | 135 |
| YIR036C | 6.6 | 0.5 | 54 |
| YLR300W | 3.4 | 0.3 | 15 |
| YOL012C | 384.2 | 17.6 | 261 |
| YHL036W | 27.8 | 3.6 | 55 |
| YPR172W | 30.7 | 3 | 142 |
| YIL149C | 1.4 | 0.2 | 20 |
| YDR342C | 7.2 | 0.9 | 81 |
| YER073W | 3.5 | 0.3 | 64 |
| YKR086W | 1 | 0.3 | 4 |
| YCR081W | 1.2 | 0.3 | 41 |
| YPL118W | 3.2 | 0.4 | 16 |
| YLR072W | 1.5 | 0.3 | 10 |
| YER086W | 21 | 1.6 | 44 |
| YNL122C | 10.4 | 1 | -2373 |
| YIL083C | 2.9 | 0.5 | 75 |
| YLR408C | 19.8 | 4.1 | 61 |
| YEL039C | 90 | 12.2 | 236 |
| YML024W | 8.1 | 1.1 | -1238 |
| YLR313C | 2.3 | 0.3 | 31 |
| YGR221C | 1.8 | 0.4 | 140 |
| YGR001C | 12.5 | 0.9 | 51 |
| YLR413W | 14.6 | 1.4 | 72 |
| YPR017C | 75.9 | 6.5 | 154 |
| YNL173C | 1.7 | 0.5 | 40 |
| YGL166W | 1.9 | 0.3 | 23 |
| YBR282W | 9.1 | 1 | 132 |
| YMR302C | 3.3 | 0.4 | 46 |
| YJR070C | 28.5 | 3.3 | 63 |
| YJL004C | 22.9 | 5.3 | 57 |
| YPL091W | 10.2 | 0.7 | 35 |
| YDL087C | 19.5 | 1 | -591 |
| YOR347C | 5.1 | 0.5 | 462 |
| YMR054W | 3.9 | 0.3 | 33 |
| YKL113C | 9.4 | 1 | -135 |
| YJR053W | 1.1 | 0.2 | 38 |
| YDR144C | 2.9 | 0.6 | 31 |
| YMR277W | 1.6 | 0.4 | 20 |
| YPL065W | 23.2 | 2.3 | 259 |
| YDL005C | 0.3 | 0.1 | 38 |
| YOL010W | 14.4 | 0.9 | 46 |
| YJL159W | 134.8 | 14.4 | 8 |
| YGR189C | 2.9 | 0.4 | 31 |
| YIR003W | 0.9 | 0.3 | 32 |
| YDL230W | 7.8 | 0.8 | 11 |
| YHR114W | 1.2 | 0.3 | 1187 |
| YIL157C | 36.3 | 3.9 | 120 |
| YGL079W | 4.5 | 0.8 | 39 |
| YGL203C | 1.6 | 0.2 | 51 |
| YLR380W | 3.4 | 0.3 | 66 |
| YBR214W | 2.3 | 0.4 | 54 |
| YDR009W | 3 | 0.3 | 18 |
| YJL178C | 2 | 0.5 | 44 |
| YOR027W | 5 | 0.6 | 89 |
| YGL059W | 6.6 | 0.5 | 57 |
| YHR090C | 1.5 | 0.5 | 32 |
| YDL156W | 5.1 | 0.5 | 113 |
| YGL201C | 0.9 | 0.2 | 21 |
| YNL130C | 29.5 | 1 | 39 |
| YPR186C | 3.8 | 0.4 | 99 |
| YER166W | 1.5 | 0.2 | -87 |
| YHR082C | 0.4 | 0.1 | 35 |
| YKL023W | 0.9 | 0.4 | 44 |
| YLR319C | 3.3 | 0.5 | 35 |
| YOR298C-A | 25.5 | 5.1 | 106 |
| YEL031W | 2.4 | 0.2 | -113 |
| YBR167C | 34.6 | 13.8 | 66 |
| YLR228C | 0.5 | 0.1 | 2 |
| YIL042C | 47.5 | 2.8 | 121 |
| YAR014C | 0.8 | 0.2 | 29 |
| YBR241C | 3.3 | 0.9 | 86 |
| YOR239W | 0.8 | 0.4 | -142 |
| YEL046C | 3.6 | 0.4 | 1367 |
| YGL025C | 0.7 | 0.2 | 21 |
| YBR278W | 22.4 | 3.5 | 1162 |
| YGR082W | 6.2 | 1.1 | -998 |
| YLR316C | 5.9 | 0.4 | 25 |
| YGL061C | 2.3 | 0.7 | 76 |
| YBR268W | 77.1 | 10.4 | -477 |
| YER084W | 163.9 | 9.3 | 17 |
| YLR403W | 0.4 | 0.1 | 15 |
| YOL078W | 0.5 | 0.1 | 11 |
| YOR370C | 3.5 | 0.5 | 58 |
| YJL082W | 2.8 | 0.3 | 103 |
| YGR248W | 28.6 | 3.1 | -72 |
| YMR229C | 1.9 | 0.2 | 15 |
| YBR288C | 7 | 0.7 | 79 |
| YDL120W | 5.6 | 0.5 | -179 |
| YDR022C | 8.9 | 2.5 | 165 |
| YLL012W | 3.6 | 0.4 | 32 |
| YDL188C | 8.4 | 1.6 | 61 |
| YDR436W | 0.6 | 0.2 | 43 |
| YIL070C | 16.1 | 2.5 | -1024 |
| YJR112W | 22.8 | 3.2 | 170 |
| YKL041W | 8.3 | 0.9 | -77 |
| YMR038C | 6 | 0.7 | -159 |
| YBL097W | 0.7 | 0.2 | 3 |
| YMR036C | 2.9 | 0.7 | 12 |
| YBR006W | 8.8 | 0.8 | 31 |
| YMR271C | 77.7 | 4.9 | 32 |
| YPL177C | 7.2 | 1.3 | 27 |
| YOR152C | 1.3 | 0.5 | 69 |
| YBR069C | 6.8 | 0.4 | 42 |
| YPR061C | 7.1 | 0.9 | 11 |
| YPR169W | 7.3 | 0.7 | 50 |
| YOR234C | 67.6 | 7.7 | -208 |
| YML071C | 3.2 | 0.6 | 31 |
| YBR119W | 18.1 | 2.6 | -18948 |
| YOR070C | 2.6 | 0.4 | 21 |
| YCL047C | 10.9 | 1 | 210 |
| YER053C-A | 509 | 31 | -231 |
| YPL183C | 2 | 0.2 | 21 |
| YOR047C | 3.1 | 0.5 | 8 |
| YDL069C | 31.8 | 2.4 | 120 |
| YLR384C | 2 | 0.2 | 165 |
| YBR053C | 4.9 | 0.5 | 20 |
| YFL049W | 1.8 | 0.3 | 3 |
| YMR293C | 42.7 | 2.9 | 85 |
| YOL103W | 5.5 | 0.4 | 71 |
| YOR040W | 24.5 | 3.1 | 252 |
| YGL143C | 4.5 | 0.9 | 123 |
| YPR098C | 751.3 | 25.2 | 65 |
| YDR066C | 101.3 | 5.6 | 113 |
| YBR130C | 1.5 | 0.5 | 70 |
| YLR206W | 5.7 | 1.7 | 18 |
| YGL112C | 22.2 | 2.2 | 23 |
| YDL006W | 3.5 | 0.3 | -247 |
| YGR065C | 6 | 0.4 | 42 |
| YDR332W | 4.1 | 0.4 | 46 |
| YHR143W-A | 859.2 | 128 | -679 |
| YEL071W | 5.7 | 0.4 | 56 |
| YHR086W | 1 | 0.2 | 62 |
| YDR380W | 1.6 | 0.2 | 30 |
| YBR058C | 1.8 | 0.2 | 39 |
| YJL153C | 3.4 | 0.3 | 38 |
| YMR222C | 10 | 0.5 | 7 |
| YKL119C | 68.8 | 4.8 | 30 |
| YDL226C | 1.5 | 0.5 | 109 |
| YOR025W | 14.6 | 2.3 | 3 |
| YLR404W | 6.1 | 0.5 | -271 |
| YJL177W | 6 | 1 | 21 |
| YLR240W | 5.4 | 0.4 | 22 |
| YHL032C | 5.4 | 0.5 | 78 |
| YOR323C | 27.4 | 2.2 | 106 |
| YJL176C | 0.5 | 0.2 | 72 |
| YLR440C | 4.2 | 0.4 | 35 |
| YNL175C | 1.7 | 0.4 | 78 |
| YDL027C | 3.5 | 0.3 | 22 |
| YNL299W | 5.5 | 0.6 | 35 |
| YHL038C | 4.4 | 0.4 | 51 |
| YBR235W | 1.3 | 0.2 | 31 |
| YLR201C | 58.5 | 5.4 | -376 |
| YIL021W | 6.8 | 0.8 | 29 |
| YOL102C | 10.2 | 1 | 112 |
| YOR251C | 45.6 | 4.6 | 296 |
| YOR142W | 164.4 | 20.5 | 1026 |
| YGR194C | 10.8 | 0.6 | 50 |
| YBR054W | 81.9 | 5.8 | 83 |
| YMR281W | 21.7 | 1.3 | 52 |
| YBR036C | 1.7 | 0.3 | 31 |
| YER061C | 2.4 | 0.3 | -299 |
| YBR263W | 27.9 | 2.3 | 43 |
| YPR060C | 37.4 | 3.2 | 3 |
| YLR223C | 0.3 | 0.1 | 62 |
| YGR202C | 1.2 | 0.4 | 26 |
| YML065W | 3.4 | 0.3 | 85 |
| YJL098W | 0.6 | 0.1 | 60 |
| YDR311W | 1.4 | 0.3 | 35 |
| YDR308C | 47.1 | 4.4 | -460 |
| YBL021C | 1.7 | 0.5 | 51 |
| YLR174W | 14.7 | 1 | 69 |
| YLR456W | 8.6 | 0.9 | -293 |
| YGL122C | 4.1 | 0.6 | 44 |
| YDR418W | 100.5 | 12.5 | 4849 |
| YAL007C | 42.9 | 2.7 | 49 |
| YMR023C | 3.3 | 0.3 | 32 |
| YPL076W | 113.4 | 7.6 | 70 |
| YPL259C | 5.1 | 0.4 | 37 |
| YGL144C | 1.9 | 0.3 | 3 |
| YCR094W | 4.2 | 0.5 | 47 |
| YOR120W | 31.3 | 3.5 | 45 |
| YKR099W | 0.4 | 0.1 | 2 |
| YBR037C | 2.7 | 0.4 | 74 |
| YOR022C | 1.3 | 0.3 | 48 |
| YLR038C | 16.3 | 5 | 7 |
| YPL105C | 1.6 | 0.3 | 6 |
| YGR285C | 17.5 | 2.1 | 107 |
| YER064C | 3.3 | 0.7 | 2 |
| YLL031C | 8.7 | 0.5 | -110 |
| YER035W | 2.4 | 1.2 | 52 |
| YPL006W | 4.4 | 0.2 | 84 |
| YOR349W | 12.2 | 0.8 | 16 |
| YPR073C | 30.4 | 3.7 | 44 |
| YGR049W | 33.9 | 4.7 | 29 |
| YDR003W | 1.1 | 0.3 | 78 |
| YNL162W | 438.8 | 253 | 224 |
| YDR001C | 2.6 | 0.4 | 30 |
| YOR247W | 7.2 | 0.9 | 56 |
| YJL100W | 4.3 | 0.6 | -1051 |
| YLR290C | 11.8 | 0.8 | 34 |
| YMR250W | 11.7 | 1.6 | 62 |
| YGL252C | 7.4 | 0.5 | 69 |
| YDL021W | 6.7 | 0.5 | 47 |
| YFL034W | 1.1 | 0.4 | 64 |
| YHR053C | 127.3 | 6.6 | 250 |
| YJL090C | 2.7 | 0.3 | 19 |
| YLR460C | 6.1 | 0.4 | 9 |
| YER052C | 5 | 0.5 | 78 |
| YDR103W | 2.6 | 0.4 | 5 |
| YGR218W | 6.3 | 0.4 | 51 |
| YMR310C | 55.9 | 4.6 | 18 |
| YHR029C | 9.2 | 0.9 | 138 |
| YBL027W | 6 | 1 | 48 |
| YKL151C | 21.7 | 2.9 | 54 |
| YPR139C | 4.1 | 0.4 | 169 |
| YMR113W | 3.3 | 0.3 | 54 |
| YML032C | 1.3 | 0.3 | 27 |
| YEL054C | 100.5 | 12.5 | 10 |
| YDR180W | 3 | 0.2 | 21 |
| YFR011C | 9.4 | 0.9 | 53 |
| YIL054W | 910.6 | 46.3 | 6 |
| YDR423C | 2.9 | 0.5 | 26 |
| YIL015W | 2.4 | 0.3 | 7 |
| YML054C | 4 | 0.3 | 54 |
| YML056C | 3.4 | 0.3 | 65 |
| YOL143C | 36.9 | 5.3 | 61 |
| YDR202C | 2.6 | 0.4 | 1058 |
| YPL129W | 26.1 | 5.2 | 251 |
| YER153C | 25.3 | 1.1 | 83 |
| YKL160W | 17.9 | 3 | 119 |
| YLR002C | 2.6 | 0.4 | 44 |
| YGR071C | 5.5 | 0.6 | 38 |
| YDR478W | 16.3 | 2.3 | 75 |
| YLR052W | 4 | 0.7 | 65 |
| YNL111C | 15.6 | 1.1 | 73 |
| YGR214W | 24 | 5.9 | -324 |
| YDR314C | 5.9 | 0.5 | 11 |
| YER169W | 2.1 | 0.4 | 55 |
| YCR042C | 0.9 | 0.1 | 136 |
| YBR052C | 14.2 | 1 | -551 |
| YHR027C | 1.7 | 0.2 | 49 |
| YDR141C | 2.4 | 0.2 | 23 |
| YNR040W | 8 | 0.8 | 44 |
| YHR187W | 2.5 | 0.9 | 66 |
| YMR189W | 6.4 | 0.5 | 69 |
| YGL067W | 8.2 | 0.6 | 20 |
| YJR062C | 5.2 | 0.7 | 55 |
| YLR109W | 15.2 | 1.1 | -106 |
| YBR199W | 5.3 | 0.5 | 12 |
| YCR092C | 1.8 | 0.2 | 21 |
| YOL071W | 33.2 | 4.5 | 59 |
| YGL135W | 53.1 | 4.3 | 69 |
| YHR011W | 3 | 0.3 | 61 |
| YGL014W | 0.8 | 0.2 | 22 |
| YBR233W | 4.1 | 0.4 | 36 |
| YJL112W | 1.5 | 0.3 | 25 |
| YOR123C | 1.5 | 0.6 | 49 |
| YKL214C | 4.6 | 0.8 | 247 |
| YGL159W | 5.6 | 0.4 | 62 |
| YLR044C | 6.8 | 0.4 | 29 |
| YER041W | 5.3 | 0.6 | 18 |
| YDR291W | 3.4 | 0.4 | 16 |
| YJR137C | 1.9 | 0.2 | 125 |
| YGR220C | 9.6 | 0.8 | 65 |
| YGL115W | 16.7 | 0.9 | -126 |
| YIL122W | 6.4 | 1.7 | 26 |
| YCR054C | 5.4 | 0.4 | 87 |
| YOL112W | 2.1 | 0.3 | 28 |
| YPR118W | 4.1 | 0.4 | -308 |
| YER128W | 17.3 | 3.9 | 117 |
| YNL156C | 11.9 | 0.9 | 47 |
| YOR296W | 2.3 | 0.3 | 14 |
| YAL054C | 9.7 | 0.7 | 29 |
| YFR030W | 11 | 0.8 | 53 |
| YMR135C | 1.6 | 0.2 | 156 |
| YLR118C | 1.9 | 0.4 | -150 |
| YGR291C | 1674.9 | 53.6 | 3 |
| YAL001C | 1.6 | 0.2 | 475 |
| YGR275W | 17.9 | 4.2 | -1111 |
| YNL062C | 12.1 | 2 | 69 |
| YNR004W | 67.4 | 5.8 | -71 |
| YOR061W | 55.1 | 4.9 | 54 |
| YGL114W | 7.7 | 0.7 | 17 |
| YHR161C | 3.6 | 0.5 | 33 |
| YNL267W | 1.2 | 0.2 | -156 |
| YER098W | 1.9 | 0.3 | 9 |
| YDR038C | 2 | 0.3 | 43 |
| YPR155C | 4.4 | 0.4 | 41 |
| YOR133W | 5.1 | 0.5 | 155 |
| YLR423C | 6.6 | 0.7 | 43 |
| YML058W-A | 65.5 | 17 | 3 |
| YNR053C | 4.5 | 0.9 | 381 |
| YGR042W | 11.3 | 2.1 | 14 |
| YOR221C | 5.5 | 0.4 | 322 |
| YDR514C | 5.3 | 0.7 | 35 |
| YPL168W | 3 | 0.4 | 107 |
| YKL148C | 1.5 | 0.2 | 62 |
| YDL181W | 350.8 | 58.9 | 44 |
| YKR074W | 13.6 | 3.1 | -182 |
| YPR045C | 2.2 | 0.3 | 58 |
| YOL019W | 1 | 0.2 | 73 |
| YDR258C | 6.3 | 0.4 | 57 |
| YGL155W | 1.9 | 0.4 | 7806 |
| YBL028C | 108.2 | 18.1 | 73 |
| YDR292C | 1.8 | 0.4 | 49 |
| YEL058W | 2.6 | 0.2 | 43 |
| YPL148C | 189.2 | 15 | -879 |
| YNR049C | 1.9 | 0.8 | 39 |
| YPR143W | 6.9 | 2.4 | 66 |
| YJL016W | 1.9 | 0.3 | 31 |
| YBR150C | 0.5 | 0.2 | 73 |
| YLR185W | 5.4 | 1.1 | 316 |
| YJL097W | 21.1 | 1 | -147 |
| YJL212C | 3.3 | 0.2 | 2 |
| YCR047C | 42.6 | 5 | 44 |
| YNL014W | 1.7 | 0.2 | 3 |
| YLR399C | 0.4 | 0.1 | 59 |
| YHR186C | 0.6 | 0.1 | 112 |
| YJR011C | 30.7 | 2.3 | 68 |
| YFL050C | 0.8 | 0.2 | 3 |
| YKL059C | 2.8 | 0.8 | 33 |
| YMR313C | 15.2 | 1.4 | 21 |
| YNL336W | 7.6 | 0.8 | 58 |
| YAL033W | 27.4 | 3.7 | 47 |
| YMR243C | 8.3 | 2 | 35 |
| YLR431C | 10.7 | 1.4 | 24 |
| YNL320W | 26.9 | 2.1 | 23 |
| YDR075W | 32.7 | 2.2 | 61 |
| YDR120C | 1.9 | 0.4 | 29 |
| YJL104W | 6.9 | 2.6 | 2275 |
| YHR170W | 14.7 | 1.5 | 80 |
| YER124C | 2.8 | 0.3 | 2 |
| YPR184W | 1.6 | 0.1 | 18 |
| YOR261C | 13.2 | 1.4 | 145 |
| YPL064C | 7.1 | 0.9 | 173 |
| YIL106W | 1.4 | 0.5 | 87 |
| YDL207W | 10.1 | 1.3 | 31 |
| YNR015W | 8.6 | 0.7 | 99 |
| YJL154C | 4.6 | 0.3 | 320 |
| YGR245C | 1.1 | 0.2 | 55 |
| YOL126C | 5.7 | 0.4 | 244 |
| YFR021W | 2.3 | 0.4 | 140 |
| YDL048C | 1.6 | 0.4 | 2 |
| YNL211C | 15.1 | 1.2 | 43 |
| YNL108C | 2.5 | 0.4 | -409 |
| YOR233W | 0.7 | 0.2 | 7 |
| YMR105C | 2.7 | 0.3 | 43 |
| YJL130C | 1.1 | 0.1 | 22 |
| YER002W | 2.2 | 0.5 | 37 |
| YER003C | 2.5 | 0.4 | 29 |
| YGL082W | 0.6 | 0.1 | 40 |
| YJL109C | 5 | 0.3 | 12 |
| YML096W | 3.3 | 0.3 | 34 |
| YBR122C | 30.8 | 5.2 | 28 |
| YNL292W | 2 | 0.9 | 65 |
| YKR036C | 2.7 | 0.3 | 34 |
| YGL184C | 3.3 | 0.3 | 2 |
| YGR117C | 2.3 | 0.2 | 5 |
| YHR058C | 1 | 0.3 | 26 |
| YOL025W | 4 | 0.6 | 36 |
| YDR411C | 2.6 | 0.5 | 82 |
| YGR040W | 43.8 | 3.4 | 83 |
| YJL122W | 3.7 | 0.9 | 56 |
| YGR111W | 2.8 | 0.3 | 108 |
| YMR091C | 3.4 | 0.7 | 116 |
| YGR204W | 1.9 | 0.2 | 86 |
| YLR309C | 3.5 | 0.4 | 219 |
| YOL111C | 14.9 | 3.7 | 176 |
| YJR126C | 1.3 | 0.1 | 14 |
| YPL178W | 5 | 1 | 87 |
| YCL010C | 21.8 | 3.2 | 42 |
| YGL087C | 4.9 | 1 | 51 |
| YGL200C | 86 | 4.9 | -152 |
| YDR171W | 2.9 | 0.5 | 72 |
| YOL141W | 12.6 | 1.2 | 23 |
| YPL075W | 0.7 | 0.2 | 36 |
| YDR500C | 5.7 | 1.1 | 53 |
| YGR198W | 3.6 | 0.3 | 16 |
| YDR525W-A | 203.9 | 23.6 | 224 |
| YNL007C | 30.1 | 6.2 | 274 |
| YDR302W | 88.9 | 7.7 | 305 |
| YBR230C | 10.4 | 0.8 | -184 |
| YDR298C | 10.3 | 0.9 | -113 |
| YBR152W | 2 | 0.8 | 45 |
| YDR295C | 1.2 | 0.2 | 41 |
| YOL087C | 0.8 | 0.2 | -394 |
| YPL199C | 1.3 | 0.4 | 15 |
| YMR024W | 21.8 | 1.9 | 94 |
| YDR222W | 5.4 | 0.5 | 76 |
| YLR095C | 7.4 | 1 | 12 |
| YDR079W | 217.6 | 16.6 | 15 |
| YIL131C | 1.7 | 0.3 | 31 |
| YBR010W | 4070.8 | 128 | -814 |
| YDR244W | 1.3 | 0.2 | 23 |
| YBR266C | 75.4 | 4 | 11 |
| YEL016C | 5 | 0.5 | 7 |
| YPR167C | 16.1 | 1 | 54 |
| YPL228W | 1.3 | 0.3 | 33 |
| YDL082W | 4.5 | 0.5 | 30 |
| YEL013W | 4.3 | 0.6 | 103 |
| YKL121W | 1.2 | 0.1 | 5 |
| YOR271C | 8.2 | 0.9 | 31 |
| YGL001C | 8.9 | 0.9 | 154 |
| YIL093C | 3.7 | 1 | -116 |
| YDL104C | 10.3 | 0.8 | 130 |
| YDL122W | 1.2 | 0.3 | 38 |
| YOR124C | 1.4 | 0.2 | 80 |
| YLR168C | 21.4 | 2.7 | 72 |
| YKL138C | 120.2 | 26.6 | -104 |
| YMR002W | 10.8 | 1 | -3826 |
| YKR048C | 3.4 | 0.9 | 108 |
| YJR129C | 33.6 | 3.5 | 13 |
| YKL022C | 1.1 | 0.2 | 66 |
| YDR482C | 2 | 1.1 | 2 |
| YGL043W | 1.7 | 0.5 | 184 |
| YNL223W | 6.5 | 0.7 | 38 |
| YMR166C | 3.1 | 0.8 | 32 |
| YKL140W | 10.7 | 1.3 | 29 |
| YGR169C | 6 | 0.5 | 86 |
| YFR031C | 2.2 | 0.2 | 41 |
| YHR008C | 8.4 | 0.5 | -392 |
| YDL085W | 4.8 | 0.5 | 48 |
| YHR155W | 2.5 | 0.2 | 35 |
| YNL230C | 13.3 | 2.6 | 13 |
| YNL030W | 1452.5 | 318 | 167 |
| YAL024C | 0.8 | 0.2 | 144 |
| YHR001W | 3.2 | 0.5 | 41 |
| YHR101C | 42.4 | 3.5 | 73 |
| YDL136W | 1347.3 | 64.3 | 142 |
| YNL271C | 0.7 | 0.2 | 79 |
| YCL063W | 2.7 | 0.6 | 31 |
| YLL026W | 6.9 | 0.6 | 61 |
| YGR037C | 102.5 | 14.2 | -164 |
| YER056C-A | 77.3 | 16.9 | 102 |
| YCL011C | 0.9 | 0.2 | 61 |
| YCL049C | 7 | 0.9 | 19 |
| YDR088C | 2.1 | 0.6 | 79 |
| YPL050C | 5.3 | 0.5 | 7 |
| YLR069C | 4.3 | 0.5 | -5393 |
| YBL075C | 3.4 | 0.4 | 59 |
| YMR102C | 1.8 | 0.3 | 23 |
| YDR081C | 0.6 | 0.1 | 85 |
| YER131W | 1.4 | 1.1 | 144 |
| YJL123C | 1.7 | 0.4 | 57 |
| YDL227C | 6.2 | 0.5 | 2 |
| YNR039C | 5.9 | 1 | 39 |
| YNL289W | 21.2 | 2.8 | 3 |
| YKL089W | 1.7 | 0.3 | 75 |
| YLR287C | 20.6 | 1.9 | 83 |
| YGR207C | 8.5 | 0.9 | -441 |
| YBR082C | 19.7 | 1.1 | 12 |
| YKL165C | 16.7 | 1.2 | 657 |
| YDL225W | 1.2 | 0.3 | 76 |
| YBR129C | 2 | 0.8 | 9 |
| YLR216C | 4.2 | 0.5 | 89 |
| YLR382C | 2.5 | 0.2 | 46 |
| YER034W | 21.9 | 3.9 | 60 |
| YHR209W | 31.1 | 2.9 | 40 |
| YOR101W | 0.7 | 0.2 | 106 |
| YDL060W | 0.8 | 0.3 | 27 |
| YIL017C | 2.1 | 0.2 | 7 |
| YGL172W | 3 | 0.5 | 51 |
| YMR285C | 11.2 | 1.5 | 22 |
| YNL126W | 2.5 | 0.2 | 32 |
| YJR013W | 10.7 | 0.9 | 38 |
| YER042W | 8.2 | 1 | 145 |
| YIL138C | 11.8 | 2.8 | 24 |
| YER087W | 6.8 | 0.6 | 3 |
| YPL161C | 11.4 | 0.6 | 39 |
| YOL076W | 6.2 | 0.4 | 139 |
| YNL262W | 0.8 | 0.1 | 190 |
| YBR264C | 65.7 | 7 | 57 |
| YIR010W | 2.7 | 0.6 | 22 |
| YER077C | 4.4 | 0.2 | 41 |
| YBL026W | 168.6 | 11.6 | 31 |
| YLR295C | 108.4 | 11 | 142 |
| YBR123C | 1.6 | 0.4 | 22 |
| YIL089W | 95.6 | 5 | 3 |
| YLR149C | 1.5 | 0.2 | 3 |
| YPL280W | 6.7 | 0.5 | -158 |
| YIL005W | 3.6 | 0.4 | 21 |
| YKL013C | 40.3 | 4.9 | -224 |
| YER145C | 5.5 | 0.5 | 183 |
| YIL046W | 3 | 0.6 | 3 |
| YHR143W | 12.9 | 0.9 | 1323 |
| YJL128C | 1.1 | 0.2 | 36 |
| YPL058C | 2.1 | 0.2 | -282 |
| YER059W | 2.7 | 0.5 | 52 |
| YER075C | 2 | 0.2 | 38 |
| YPR173C | 1.2 | 0.3 | 57 |
| YLR016C | 6.7 | 0.5 | 40 |
| YOL028C | 1.2 | 0.5 | 18 |
| YER056C | 13 | 0.8 | 30 |
| YJL144W | 2.2 | 0.8 | 15 |
| YBR111C | 6.3 | 1 | 194 |
| YGL229C | 4.4 | 0.4 | 39 |
| YJR154W | 5.7 | 0.9 | 30 |
| YNL154C | 1 | 0.2 | 18 |
| YHL039W | 5.9 | 0.4 | 160 |
| YGL071W | 1 | 0.2 | 23 |
| YJR098C | 4.5 | 0.7 | 54 |
| YDL195W | 0.8 | 0.1 | 26 |
| YHR030C | 1 | 0.3 | 62 |
| YKR041W | 8.1 | 1 | 37 |
| YER012W | 514.8 | 41.5 | 1131 |
| YML009C | 130.3 | 15.9 | 664 |
| YIL016W | 5.7 | 1 | 4664 |
| YMR292W | 1044.1 | 49.7 | 8 |
| YDR219C | 2.1 | 0.3 | 53 |
| YDL202W | 12.4 | 0.8 | -286 |
| YER142C | 76.1 | 6.3 | -353 |
| YDR483W | 8.1 | 0.7 | 2 |
| YOR340C | 0.7 | 0.2 | -453 |
| YPL181W | 1.8 | 0.4 | 29 |
| YDR516C | 3.8 | 0.4 | 31 |
| YIL031W | 0.7 | 0.1 | 14 |
| YOL006C | 3.3 | 0.4 | 35 |
| YBR193C | 7 | 1.1 | -1925 |
| YDR197W | 4.4 | 0.3 | 73 |
| YJL158C | 48.2 | 6.7 | 11 |
| YDR155C | 3.9 | 0.6 | -528 |
| YDR373W | 16.5 | 0.9 | 34 |
| YGR197C | 8 | 1.1 | 29 |
| YDR060W | 0.7 | 0.2 | 103 |
| YDL108W | 65.2 | 7.3 | 336 |
| YBL107C | 6.4 | 0.8 | 8 |
| YBR238C | 0.7 | 0.2 | 19 |
| YNL048W | 5.5 | 0.5 | 152 |
| YDR145W | 1.6 | 0.5 | 48 |
| YMR282C | 6.5 | 0.7 | 45 |
| YAL026C | 1.1 | 0.2 | 20 |
| YGL216W | 6.5 | 0.8 | 12 |
| YER185W | 20.7 | 1.1 | 33 |
| YGR268C | 13.6 | 1 | 42 |
| YLR358C | 3.2 | 0.5 | 169 |
| YHR037W | 6.6 | 0.8 | 132 |
| YNR048W | 3.4 | 0.4 | 30 |
| YBR164C | 221.9 | 16.5 | -120 |
| YKL004W | 41.7 | 3.4 | 94 |
| YML055W | 37.7 | 5.1 | 76 |
| YLR335W | 1.2 | 0.3 | 21 |
| YLR073C | 33.9 | 4.3 | 8 |
| YDL123W | 14.2 | 6.2 | 63 |
| YLR324W | 1.4 | 0.3 | 54 |
| YDL141W | 1.9 | 0.3 | 23 |
| YBR085W | 5.8 | 0.6 | 21 |
| YPR034W | 1.8 | 0.3 | 762 |
| YMR170C | 26 | 2.1 | -2079 |
| YIR038C | 11.8 | 1.1 | 66 |
| YPR004C | 12.6 | 0.9 | -164 |
| YKR081C | 36.4 | 4.8 | 166 |
| YGR132C | 7.6 | 0.9 | 27 |
| YER127W | 1.8 | 0.3 | 31 |
| YHR039C-A | 4.7 | 1.1 | -1639 |
| YNL255C | 72.7 | 4 | 135 |
| YDR182W | 16.2 | 1.7 | 6 |
| YJR024C | 96.3 | 5.2 | 14 |
| YLR231C | 10.3 | 0.7 | 64 |
| YJL168C | 2.8 | 0.3 | 2 |
| YPL090C | 8.1 | 1.1 | 100 |
| YLL002W | 6.6 | 0.5 | 150 |
| YLR372W | 9 | 0.5 | 91 |
| YCL039W | 3.7 | 0.4 | 23 |
| YNL309W | 0.9 | 0.2 | 69 |
| YOR052C | 8.7 | 0.9 | 103 |
| YPR101W | 25.3 | 3.2 | -154 |
| YOR249C | 3.5 | 0.3 | 18 |
| YJR068W | 13.4 | 0.9 | 31 |
| YKR090W | 1 | 0.2 | 98 |
| YNL129W | 10.4 | 0.9 | -2531 |
| YMR272C | 27.3 | 3.2 | 77 |
| YLR050C | 124.2 | 4.9 | 60 |
| YHR166C | 4.8 | 0.4 | 16 |
| YFL025C | 3 | 0.3 | 69 |
| YDL046W | 3.7 | 0.5 | 6 |
| YFR039C | 8 | 0.6 | 6 |
| YBR258C | 1.4 | 0.5 | 45 |
| YEL004W | 15.7 | 0.9 | 49 |
| YPL051W | 107.2 | 6.8 | 72 |
| YIL090W | 34.2 | 1.9 | 34 |
| YJL184W | 65.6 | 11.2 | -84 |
| YNL326C | 11.9 | 1 | 33 |
| YPR006C | 2.2 | 0.2 | 60 |
| YML034W | 1 | 0.2 | 31 |
| YNL091W | 0.4 | 0.1 | 32 |
| YDL099W | 3 | 0.6 | 63 |
| YNL101W | 1.1 | 0.3 | 26 |
| YJL121C | 121.2 | 12.3 | 1797 |
| YPL103C | 5 | 0.5 | 14 |
| YGR090W | 0.9 | 0.1 | 13266 |
| YGR081C | 4.1 | 0.8 | 109 |
| YEL015W | 3.7 | 0.6 | 61 |
| YIR035C | 6.8 | 0.5 | -101 |
| YLR079W | 1.5 | 0.5 | 33 |
| YKL150W | 41.2 | 4 | 53 |
| YMR260C | 5.7 | 1 | 32 |
| YLR189C | 0.9 | 0.2 | 220 |
| YDR068W | 8.6 | 2 | 100 |
| YHR057C | 6.7 | 1 | 7 |
| YBL091C-A | 5.8 | 1 | 15 |
| YBR239C | 3.5 | 0.5 | 16 |
| YLR063W | 31.9 | 2.6 | -384 |
| YDL100C | 3.6 | 0.8 | 19 |
| YFR007W | 10 | 0.7 | 68 |
| YDR479C | 1.7 | 0.4 | 52 |
| YMR089C | 1.6 | 0.4 | 29 |
| YGR017W | 7.7 | 2 | 180 |
| YOR199W | 61.4 | 8.9 | 372 |
| YOL064C | 5.9 | 0.9 | 31 |
| YNR027W | 15.2 | 0.9 | 234 |
| YDR370C | 7.7 | 0.6 | 54 |
| YGL113W | 2.2 | 0.4 | 16 |
| YOR359W | 1.2 | 0.4 | 22 |
| YMR194W | 73 | 8.1 | 214 |
| YBR126C | 4.1 | 0.3 | -70 |
| YDR288W | 2.4 | 0.8 | 22 |
| YMR016C | 0.6 | 0.1 | 3 |
| YJL131C | 9.8 | 1.3 | 50 |
| YLR419W | 1.8 | 0.2 | 26 |
| YDR319C | 11.8 | 0.9 | 44 |
| YJL200C | 1.2 | 0.2 | 34 |
| YJL025W | 2.3 | 0.3 | 15 |
| YOR246C | 6.9 | 0.9 | 17 |
| YDL070W | 2.6 | 0.4 | 9 |
| YDL063C | 4.7 | 0.4 | 35 |
| YPR156C | 5.8 | 0.8 | 35 |
| YMR280C | 0.6 | 0.1 | 11 |
| YIL098C | 86.6 | 7.4 | 84 |
| YDR128W | 1 | 0.2 | 3 |
| YDR052C | 1.3 | 0.3 | 12 |
| YGR168C | 2.3 | 0.7 | 58 |
| YGL076C | 51.5 | 3.6 | 113 |
| YMR039C | 0.9 | 0.3 | 46 |
| YML043C | 2.3 | 0.6 | 23 |
| YHR172W | 3.4 | 0.5 | 81 |
| YGL029W | 46.2 | 15.4 | 36 |
| YGL209W | 2.2 | 0.5 | 15 |
| YNL177C | 8.9 | 0.9 | 151 |
| YDR216W | 0.6 | 0.1 | 58 |
| YLR027C | 5.7 | 0.4 | 205 |
| YDR305C | 8.5 | 1 | 126 |
| YDL160C | 4.9 | 0.7 | 176 |
| YNL152W | 1.5 | 0.4 | 26 |
| YGL080W | 403.5 | 16.9 | 37 |
| YKL141W | 90.9 | 9.5 | 24 |
| YJR025C | 36.1 | 5.3 | 185 |
| YCR020C | 63 | 5.6 | 65 |
| YML109W | 0.9 | 0.2 | 47 |
| YKL028W | 3.1 | 0.6 | 28 |
| YGL044C | 0.6 | 0.2 | 71 |
| YOR174W | 18.2 | 3.1 | -157 |
| YLR386W | 4 | 0.3 | 60 |
| YPL029W | 4.9 | 0.4 | 44 |
| YBR197C | 3.3 | 0.9 | 93 |
| YJR139C | 3.7 | 0.4 | 56 |
| YEL017C-A |  |  | -167 |
| YOR065W | 11.7 | 1.1 | 161 |
| YPL106C | 18 | 2 | 101 |
| YMR178W | 14.9 | 1 | -102 |
| YPL126W | 1.2 | 0.1 | 55 |
| YJR109C | 3.3 | 0.3 | 7 |
| YMR204C | 1.9 | 0.4 | 30 |
| YNL051W | 12.8 | 1.1 | 28 |
| YBR104W | 4.3 | 0.5 | 16 |
| YER174C | 6 | 0.5 | -146 |
| YLR110C | 5.8 | 1.2 | 26 |
| YOR283W | 53 | 4 | 68 |
| YJL088W | 5.8 | 0.7 | 49 |
| YMR011W | 8.8 | 0.8 | -961 |
| YNL036W | 9.7 | 0.9 | 14 |
| YCR053W | 4.8 | 0.3 | 148 |
| YLR042C | 27.1 | 3.5 | -94 |
| YBL015W | 3.2 | 0.3 | 6 |
| YNL238W | 0.9 | 0.2 | 93 |
| YOL081W | 1.2 | 0.1 | 13 |
| YKL086W | 32.9 | 9.7 | 16 |
| YNL102W | 0.9 | 0.1 | -4088 |
| YGR089W | 1.3 | 0.2 | 16 |
| YPL188W | 8.6 | 1.4 | 86 |
| YER112W | 0.6 | 0.2 | 217 |
| YPR127W | 11.3 | 0.8 | 92 |
| YMR269W | 5.2 | 1 | 23 |
| YDR073W | 7.2 | 1.2 | 30 |
| YGR033C | 4.8 | 0.9 | 26 |
| YAL058W | 6.8 | 0.7 | 71 |
| YMR305C | 6.1 | 0.9 | 18 |
| YAR007C | 5.6 | 1 | 35 |
| YBR162C | 2 | 0.3 | 67 |
| YOR167C | 73.1 | 19.3 | 117 |
| YNR074C | 9.9 | 0.8 | 49 |
| YBR087W | 5.4 | 0.5 | 138 |
| YML067C | 7 | 0.9 | 166 |
| YOR043W | 5.5 | 1.1 | 41 |
| YJL070C | 2.9 | 0.4 | 63 |
| YOR258W | 17.6 | 0.8 | 50 |
| YGR121C | 2.2 | 0.8 | 36 |
| YDL025C | 1.1 | 0.3 | 32 |
| YOL043C | 7.8 | 0.9 | -3648 |
| YGL055W | 10.2 | 0.8 | 193 |
| YPL053C | 6.2 | 0.7 | 27 |
| YMR029C | 2.2 | 0.4 | 29 |
| YDR177W | 5.6 | 1 | 45 |
| YER008C | 1 | 0.2 | 29 |
| YPL202C | 9.1 | 1.4 | 35 |
| YGR141W | 5.6 | 0.5 | 36 |
| YBR065C | 9.5 | 1.9 | 41 |
| YPL198W | 51.9 | 3.6 | 82 |
| YLL021W | 0.6 | 0.2 | -162 |
| YMR162C | 1.3 | 0.2 | 47 |
| YHR085W | 18.2 | 0.9 | -320 |
| YMR117C | 9.1 | 0.9 | 74 |
| YPL007C | 3 | 0.2 | 44 |
| YOR270C | 7.1 | 0.6 | 44 |
| YMR264W | 2.4 | 0.9 | 25 |
| YOL052C | 8.4 | 0.8 | 111 |
| YDR007W | 4.9 | 0.5 | 52 |
| YJR110W | 3.8 | 0.6 | 19 |
| YOR335C | 6.4 | 0.5 | 108 |
| YDR264C | 3.1 | 0.4 | 90 |
| YNL011C | 3 | 0.3 | 28 |
| YMR322C | 13.5 | 0.9 | 3 |
| YJL214W | 3.2 | 0.5 | -75 |
| YBR212W | 0.6 | 0.1 | 28 |
| YDR279W | 2.8 | 0.4 | 31 |
| YMR149W | 9.8 | 1 | 329 |
| YBR274W | 5.7 | 0.5 | 30 |
| YOR085W | 37.5 | 3 | 117 |
| YPR154W | 2.1 | 0.5 | 384 |
| YLR139C | 4.9 | 0.4 | -3308 |
| YKR025W | 1.6 | 0.4 | 43 |
| YDR210W |  |  | -1610 |
| YGR231C | 37.6 | 3.8 | 39 |
| YIL112W | 0.7 | 0.1 | 15 |
| YBL061C | 1.6 | 0.3 | 10 |
| YKR052C | 39.8 | 5.7 | 62 |
| YCL045C | 3.1 | 0.3 | 48 |
| YGR211W | 9 | 0.9 | 13 |
| YPL155C | 0.9 | 0.2 | -394 |
| YGL195W | 1.8 | 0.1 | 17 |
| YJL033W | 13.1 | 2.8 | 38 |
| YPR104C | 1.2 | 0.3 | 40 |
| YNL061W | 9.8 | 2.1 | 886 |
| YDL145C | 3.3 | 0.3 | 92 |
| YPR035W | 5.8 | 0.8 | 73 |
| YNL172W | 0.9 | 0.1 | 31 |
| YOR078W | 1.7 | 1.1 | 83 |
| YLR433C | 3.6 | 0.3 | 32 |
| YNL252C | 5.3 | 0.9 | 43 |
| YOR195W | 1.5 | 0.3 | 25 |
| YDL097C | 13.4 | 0.8 | 51 |
| YPL122C | 2 | 0.3 | 46 |
| YML097C | 1.8 | 0.3 | 85 |
| YPR022C | 0.5 | 0.1 | 20 |
| YKR093W | 6.1 | 1.4 | 23 |
| YGL246C | 5.8 | 0.3 | 82 |
| YDR179C | 205.1 | 16.8 | 40 |
| YHR079C | 2.5 | 0.3 | 32 |
| YMR165C | 1.1 | 0.2 | 72 |
| YCR023C | 4.8 | 0.4 | 48 |
| YGR205W | 29.7 | 2.3 | 472 |
| YJR006W | 2.6 | 0.3 | 43 |
| YPL101W | 3.4 | 0.5 | 75 |
| YPR147C | 16 | 0.9 | 8 |
| YLR143W | 6.5 | 0.8 | 27 |
| YPL038W | 4.7 | 1 | 95 |
| YML132W | 7.9 | 0.8 | 33 |
| YML086C | 3.1 | 0.3 | 39 |
| YGL111W | 9.2 | 0.9 | 48 |
| YBR049C | 0.5 | 0.2 | 12 |
| YFL023W | 1.8 | 0.5 | 13 |
| YNL209W | 11.4 | 0.9 | 712 |
| YPL254W | 0.7 | 0.2 | 28 |
| YOR098C | 0.4 | 0.1 | 21 |
| YDL212W | 16.1 | 3.4 | 95 |
| YBL047C | 0.5 | 0.2 | 74 |
| YJL041W | 1.2 | 0.3 | 13 |
| YLR120C | 6 | 0.6 | 11 |
| YGR074W | 4.6 | 0.5 | 128 |
| YMR290W-A | 72.5 | 8.2 | 209 |
| YFL047W | 4 | 0.3 | 117 |
| YHR162W | 18.4 | 1.1 | 42 |
| YMR287C | 3.9 | 0.3 | 35 |
| YMR186W | 26.2 | 2 | 109 |
| YHR034C | 4.6 | 0.5 | 62 |
| YCL059C | 7.9 | 0.9 | 191 |
| YNL293W | 0.6 | 0.2 | 23 |
| YPR161C | 0.6 | 0.2 | 12 |
| YMR263W | 0.5 | 0.2 | -263 |
| YKL114C | 24.3 | 1.9 | 60 |
| YMR006C | 1.8 | 0.2 | 2 |
| YBR030W | 7.7 | 0.6 | 37 |
| YNL106C | 1.3 | 0.2 | -98 |
| YHR060W | 49 | 5.6 | 52 |
| YPL017C | 6.5 | 0.9 | 29 |
| YDL101C | 4 | 0.3 | 102 |
| YDR013W | 12.9 | 1 | 67 |
| YHR129C | 14.3 | 1 | -290 |
| YDR235W | 7.8 | 0.5 | 90 |
| YHR103W | 1.1 | 0.2 | 7 |
| YPR047W | 9.3 | 0.8 | 303 |
| YGR061C | 1 | 0.1 | 33 |
| YLL011W | 9.9 | 1.3 | 29 |
| YDR440W | 0.9 | 0.3 | 63 |
| YDL053C | 1.7 | 0.5 | 44 |
| YIL121W | 8.1 | 0.9 | 52 |
| YLR417W | 4.9 | 0.5 | 166 |
| YMR077C | 8.8 | 0.5 | 124 |
| YKL032C | 0.9 | 0.3 | 10 |
| YKL204W | 0.3 | 0.1 | 25 |
| YNL092W | 11.8 | 0.7 | 12 |
| YBR070C | 21.8 | 1.1 | 43 |
| YDR275W | 17.4 | 2 | 73 |
| YFR008W | 14.2 | 0.9 | 36 |
| YFR036W | 2.9 | 0.8 | 2 |
| YJR059W | 0.5 | 0.2 | 50 |
| YPL162C | 4.8 | 0.9 | 2 |
| YNL078W | 4.1 | 1.1 | 37 |
| YGR178C | 0.6 | 0.2 | -780 |
| YOL039W | 257.2 | 13 | 159 |
| YGR048W | 6.4 | 0.5 | 70 |
| YGR075C | 1.4 | 0.3 | 29 |
| YLR363W-A | 18.7 | 14.1 | 302 |
| YPL005W | 3.7 | 0.2 | 11 |
| YIL105C | 0.6 | 0.2 | 21 |
| YNL163C | 4.9 | 0.4 | 2 |
| YMR027W | 3.2 | 0.5 | 26 |
| YPL263C | 1.9 | 0.3 | 29 |
| YIL045W | 1 | 0.2 | 2 |
| YKL157W | 2.1 | 0.3 | 36 |
| YDR412W | 11 | 3.3 | 82 |
| YCL031C | 11.3 | 0.8 | 101 |
| YOR182C | 4.5 | 1.2 | 74 |
| YER110C | 3.4 | 0.2 | 130 |
| YBR159W | 26.1 | 0.9 | 988 |
| YGL110C | 3.6 | 0.6 | 21 |
| YBR023C | 1.2 | 0.2 | 49 |
| YDL105W | 5.2 | 0.6 | 25 |
| YPL043W | 3 | 0.6 | 22 |
| YNL284C | 39.2 | 3.9 | 109 |
| YHR072W | 6.8 | 0.4 | 18 |
| YAL032C | 2.2 | 0.4 | 474 |
| YJL062W | 7.5 | 0.4 | 29 |
| YJR055W | 28.8 | 2.5 | 82 |
| YBR287W | 16.7 | 1.9 | 29 |
| YOR104W | 4.5 | 0.6 | 69 |
| YCR017C | 5.5 | 0.3 | 689 |
| YMR031C | 4.9 | 0.9 | 9 |
| YDL165W | 58.1 | 6.8 | -83 |
| YML022W | 92.1 | 6.2 | -112 |
| YJL173C | 7.4 | 0.5 | 3196 |
| YCR027C | 32.5 | 4.7 | 34 |
| YDR056C | 81.1 | 7.4 | 127 |
| YHR004C | 3 | 0.6 | 97 |
| YDR159W | 1.4 | 0.2 | 35 |
| YGL223C | 17.4 | 1.2 | 41 |
| YBL055C | 6.1 | 0.5 | 58 |
| YBL045C | 3.2 | 0.4 | 291 |
| YBR043C | 3.7 | 0.4 | 30 |
| YJR017C | 70.9 | 11.8 | -299 |
| YLL050C | 4.7 | 0.6 | -226 |
| YDR268W | 4.8 | 0.8 | -112 |
| YKL018W | 5 | 0.5 | 26 |
| YGR021W | 3.8 | 0.4 | -4890 |
| YPL256C | 1.6 | 0.2 | 5 |
| YML125C | 30.5 | 2.8 | -146 |
| YDL124W | 6.9 | 0.5 | 55 |
| YOL022C | 8.5 | 0.8 | 44 |
| YIL148W | 153.5 | 16.4 | 187 |
| YLR067C | 5.4 | 0.4 | 46 |
| YGR068C | 1.8 | 0.4 | 25 |
| YDR148C | 2.9 | 0.3 | 51 |
| YGR158C | 3 | 0.3 | 38 |
| YNL029C | 3.9 | 0.4 | 16 |
| YGR271C-A | 52.3 | 4.6 | -593 |
| YMR314W | 47.5 | 5.8 | -68 |
| YML064C | 14.9 | 3.6 | 26 |
| YHR158C | 0.7 | 0.1 | -747 |
| YDR404C | 113.2 | 19.2 | 35 |
| YDL036C | 2.3 | 0.3 | 129 |
| YBR017C | 2.1 | 0.3 | 41 |
| YPR066W | 12.8 | 0.8 | 30 |
| YJL005W | 0.6 | 0.1 | 75 |
| YHR049W | 6 | 0.4 | 49 |
| YNR030W | 16.2 | 0.8 | 59 |
| YBR273C | 2.4 | 0.8 | 47 |
| YKL152C | 69.4 | 5.8 | -738 |
| YHR023W | 1.4 | 0.1 | 42 |
| YDL030W | 10.4 | 0.8 | 21 |
| YMR127C | 2.4 | 0.9 | 6 |
| YJL094C | 13.2 | 1.2 | 19 |
| YPL002C | 11.4 | 0.5 | 96 |
| YOL125W | 0.8 | 0.2 | 61 |
| YLR330W | 1.2 | 0.6 | 228 |
| YGR156W | 4.5 | 1.1 | 77 |
| YMR026C | 4.5 | 0.8 | 33 |
| YOL070C | 1.7 | 0.7 | 53 |
| YDR129C | 9.5 | 0.6 | 96 |
| YOR033C | 4 | 0.4 | 2 |
| YPL022W | 2.1 | 0.3 | 67 |
| YMR230W | 179.4 | 19.5 | 1448 |
| YDR368W | 10.3 | 0.9 | 74 |
| YBR132C | 20.1 | 1.7 | 89 |
| YJR073C | 93.7 | 12.8 | 203 |
| YGL142C | 71.9 | 2.8 | 26 |
| YHL018W | 102.4 | 29.5 | 18 |
| YPR019W | 1.9 | 0.6 | -260 |
| YPL235W | 16.3 | 0.9 | 250 |
| YPL128C | 1 | 0.3 | 46 |
| YIL052C | 77.1 | 16.9 | 69 |
| YPR129W | 1.2 | 0.4 | 30 |
| YLR132C | 12.2 | 0.9 | 29 |
| YBR084W | 7.3 | 0.6 | 73 |
| YEL012W | 4.5 | 0.5 | 87 |
| YHR087W | 5.4 | 1 | 45 |
| YOR044W | 35.5 | 6.5 | 7 |
| YHL009C | 2.9 | 0.6 | 26 |
| YDR137W | 4.6 | 0.6 | 32 |
| YDR391C | 3.7 | 0.3 | 40 |
| YCR009C | 16.6 | 1.1 | 50 |
| YNR006W | 1.5 | 0.3 | 7 |
| YBR039W | 6.6 | 0.8 | -169 |
| YFR044C | 18.1 | 0.9 | 82 |
| YML023C | 6.1 | 0.4 | 36 |
| YDL155W | 7.8 | 0.7 | 20 |
| YBR142W | 5.5 | 0.9 | 127 |
| YDR158W | 43.5 | 3.9 | 105 |
| YKL085W | 6.1 | 0.7 | 167 |
| YOL117W | 6.4 | 0.7 | 51 |
| YMR218C | 6.2 | 0.5 | 131 |
| YFL016C | 7.5 | 0.8 | 53 |
| YER037W | 31.1 | 2.5 | 36 |
| YPL241C | 20.7 | 2.3 | 44 |
| YER133W | 41.9 | 2.9 | 61 |
| YJL068C | 7.2 | 0.9 | 243 |
| YIL068C | 3.6 | 0.4 | 28 |
| YMR182C | 7.3 | 2 | -28810 |
| YOR016C | 76.7 | 5.5 | 606 |
| YMR231W | 2.3 | 0.2 | 64 |
| YKL219W | 6.3 | 0.8 | 871 |
| YKR003W | 9 | 0.9 | 81 |
| YPR112C | 1.4 | 0.2 | 16 |
| YBR216C | 6.4 | 0.5 | 23 |
| YNL187W | 6.5 | 0.5 | 33 |
| YER016W | 0.8 | 0.2 | 56 |
| YML031W | 1.5 | 0.2 | 98 |
| YGL077C | 5.5 | 0.5 | 26 |
| YJL115W | 9.1 | 3.9 | 123 |
| YDR528W | 7.8 | 1.6 | 55 |
| YPL060W | 25 | 2.1 | 29 |
| YMR068W | 1.2 | 0.2 | 58 |
| YMR167W | 5 | 0.5 | 10 |
| YOL159C-A | 337.9 | 19.6 | 2 |
| YDL125C | 10.3 | 1.2 | 267 |
| YMR259C | 9.3 | 0.5 | 89 |
| YKL088W | 1.1 | 0.3 | 59 |
| YPR119W | 1.7 | 0.3 | 22 |
| YOR172W | 2.3 | 0.3 | 92 |
| YOR243C | 2.3 | 0.3 | 40 |
| YJR035W | 1.6 | 0.2 | -68 |
| YHR015W | 4.5 | 0.7 | 2 |
| YNL147W | 38 | 8.8 | -153 |
| YGL090W | 3.6 | 0.8 | 12 |
| YGR138C | 7.8 | 0.8 | 26 |
| YKL211C | 8.1 | 0.8 | 518 |
| YMR128W | 1 | 0.4 | 393 |
| YNL098C | 1 | 0.3 | 25 |
| YJR113C | 62.5 | 6.6 | 38 |
| YCR038C | 14 | 0.9 | 122 |
| YOR275C | 9.1 | 0.6 | 27 |
| YER090W | 22.4 | 2.1 | 39 |
| YNL277W | 18 | 2.1 | 23 |
| YMR049C | 1.4 | 0.3 | 17 |
| YOR269W | 2.7 | 0.3 | 10 |
| YNL046W | 24.6 | 5 | 29 |
| YER105C | 1.5 | 0.2 | 33 |
| YLR388W | 145.9 | 18.2 | -90 |
| YML057W | 2.6 | 0.3 | 47 |
| YDR065W | 5 | 0.5 | 31 |
| YBR077C | 8 | 0.8 | 539 |
| YNL311C | 4.8 | 0.5 | 14 |
| YBR201W | 17 | 0.9 | 3 |
| YHR199C | 23.7 | 3.9 | 88 |
| YGL011C | 11.2 | 0.9 | 73 |
| YLR181C | 12.7 | 2.7 | 32 |
| YNL186W | 0.7 | 0.2 | 54 |
| YAL039C | 5.7 | 1 | 2 |
| YNL272C | 1.5 | 0.5 | 46 |
| YGR238C | 3.6 | 0.3 | 39 |
| YKL142W | 45 | 4.8 | 48 |
| YHR182W | 1.6 | 0.2 | 93 |
| YLL006W | 1.6 | 0.3 | 42 |
| YNL224C | 1.9 | 0.4 | 31 |
| YBR272C | 7.1 | 0.8 | 15 |
| YER030W | 1.6 | 0.6 | -75 |
| YJR010W | 6.3 | 0.4 | 44 |
| YDR265W | 22.6 | 3.7 | -677 |
| YGL027C | 3.6 | 0.4 | 31 |
| YOR086C | 1.2 | 0.2 | 318 |
| YLR150W | 4.2 | 1.5 | 184 |
| YBR080C | 2.7 | 0.3 | 37 |
| YKR011C | 1.3 | 0.7 | 71 |
| YJL052W | 13.6 | 0.9 | 85 |
| YDR425W | 3.3 | 0.5 | 54 |
| YPL152W | 29.8 | 2.8 | -1019 |
| YIL076W | 42.2 | 2.5 | 20 |
| YPR080W | 7.6 | 0.9 | 56 |
| YHR208W | 7.4 | 1 | 897 |
| YBL035C | 5.7 | 0.6 | 52 |
| YDR345C | 3.2 | 0.4 | -185 |
| YKL081W | 5.9 | 0.5 | 105 |
| YPL068C | 8.8 | 1.4 | 33 |
| YNL067W | 9.6 | 1 | -1032 |
| YJL036W | 18.1 | 2.5 | 201 |
| YGL120C | 4.5 | 0.9 | 32 |
| YBR166C | 7.8 | 0.8 | 79 |
| YPR057W | 22.1 | 2.4 | -175 |
| YDR059C | 171.9 | 11.7 | 77 |
| YCL014W | 0.9 | 0.2 | 8 |
| YML005W | 4.2 | 0.3 | 63 |
| YDR152W | 21.8 | 6.1 | 39 |
| YOR260W | 4.1 | 0.6 | 1041 |
| YKR068C | 105.6 | 6.6 | 44 |
| YGR010W | 2.6 | 0.8 | 65 |
| YDR434W | 2.1 | 0.2 | 83 |
| YLR429W | 6.2 | 0.7 | 23 |
| YJR147W | 28.8 | 2.1 | 42 |
| YHR031C | 2.3 | 0.4 | 8 |
| YBR249C | 1.9 | 0.3 | 71 |
| YIL064W | 2.2 | 0.4 | 90 |
| YIL002C | 4.9 | 0.3 | 23 |
| YIL135C | 1.2 | 0.4 | 20 |
| YCL008C | 2.1 | 0.4 | 46 |
| YML007C-A | 4494.2 | 128 | 2981 |
| YER178W | 8.7 | 0.5 | 77 |
| YOR129C | 1.3 | 0.2 | 24 |
| YKL068W | 0.3 | 0.1 | 20 |
| YAL049C | 39.1 | 3.2 | 560 |
| YGR084C | 29.4 | 2.4 | 82 |
| YPR175W | 4.1 | 0.4 | 62 |
| YPL151C | 6.8 | 0.5 | 124 |
| YML102W | 3.3 | 0.4 | 45 |
| YPR036W | 3.7 | 0.3 | 223 |
| YCR048W | 20.2 | 0.9 | 36 |
| YDR348C | 1.1 | 0.4 | 31 |
| YDR090C | 76.3 | 8.9 | 87 |
| YMR209C | 4.6 | 0.4 | 52 |
| YIL160C | 18.8 | 2.1 | 30 |
| YHR179W | 5.1 | 0.5 | 107 |
| YKL008C | 26.3 | 3.6 | 77 |
| YDL115C | 3.9 | 0.8 | 40 |
| YOL054W | 9.9 | 1.9 | 54 |
| YHR149C | 0.8 | 0.2 | 9 |
| YLL019C | 1.7 | 0.2 | 34 |
| YJR096W | 7 | 0.5 | 63 |
| YML072C | 0.6 | 0.1 | 32 |
| YKR064W | 2.1 | 0.2 | 109 |
| YPL146C | 5.6 | 1.5 | 33 |
| YBL014C | 2 | 0.4 | 33 |
| YBR162W-A | 57.6 | 10.3 | 4 |
| YER136W | 13.6 | 0.9 | -85 |
| YOL101C | 23.7 | 2.7 | 19 |
| YNL097C | 2.2 | 0.6 | 49 |
| YKR082W | 2 | 0.2 | 15 |
| YLR219W | 1 | 0.3 | 92 |
| YCR060W | 139.8 | 12.2 | 47 |
| YHR188C | 3.1 | 0.4 | 42 |
| YGL047W | 14.7 | 1 | -3088 |
| YOR312C | 115.2 | 12.7 | -317 |
| YOL031C | 2.2 | 0.4 | 779 |
| YDL110C | 12.8 | 2.3 | 19 |
| YKL060C | 5.1 | 0.5 | 104 |
| YJL157C | 7.5 | 0.7 | 10 |
| YHR154W | 2.8 | 0.3 | 41 |
| YLR345W | 6.4 | 0.8 | 73 |
| YKL010C | 1.4 | 0.3 | 11 |
| YOR150W | 85.7 | 13.4 | -123 |
| YNL317W | 1.8 | 0.3 | 38 |
| YLR244C | 4.7 | 0.9 | 669 |
| YNR011C | 7.2 | 0.9 | 7 |
| YDR488C | 2.3 | 0.3 | 55 |
| YPL032C | 0.5 | 0.2 | 24 |
| YGR135W | 117 | 6.8 | 47 |
| YPR049C | 1.4 | 0.2 | 73 |
| YIR024C | 6.4 | 0.5 | 413 |
| YDR046C | 3.2 | 0.4 | 27 |
| YER049W | 12.6 | 1.5 | 35 |
| YHL013C | 19.4 | 3.5 | 50 |
| YHL019C | 4.7 | 0.6 | 2 |
| YBL078C | 120.4 | 14.8 | 289 |
| YPR081C | 3.9 | 0.4 | 38 |
| YGR200C | 4.4 | 0.3 | 73 |
| YKR013W | 15 | 1 | 32 |
| YLR176C | 1 | 0.2 | 53 |
| YLR439W | 25.7 | 2.3 | -384 |
| YER134C | 5 | 0.4 | -313 |
| YGL156W | 2.1 | 0.2 | 52 |
| YGR020C | 58.4 | 5.8 | 49 |
| YDR379W | 0.8 | 0.2 | -310 |
| YBR163W | 7.8 | 0.8 | 27 |
| YHR066W | 6 | 0.8 | 30 |
| YJL222W | 1.9 | 0.2 | -218 |
| YDR335W | 2.2 | 0.2 | -2587 |
| YER017C | 1.2 | 0.3 | 45 |
| YBR093C | 8.2 | 0.6 | 18 |
| YBR227C | 7.7 | 0.8 | 83 |
| YNL127W | 3.7 | 0.7 | 84 |
| YJR042W | 3.5 | 0.4 | 173 |
| YOL061W | 4 | 0.5 | 124 |
| YML030W | 134.3 | 16.3 | 63 |
| YNL027W | 0.6 | 0.2 | 10 |
| YOL104C | 7.2 | 0.5 | 13 |
| YLR406C | 256.7 | 23.8 | 265 |
| YMR261C | 1.5 | 0.1 | 22 |
| YMR267W | 2.5 | 0.3 | 357 |
| YDR221W | 3.1 | 0.3 | 26 |
| YPL169C | 2.1 | 0.2 | 41 |
| YJL110C | 1.4 | 0.3 | 23 |
| YOR316C | 14.4 | 2.4 | 18 |
| YBL069W | 3.7 | 0.3 | 6 |
| YML124C | 5 | 0.5 | 448 |
| YCR082W | 15.5 | 1 | 21 |
| YGL234W | 1.2 | 0.1 | 162 |
| YML028W | 10.2 | 1 | -129 |
| YKL047W | 3.1 | 0.3 | 177 |
| YMR311C | 10 | 3.1 | 53 |
| YJL125C | 18.1 | 3 | 905 |
| YIR015W | 3.1 | 1 | 17 |
| YKL035W | 4.2 | 0.3 | 57 |
| YLR385C | 15.4 | 1 | -312 |
| YCL044C | 2.8 | 0.5 | 99 |
| YPL004C | 1 | 0.6 | 389 |
| YGL255W | 1 | 0.3 | 33 |
| YER111C | 0.5 | 0.1 | 141 |
| YNL016W | 1.6 | 0.4 | 72 |
| YAL046C | 17.7 | 1.2 | 115997 |
| YKL065C | 18.4 | 3.5 | 1256 |
| YER168C | 8.3 | 0.6 | 159 |
| YKL064W | 1.5 | 0.4 | 78 |
| YBR229C | 1.9 | 0.2 | 27 |
| YDL085C-A | 35.7 | 24.3 | 73 |
| YLR310C | 1.1 | 0.2 | 16 |
| YIL154C | 18.4 | 5.4 | -91 |
| YER155C | 1 | 0.1 | 2616 |
| YML082W | 1.2 | 0.3 | 58 |
| YNL052W | 40.2 | 6.5 | 250 |
| YER015W | 24.5 | 1.6 | 26 |
| YNL044W | 61.4 | 8 | 44 |
| YLR432W | 2.9 | 0.3 | 650 |
| YGR196C | 0.6 | 0.2 | 26 |
| YDR312W | 5.1 | 0.8 | 50 |
| YGR099W | 5.3 | 0.3 | 248 |
| YBR060C | 1.2 | 0.4 | 25 |
| YLR220W | 1.3 | 0.5 | 2 |
| YBR073W | 4.5 | 0.5 | 30 |
| YIL118W | 2 | 0.5 | 171 |
| YGL045W | 2.2 | 0.3 | 15 |
| YDR392W | 15.5 | 3.2 | 329 |
| YHR193C | 7.7 | 0.9 | 263 |
| YER024W | 0.6 | 0.2 | 83 |
| YLR305C | 1.4 | 0.1 | 27 |
| YOR254C | 17.8 | 1.5 | -72 |
| YGR148C | 91.5 | 10.3 | 54 |
| YMR242C | 115.2 | 12.7 | 137 |
| YLR441C | 43.8 | 6.4 | 334 |
| YLL034C | 1.8 | 0.4 | 20 |
| YER144C | 3.2 | 0.4 | 14 |
| YLR059C | 51.3 | 4.4 | 66 |
| YMR180C | 2.4 | 0.4 | 114 |
| YER170W | 101.8 | 16.4 | 135 |
| YER162C | 3.5 | 0.4 | 57 |
| YHR117W | 3.8 | 0.4 | 98 |
| YOL005C | 29.6 | 3.8 | -167 |
| YML101C | 25.8 | 7.5 | -90 |
| YNL080C | 6.8 | 0.8 | 9 |
| YNL274C | 24.2 | 2.4 | 50 |
| YLR117C | 7.5 | 0.4 | 31 |
| YML108W | 44.9 | 9.6 | 59 |
| YDR201W | 32.6 | 6.5 | -84 |
| YPL119C | 0.6 | 0.2 | 18 |
| YFR055W | 15.5 | 1 | 30 |
| YNL300W | 352.3 | 46.5 | -146 |
| YLR354C | 49.2 | 4.2 | 136 |
| YJR074W | 7.7 | 1 | 29 |
| YLR355C | 2.9 | 0.3 | 350 |
| YNL169C | 7.6 | 0.7 | 16 |
| YKL074C | 0.9 | 0.2 | 74 |
| YDR386W | 6 | 0.7 | 20 |
| YKR046C | 25.7 | 3.7 | 43 |
| YLL022C | 0.7 | 0.3 | 188 |
| YPL081W | 109.1 | 10 | 169 |
| YMR268C | 4.2 | 0.3 | 52 |
| YAR003W | 5.7 | 0.4 | 42 |
| YER093C-A | 30 | 14.3 | 1178 |
| YML095C | 6.4 | 0.7 | 42 |
| YLR257W | 4.3 | 0.7 | 21 |
| YMR213W | 2.2 | 0.7 | 28 |
| YCR016W | 1.4 | 0.5 | 68 |
| YMR291W | 1.9 | 0.4 | 14 |
| YHL024W | 0.5 | 0.1 | 158 |
| YBR244W | 7.9 | 0.6 | 168 |
| YGL012W | 6.4 | 0.5 | 198 |
| YAL036C | 42.4 | 3.3 | 57 |
| YER020W | 1 | 0.3 | 16 |
| YDR447C | 8.1 | 1.1 | 140 |
| YDR499W | 10.6 | 0.7 | 14 |
| YML010W | 0.5 | 0.1 | 3 |
| YBR125C | 1.1 | 0.2 | 62 |
| YML018C | 20.9 | 2.9 | 80 |
| YLL018C-A | 4 | 1.1 | 5 |
| YLR449W | 1 | 0.5 | 11 |
| YAR015W | 3.8 | 0.4 | 77 |
| YMR106C | 6.3 | 1.5 | 16 |
| YLR126C | 11 | 0.9 | 27 |
| YDR033W | 42.6 | 3.1 | 208 |
| YPL041C | 24.8 | 1.1 | 54 |
| YNL206C | 2.4 | 0.7 | -184 |
| YER122C | 1.3 | 0.3 | 56 |
| YHR189W | 14.3 | 1.2 | 82 |
| YOR165W | 19.2 | 1.7 | 135 |
| YLR056W | 5.9 | 0.4 | 45 |
| YOR256C | 1.6 | 0.2 | 2 |
| YIL130W | 0.8 | 0.2 | 3 |
| YPL242C | 3.3 | 0.2 | -614 |
| YPL034W | 289.7 | 33.1 | -301 |
| YPL211W | 15.2 | 1 | 40 |
| YLR393W | 26.7 | 3.2 | 126 |
| YHR111W | 5.7 | 0.8 | 57 |
| YOR356W | 25 | 2.2 | 24 |
| YJL055W | 1.7 | 0.5 | 32 |
| YLL028W | 3.6 | 0.5 | 181 |
| YDR168W | 8.7 | 1.7 | 454 |
| YDL173W | 1 | 0.2 | 72 |
| YHR050W | 2.9 | 0.3 | 29 |
| YBR261C | 12.5 | 1.1 | -240 |
| YOR062C | 3.9 | 1 | 32 |
| YJL030W | 7.1 | 0.9 | 24 |
| YMR037C | 0.7 | 0.2 | 2 |
| YOL080C | 1.8 | 0.5 | 97 |
| YIL048W | 5 | 0.7 | -87 |
| YGR183C | 166.9 | 6.5 | 2048 |
| YHR077C | 1.9 | 0.2 | 2 |
| YOR026W | 7.1 | 0.7 | 22 |
| YMR109W | 4.3 | 0.7 | 168 |
| YLR448W | 8.2 | 0.6 | -413 |
| YER132C | 0.5 | 0.1 | 3 |
| YPR179C | 1.9 | 0.3 | 30 |
| YOR164C | 14.4 | 0.9 | 272 |
| YGR208W | 6.8 | 0.9 | 59 |
| YGR092W | 3.2 | 0.5 | 11 |
| YER183C | 160.7 | 9 | -214 |
| YFR016C | 0.6 | 0.2 | 119 |
| YPR189W | 1.9 | 0.2 | 32 |
| YJL198W | 13.4 | 1.3 | -244 |
| YHL003C | 30.8 | 3.5 | 48 |
| YBL098W | 38.6 | 2.7 | 175 |
| YDR540C | 134.7 | 8.4 | 6 |
| YPR051W | 105.3 | 7 | 235 |
| YJR041C | 2.6 | 0.2 | 32 |
| YFL004W | 7 | 0.6 | 26 |
| YBL039C | 6 | 0.7 | 389 |
| YPL109C | 23.7 | 1.7 | 110 |
| YPL083C | 11.5 | 0.9 | 25 |
| YIL008W | 115.4 | 10.8 | 86 |
| YLR200W | 82 | 9.5 | 112 |
| YLR015W | 1.7 | 0.3 | 33 |
| YHR132C | 30.8 | 2.3 | 11 |
| YFR017C | 3 | 0.8 | -249 |
| YBR205W | 47.5 | 3.2 | 17 |
| YER047C | 1.5 | 0.3 | 34 |
| YLR064W | 16.5 | 0.5 | 9 |
| YLR022C | 42.9 | 5.8 | 21 |
| YJL044C | 13.5 | 1.5 | 43 |
| YFR024C-A | 1.6 | 0.3 | 17 |
| YEL018W | 3.4 | 0.4 | 81 |
| YNL242W | 1.4 | 0.2 | 574 |
| YGL053W | 16.2 | 4.3 | 79 |
| YDR019C | 4.4 | 0.3 | 95 |
| YKL197C | 1 | 0.2 | 57 |
| YKL126W | 1.3 | 0.2 | 30 |
| YML021C | 4.3 | 0.7 | 36 |
| YGL131C | 1.3 | 0.2 | 158 |
| YMR227C | 4.3 | 0.8 | 55 |
| YFL021W | 1.3 | 0.3 | 21 |
| YDR138W | 1.9 | 0.5 | 27 |
| YHR110W | 34.3 | 3.5 | 48 |
| YNL003C | 6.5 | 0.5 | 29 |
| YOL065C | 4.7 | 0.4 | 14 |
| YLR450W | 3.3 | 0.4 | 93 |
| YNL100W | 38.3 | 3.2 | 126 |
| YHR152W | 12.5 | 3.7 | 14 |
| YKR057W | 45.9 | 10 | -167 |
| YBL003C | 97.5 | 7.6 | 167 |
| YGL125W | 2.5 | 0.3 | 54 |
| YIL144W | 2.8 | 0.5 | 39 |
| YPR068C | 6.5 | 0.6 | 6 |
| YMR297W | 4.1 | 0.3 | 13 |
| YJR140C | 2.6 | 0.2 | 21 |
| YOR352W | 1.9 | 0.3 | 18 |
| YLR187W | 0.8 | 0.2 | 14 |
| YNL025C | 8.8 | 1 | 240 |
| YDR189W | 3 | 0.5 | 32 |
| YIL040W | 265.3 | 8.3 | 339 |
| YMR273C | 0.6 | 0.2 | 24 |
| YGL225W | 65.2 | 3 | 29 |
| YMR184W | 30.3 | 3.8 | 22 |
| YHR001W-A | 4053.8 | 128 | 2 |
| YGR100W | 5.8 | 0.4 | 106 |
| YDR254W | 1.4 | 0.2 | 35 |
| YOR113W | 0.5 | 0.2 | 6 |
| YGL078C | 5.5 | 0.5 | 33 |
| YAL010C | 3 | 0.4 | 43 |
| YOL072W | 4.3 | 0.3 | 66 |
| YKL067W | 316.9 | 32.9 | -1033 |
| YHR070W | 15.3 | 1.8 | 76 |
| YFL024C | 1.2 | 0.2 | 85 |
| YDR416W | 4 | 0.2 | 26 |
| YBL029W | 2.6 | 0.7 | 31 |
| YCL040W | 5.3 | 0.5 | 142 |
| YJL172W | 3.5 | 0.3 | 9 |
| YLR021W | 10.2 | 1 | 29 |
| YGR002C | 2.2 | 0.6 | 48 |
| YDR294C | 3.1 | 0.3 | 32 |
| YOR217W | 5.1 | 0.6 | 44 |
| YJL042W | 0.8 | 0.1 | 125 |
| YGL031C | 90.9 | 10.3 | 113 |
| YOR303W | 5.1 | 0.5 | 25 |
| YHR069C | 1.2 | 0.3 | 74 |
| YJR111C | 5.8 | 0.5 | 69 |
| YJR069C | 9.8 | 0.8 | 51 |
| YKL078W | 5.8 | 1.4 | 46 |
| YGL021W | 2.4 | 0.3 | 184 |
| YKL020C | 1.1 | 0.1 | 34 |
| YLR277C | 7.1 | 0.7 | 34 |
| YIR009W | 10.5 | 1.1 | 3 |
| YGL134W | 2.3 | 0.3 | 10 |
| YNL041C | 5.2 | 0.5 | 101 |
| YHR024C | 2 | 0.3 | 53 |
| YNL215W | 1.1 | 0.5 | 281 |
| YFR001W | 54.7 | 9.1 | -548 |
| YDR091C | 3 | 0.5 | 38 |
| YPL208W | 4.6 | 0.3 | 29 |
| YBR055C | 3.9 | 0.4 | 2 |
| YOL013C | 4 | 0.6 | 45 |
| YMR140W | 2.2 | 0.4 | 19 |
| YDR175C | 20.5 | 2.6 | 30 |
| YIL140W | 1.6 | 0.3 | 114 |
| YDR117C | 3.9 | 0.4 | -106 |
| YGL160W | 2 | 0.4 | 95 |
| YML025C | 1.4 | 0.5 | 50 |
| YDL109C | 9.7 | 1.3 | 15 |
| YDR123C | 3.2 | 0.7 | 13 |
| YPR108W | 4.7 | 0.5 | -101 |
| YLR097C | 7.9 | 0.5 | 26 |
| YMR318C | 12.2 | 1 | 57 |
| YFL046W | 12.3 | 0.9 | 63 |
| YCL061C | 0.5 | 0.1 | 98 |
| YMR188C | 68.8 | 6.8 | 81 |
| YNL312W | 3.2 | 0.8 | 125 |
| YLR178C | 23.6 | 4.7 | 55 |
| YDL128W | 19.3 | 2.4 | 35 |
| YDR153C | 2.4 | 0.6 | 21 |
| YDR337W | 28.1 | 2.1 | 50 |
| YFL026W | 7.2 | 0.8 | 9 |
| YKL095W | 4.6 | 0.7 | 76 |
| YIL119C | 1.1 | 0.3 | 26 |
| YPL144W | 82.6 | 5.3 | -108 |
| YNL275W | 7.7 | 0.5 | 6 |
| YJL151C | 55.2 | 11.4 | 73 |
| YLR089C | 9.5 | 1.3 | 78 |
| YER057C | 53.2 | 3.3 | -193 |
| YLR427W | 1 | 0.3 | 24 |
| YGR171C | 5 | 0.3 | 69 |
| YKL205W | 3.7 | 0.2 | 25 |
| YHR088W | 17 | 2.5 | 57 |
| YJL147C | 19.6 | 1.7 | 52 |
| YJL164C | 1 | 0.3 | -171 |
| YBL034C | 0.7 | 0.1 | 100 |
| YNL290W | 10.1 | 0.8 | 54 |
| YLR098C | 5.7 | 1.1 | 65 |
| YLR211C | 6.4 | 0.7 | 22 |
| YKL046C | 2.5 | 0.2 | 40 |
| YOL121C | 7.6 | 1.1 | -76 |
| YDL042C | 5.6 | 0.6 | 21 |
| YLR381W | 12.6 | 1.1 | 26 |
| YDR390C | 2.2 | 0.4 | 47 |
| YGR150C | 3.1 | 0.3 | 23 |
| YHR043C | 4.6 | 0.6 | 277 |
| YBR096W | 118.1 | 4.8 | 32 |
| YHR052W | 2.4 | 0.8 | 199 |
| YNL068C | 0.5 | 0.1 | 26 |
| YOR320C | 9.8 | 0.6 | 3 |
| YMR224C | 1 | 0.2 | 7 |
| YOL130W | 1.8 | 0.4 | 13 |
| YLR312W-A | 2.1 | 0.2 | 453 |
| YKR089C | 1.7 | 0.3 | 17 |
| YGR180C | 37.4 | 2.9 | 372 |
| YOR281C | 5.6 | 0.8 | 203 |
| YBR299W | 2.9 | 0.4 | 42 |
| YGR161C | 4.3 | 0.9 | 57 |
| YML042W | 1.5 | 0.2 | 47 |
| YHR016C | 3.1 | 0.7 | 8 |
| YPL170W | 2.4 | 0.5 | -470 |
| YPL222W | 5.3 | 0.4 | 25 |
| YMR123W | 30.8 | 1.1 | 57 |
| YPL086C | 3.7 | 0.3 | 32 |
| YOR220W | 12.3 | 2.4 | 25 |
| YDR493W | 69.2 | 9.5 | 210 |
| YER175C | 49.8 | 6.6 | -187 |
| YPL072W | 6.2 | 0.6 | 53 |
| YIL143C | 2.4 | 0.4 | 2 |
| YBR171W | 4.3 | 0.5 | -3619 |
| YHR116W | 1.9 | 0.9 | 3 |
| YDR069C | 1.1 | 0.2 | 69 |
| YOR198C | 4.3 | 0.7 | 128 |
| YNL059C | 4.2 | 0.4 | 38 |
| YPL249C | 1.2 | 0.2 | -175 |
| YLR026C | 1.7 | 0.4 | 49 |
| YBL086C | 1.1 | 0.2 | 32 |
| YOR160W | 5.3 | 0.3 | 68 |
| YPL271W | 5.1 | 1.2 | -176 |
| YKL092C | 1.1 | 0.1 | 38 |
| YML048W | 3.6 | 0.7 | 108 |
| YHR204W | 3.1 | 0.4 | 27 |
| YER099C | 9.4 | 0.8 | -5160 |
| YDR419W | 5.5 | 0.7 | 61 |
| YKL021C | 9 | 0.8 | 171 |
| YGL070C | 127.2 | 9.5 | 87 |
| YGR187C | 19.8 | 2.7 | 47 |
| YOL067C | 2.2 | 0.9 | 32 |
| YJR088C | 18.9 | 0.8 | -630 |
| YOR236W | 32.7 | 3.9 | 36 |
| YOR081C | 2.9 | 0.4 | 17 |
| YML035C | 2.5 | 0.6 | 33 |
| YMR154C | 5.8 | 0.5 | 2 |
| YOR363C | 3.3 | 0.4 | 3 |
| YGL206C | 2.1 | 0.1 | 102 |
| YMR083W | 18.2 | 1 | -431 |
| YPR131C | 50.4 | 4.4 | 140 |
| YJR046W | 3.9 | 0.6 | 15 |
| YDR506C | 3 | 0.3 | 10 |
| YGL100W | 5.4 | 0.8 | 72 |
| YPR115W | 0.6 | 0.1 | 3 |
| YLR131C | 0.6 | 0.1 | 3 |
| YKR078W | 7.3 | 0.9 | -1826 |
| YNL161W | 0.5 | 0.2 | 31 |
| YJL191W | 45.4 | 8 | -327 |
| YPL150W | 1.2 | 0.2 | 9 |
| YDL185W | 2.7 | 0.3 | 109 |
| YGR130C | 2.1 | 0.5 | 44 |
| YDL166C | 57.6 | 4.1 | 16513 |
| YOL041C | 3.6 | 0.9 | 35 |
| YGR186W | 0.4 | 0.1 | 290 |
| YML123C | 4.4 | 0.7 | 25 |
| YCR039C | 33.5 | 4.5 | 107 |
| YLR405W | 13.4 | 0.9 | 116 |
| YBR222C | 9.4 | 0.7 | 27 |
| YNR043W | 4.3 | 0.8 | 65 |
| YJR010C-A | 726.1 | 47.8 | 135 |
| YFR002W | 3.2 | 0.2 | 35 |
| YMR163C | 4.9 | 0.5 | 10 |
| YKL195W | 0.8 | 0.4 | 22 |
| YDL035C | 0.6 | 0.2 | 31 |
| YPL135W | 72.3 | 8.2 | 25 |
| YKL106W | 3.6 | 0.3 | 42 |
| YER104W | 6.4 | 1.9 | 3 |
| YHL004W | 9.9 | 1.7 | 116 |
| YGL130W | 2.4 | 0.3 | 3 |
| YCR084C | 1.5 | 0.4 | 27 |
| YPL221W | 2.1 | 0.4 | -338 |
| YGL108C | 37.9 | 12.3 | -102 |
| YLR028C | 6.3 | 0.4 | 59 |
| YLR356W | 19.7 | 5.6 | 15 |
| YOR341W | 2 | 0.3 | 27 |
| YGL086W | 1.6 | 0.3 | 24 |
| YDR346C | 2 | 0.5 | 55 |
| YER070W | 2.1 | 0.2 | 77 |
| YJL057C | 4.1 | 0.5 | 21 |
| YBL046W | 4.5 | 1.1 | 65 |
| YHR178W | 0.5 | 0.2 | 37 |
| YBR151W | 25.1 | 2.4 | 55 |
| YIL125W | 13.8 | 1.4 | 85 |
| YAL027W | 3.8 | 0.5 | 36 |
| YGR191W | 6 | 0.4 | 23 |
| YIL147C | 1.1 | 0.2 | 34 |
| YHR010W | 113.3 | 7.9 | 45 |
| YDL106C | 0.8 | 0.3 | 48 |
| YML107C | 3.5 | 0.3 | 182 |
| YKR006C | 10.3 | 0.9 | 68 |
| YKR024C | 4.8 | 0.6 | 48 |
| YFL031W | 14 | 2 | 12 |
| YOR358W | 1.7 | 1 | -100 |
| YBL054W | 1.5 | 0.5 | 18 |
| YIL044C | 4.8 | 1.1 | 51 |
| YCR018C | 4 | 0.8 | 2 |
| YJL003W | 199.9 | 27.6 | 2 |
| YAL017W | 0.9 | 0.2 | 3 |
| YDL015C | 44.8 | 3.1 | 59 |
| YOR371C | 2 | 0.4 | 28 |
| YNL139C | 0.9 | 0.1 | 86 |
| YNL264C | 3.1 | 0.3 | 52 |
| YNL037C | 38.2 | 3.3 | 29 |
| YHR038W | 30.2 | 3.2 | 38 |
| YLR264W | 236 | 28.7 | 110 |
| YFR020W | 2.1 | 0.4 | 49 |
| YJL013C | 3.5 | 0.4 | 30 |
| YMR067C | 4.8 | 0.7 | 22 |
| YHR089C | 41.5 | 7.9 | 30 |
| YKL072W | 2.7 | 0.3 | 25 |
| YGL124C | 3.2 | 0.3 | 37 |
| YMR198W | 7.3 | 1.2 | 18 |
| YDL178W | 3.5 | 0.3 | 38 |
| YHL017W | 4.2 | 0.4 | 648 |
| YBR255W | 0.5 | 0.2 | 3 |
| YNL304W | 5.1 | 0.6 | 24 |
| YER001W | 2.1 | 0.3 | 215 |
| YGR028W | 14.7 | 0.9 | 36 |
| YKL105C | 0.7 | 0.2 | 32 |
| YIL085C | 17.6 | 1.5 | 55 |
| YFR053C | 11.2 | 0.8 | 49 |
| YMR138W | 54.5 | 4.9 | 64 |
| YOR091W | 6.5 | 0.5 | 45 |
| YDR299W | 1.9 | 0.4 | 20 |
| YER054C | 0.6 | 0.1 | 40 |
| YDR236C | 68.6 | 4.2 | -220 |
| YPR091C | 1.3 | 0.3 | 51 |
| YMR215W | 6.3 | 0.5 | 17 |
| YGR246C | 5.3 | 0.7 | 26 |
| YBL082C | 48.4 | 2.4 | 30 |
| YBR149W | 6.6 | 0.5 | 96 |
| YJR079W | 203.9 | 9.9 | 44 |
| YBL091C | 2.4 | 0.3 | 36 |
| YER011W | 3.9 | 0.6 | 2 |
| YHR055C | 127.3 | 6.6 | 189 |
| YDL001W | 11.1 | 2.6 | 2 |
| YBL001C |  |  | 124 |
| YHR097C | 1.5 | 0.6 | 21 |
| YPR176C | 7.4 | 0.6 | -65 |
| YER036C | 2.9 | 0.2 | 53 |
| YBL029C-A | 92.3 | 9.6 | 12 |
| YLR218C | 21.3 | 1.1 | 437 |
| YFL010C | 19.6 | 6.4 | 92 |
| YJR031C | 1.9 | 0.2 | -137 |
| YDR083W | 2.9 | 0.9 | 81 |
| YDR198C | 26.5 | 1.5 | 28 |
| YGR134W | 2 | 0.2 | 10 |
| YAR031W | 43.4 | 7 | 104 |
| YLR082C | 2.9 | 0.7 | 67 |
| YHR169W | 18.4 | 3.7 | -186 |
| YGR232W | 13.8 | 1 | -112 |
| YGR036C | 18.6 | 3.1 | -110 |
| YJL162C | 1.3 | 0.4 | 20 |
| YDR511W | 8.9 | 1 | 27 |
| YLR084C | 1.1 | 0.1 | 55 |
| YER050C | 29.3 | 6.2 | 66 |
| YPL139C | 9.6 | 0.7 | -123 |
| YCL005W | 10.7 | 0.9 | 42 |
| YMR148W | 113.5 | 19.1 | -121 |
| YER013W | 1.7 | 0.2 | 45 |
| YDR388W | 4 | 0.5 | 41 |
| YEL042W | 4.6 | 0.4 | 2 |
| YMR299C | 16.7 | 1.8 | 161 |
| YDL040C | 2.1 | 0.2 | 65 |
| YGR058W | 1.7 | 0.3 | 16 |
| YNL212W | 4.8 | 0.6 | 22 |
| YDR338C | 3.9 | 0.8 | 18 |
| YOR276W | 23.6 | 5.1 | -320 |
| YPR100W | 6.9 | 0.5 | -390 |
| YPL260W | 1 | 0.2 | 57 |
| YOL044W | 9.6 | 0.8 | 143 |
| YJR093C | 4.3 | 1.6 | 72 |
| YML105C | 4 | 0.8 | 66 |
| YKR071C | 7.8 | 0.5 | 59 |
| YER023W | 3.8 | 0.4 | -337 |
| YKL181W | 2.2 | 0.4 | 44 |
| YLR129W | 1.7 | 0.2 | 41 |
| YLL040C | 0.8 | 0.1 | -203 |
| YOR332W | 35.1 | 3.2 | 10 |
| YNL197C | 0.8 | 0.2 | 22 |
| YOR253W | 12.2 | 1 | 44 |
| YDR200C | 0.6 | 0.1 | 29 |
| YGL228W | 8.4 | 0.6 | 230 |
| YOR350C | 6.8 | 0.6 | 31 |
| YNL113W | 311.5 | 18.9 | -302 |
| YLR093C | 1 | 0.3 | 46 |
| YLR369W | 1.9 | 0.2 | 61 |
| YOL055C | 5.4 | 0.4 | 50 |
| YKR065C | 96.4 | 7.9 | 45 |
| YOR017W | 2 | 0.4 | 19 |
| YDL059C | 3.6 | 0.5 | 23 |
| YJR156C | 117.9 | 7.3 | 3 |
| YCR012W | 2.9 | 0.4 | 199 |
| YOR176W | 7.4 | 0.8 | 123 |
| YKL094W | 6.9 | 0.5 | 88 |
| YOR138C | 0.8 | 0.2 | 30 |
| YGR104C | 5.1 | 0.6 | 124 |
| YBL079W | 0.8 | 0.1 | 20 |
| YGR120C | 6.3 | 0.9 | 158 |
| YGL173C | 0.6 | 0.1 | 81 |
| YPL190C | 0.4 | 0.2 | 38 |
| YDL205C | 95.9 | 5.4 | -61 |
| YGR270W | 1 | 0.1 | 893 |
| YLR447C | 8.8 | 0.5 | 1348 |
| YLR208W | 2.4 | 0.4 | -282 |
| YCL035C | 4.7 | 0.6 | -296 |
| YJR044C | 146.2 | 7.8 | -175 |
| YER087C-A | 8.7 | 0.6 | 55 |
| YNL002C | 6.8 | 0.8 | 96 |
| YFR040W | 0.5 | 0.1 | 42 |
| YIL077C | 1.2 | 0.3 | 62 |
| YBL084C | 1.1 | 0.3 | 3 |
| YMR104C | 1.5 | 0.3 | 71 |
| YJR142W | 15.6 | 0.9 | 35 |
| YKL179C | 1.8 | 0.4 | 23 |
| YER139C | 84.5 | 8.5 | 92 |
| YNL132W | 4.5 | 0.4 | 31 |
| YOL060C | 1.4 | 0.3 | 8 |
| YAR028W | 5.2 | 0.9 | 62 |
| YKL168C | 1.5 | 0.3 | 51 |
| YFR014C | 3.2 | 0.9 | 111 |
| YJR134C | 3.4 | 0.5 | 1950 |
| YPL231W | 0.8 | 0.1 | 19 |
| YOR386W | 10.6 | 0.7 | 28 |
| YDR452W | 11.6 | 1.5 | 9 |
| YOR058C | 1.7 | 0.2 | 3 |
| YBL099W | 5 | 0.4 | 49 |
| YLR061W | 8.8 | 1.1 | 207 |
| YBR176W | 13.2 | 1 | 118 |
| YPL160W | 3.8 | 0.3 | 81 |
| YJL197W | 1.1 | 0.2 | -4068 |
| YDL175C | 5.2 | 1.3 | 30 |
| YDR365C | 5.1 | 1.3 | 2 |
| YBR001C | 2.8 | 0.3 | 29 |
| YNL181W | 2.7 | 0.5 | 2991 |
| YPR041W | 5.1 | 0.5 | 186 |
| YML016C | 1.1 | 0.4 | 50 |
| YDR051C | 3.8 | 0.5 | 46 |
| YDR309C | 1.3 | 0.4 | 13 |
| YDR108W | 1.9 | 0.2 | 42 |
| YFL002C | 16.1 | 1.6 | 29 |
| YOL100W | 1.2 | 0.2 | 12 |
| YLR455W | 7.7 | 1.7 | 104 |
| YKL061W | 68.3 | 5.2 | 54 |
| YGR013W | 2.1 | 0.6 | 73 |
| YDL033C | 7.9 | 0.8 | 44 |
| YEL055C | 2.5 | 0.4 | 32 |
| YBL017C | 1.3 | 0.1 | 30 |
| YJR012C | 7.6 | 2.7 | 419 |
| YDR449C | 11.3 | 0.7 | 34 |
| YDL007W | 26.9 | 3.6 | 17 |
| YER067W | 72 | 11 | -296 |
| YGL254W | 3.4 | 0.8 | 84 |
| YGR106C | 27.1 | 4.4 | 39 |
| YIR011C | 5.6 | 0.8 | 3 |
| YMR161W | 16.7 | 2.8 | 56 |
| YDL144C | 8.1 | 0.5 | 145 |
| YLR351C | 8 | 0.9 | -814 |
| YGR108W | 7.3 | 0.7 | 14 |
| YBR009C | 1452.5 | 318 | 62 |
| YKR070W | 4 | 0.4 | 71 |
| YJR161C | 8.4 | 0.8 | 23 |
| YMR200W | 7.2 | 0.9 | 20 |
| YAR002W | 3 | 0.6 | 15 |
| YIL104C | 2.8 | 0.4 | 37 |
| YLR457C | 17.4 | 3.7 | 31 |
| YAL023C | 7.6 | 0.6 | 61 |
| YJR076C | 3.1 | 0.8 | 62 |
| YJR082C | 9.1 | 3 | 184 |
| YBL090W | 15.4 | 2.7 | 66 |
| YEL044W | 5.4 | 1.8 | -77 |
| YML017W | 0.4 | 0.1 | 20 |
| YNL001W | 14.9 | 1 | 106 |
| YHR174W | 14.3 | 0.9 | 27 |
| YCL024W | 1.5 | 0.2 | 22 |
| YBL008W | 1.3 | 0.1 | 49 |
| YOR006C | 0.4 | 0.2 | 58 |
| YER089C | 3.3 | 0.5 | 100 |
| YHL027W | 1 | 0.2 | 2 |
| YKL029C | 11.9 | 1.4 | 29 |
| YBR114W | 4.7 | 0.5 | 33 |
| YOR285W | 6 | 1.1 | 140 |
| YGL094C | 1.5 | 0.2 | 23 |
| YHR112C | 68.1 | 4.8 | -73 |
| YNL254C | 20.1 | 2.2 | 14 |
| YBR252W | 12.4 | 1.1 | -84 |
| YPL265W | 5.2 | 0.7 | 7 |
| YCR095C | 2.2 | 0.4 | 104 |
| YKR023W | 1.3 | 0.3 | 31 |
| YGR222W | 29.5 | 2.4 | 89 |
| YLR238W | 1.7 | 0.2 | 2 |
| YCL064C | 7.3 | 0.5 | 70 |
| YBL058W | 1.6 | 0.4 | 62 |
| YBR253W | 32.3 | 4.8 | 176 |
| YDR213W | 1.3 | 0.2 | 33 |
| YBL018C | 107.2 | 6.1 | 45 |
| YPR132W | 10.1 | 0.9 | 43 |
| YGL140C | 1.4 | 0.2 | 22 |
| YLR392C | 1.2 | 0.1 | -218 |
| YMR228W | 64.6 | 3.5 | 36 |
| YGR163W | 7.4 | 0.5 | 37 |
| YER010C | 3.8 | 0.4 | 21 |
| YPR008W | 0.6 | 0.2 | 17 |
| YOR226C | 168.4 | 14.2 | 31 |
| YML081W | 1.1 | 0.2 | 3 |
| YPR031W | 7 | 1 | 12 |
| YKL024C | 158.3 | 9.6 | 329 |
| YLR281C | 41.8 | 8.9 | 115 |
| YDR142C | 3.8 | 0.4 | 174 |
| YLR412W | 20.2 | 2.7 | -224 |
| YLR376C | 15.3 | 0.9 | 39 |
| YGR159C | 9.2 | 2.7 | -258 |
| YCL028W | 0.4 | 0.2 | 51 |
| YDR181C | 3 | 0.4 | 21 |
| YPL116W | 1.9 | 0.6 | -32558 |
| YDR310C | 0.6 | 0.2 | 12 |
| YOR131C | 7.9 | 0.5 | 23 |
| YGL153W | 1.2 | 0.3 | 66 |
| YPR134W | 2.7 | 0.4 | 34 |
| YHL008C | 2.5 | 0.4 | 134 |
| YER092W | 45.8 | 6.4 | 75 |
| YDR174W | 1.8 | 0.5 | -472 |
| YDL192W | 33.1 | 3.4 | -366 |
| YNR012W | 24.5 | 1.5 | 50 |
| YDR230W | 23.7 | 7.1 | 3 |
| YIL053W | 4 | 0.9 | 113 |
| YHR197W | 4.4 | 0.4 | 80 |
| YER102W | 46 | 6.3 | 98 |
| YLR036C | 9.1 | 0.6 | 58 |
| YBR286W | 4.7 | 0.4 | 9 |
| YOR315W | 13.7 | 1.9 | 26 |
| YLL039C | 291.6 | 28.2 | 13 |
| YLR315W | 27.3 | 5.9 | -294 |
| YPR029C | 2.4 | 0.2 | 27 |
| YGL016W | 4.8 | 0.3 | 80 |
| YPL207W | 0.8 | 0.2 | 164 |
| YLR032W | 2.2 | 0.4 | 84 |
| YDR110W | 1.6 | 0.3 | 12 |
| YJL161W | 44.5 | 4.2 | 93 |
| YNL066W | 2.6 | 0.3 | 57 |
| YDR324C | 2.4 | 0.2 | 46 |
| YBR168W | 10.6 | 2 | 49 |
| YML103C | 1.4 | 0.2 | 46 |
| YHR051W | 123.2 | 7.8 | 48 |
| YOR056C | 5.1 | 0.7 | 234 |
| YKR087C | 3.9 | 0.5 | 70 |
| YML079W | 3.1 | 0.4 | 76 |
| YJL081C | 5.3 | 0.6 | 86 |
| YGR067C | 8.1 | 0.5 | -250 |
| YPR097W | 1.9 | 0.3 | 19 |
| YGL038C | 11.2 | 1.7 | 6 |
| YGR086C | 24.6 | 6.8 | 113 |
| YDR034C | 0.8 | 0.2 | 48 |
| YOL077C | 11.3 | 0.9 | 170 |
| YHR006W | 4.1 | 0.6 | 17 |
| YIL062C | 357.5 | 35.2 | 32 |
| YKL075C | 4 | 0.7 | 46 |
| YOL017W | 1.6 | 0.3 | 30 |
| YKL006C-A | 14 | 3.4 | 16 |
| YJR072C | 1.9 | 0.3 | 141 |
| YJR097W | 39.1 | 5.2 | 41 |
| YHR061C | 1 | 0.3 | 2 |
| YKR035W-A | 5.9 | 0.9 | 100 |
| YIL095W | 0.6 | 0.2 | 31 |
| YOL030W | 3.5 | 0.4 | 11 |
| YOR327C | 20.1 | 5.3 | 10 |
| YHR106W | 5.2 | 0.5 | 38 |
| YMR240C | 1.9 | 0.7 | 54 |
| YDL213C | 2.6 | 0.8 | -796 |
| YNL184C | 35.9 | 12.9 | 95 |
| YEL060C | 1.1 | 0.3 | 3 |
| YDR115W | 69.2 | 11.8 | 93 |
| YAR071W | 18.1 | 1.3 | 10 |
| YMR296C | 14.6 | 1.2 | -167 |
| YER123W | 1.3 | 0.3 | 25 |
| YDR165W | 10.4 | 1.4 | 26 |
| YDR043C | 2.7 | 0.4 | 10 |
| YDR166C | 1.9 | 0.2 | 28 |
| YBR086C | 1 | 0.2 | 482 |
| YLR272C | 2.1 | 0.3 | -2499 |
| YPL250C | 9.7 | 1 | -197 |
| YDR076W | 14.8 | 1.8 | 14 |
| YIR001C | 4.2 | 0.8 | 609 |
| YBL108W | 372.5 | 17.1 | 8 |
| YEL041W | 3.2 | 0.7 | 70 |
| YLR360W | 7.3 | 0.4 | 40 |
| YDL092W | 5.6 | 1 | -272 |
| YGR174C | 5.5 | 1.2 | 759 |
| YGL154C | 19.2 | 0.9 | 94 |
| YDL140C | 1.4 | 0.2 | 22 |
| YDR012W | 9.2 | 0.9 | -62 |
| YNL143C | 33.1 | 8.1 | 37 |
| YHR196W | 2.1 | 0.2 | 36 |
| YDR289C | 6.5 | 1.6 | 86 |
| YOL048C | 37 | 3.7 | 112 |
| YCR003W | 5.9 | 1 | 225 |
| YGR016W | 45.2 | 3 | 9 |
| YDR530C | 2.7 | 0.4 | 36 |
| YML080W | 5.1 | 0.5 | 37 |
| YML041C | 11.7 | 2.5 | 75 |
| YGR105W | 84.4 | 16.8 | 84 |
| YBR062C | 26 | 6 | 8 |
| YMR202W | 53.3 | 4.2 | 29 |
| YGR201C | 115.1 | 7.3 | 120 |
| YLR060W | 4.5 | 0.4 | 44 |
| YLR039C | 1.9 | 0.2 | 54 |
| YCR052W | 0.8 | 0.3 | 34 |
| YOR286W | 54.1 | 7.9 | -405 |
| YGL148W | 5.1 | 0.9 | 69 |
| YPR140W | 13 | 0.8 | 26 |
| YGL213C | 6.3 | 0.7 | 258 |
| YDR214W | 3.3 | 0.8 | 34 |
| YGR119C | 1.3 | 0.4 | 14 |
| YIL124W | 11.7 | 0.8 | 76 |
| YMR192W | 2.9 | 0.4 | 19 |
| YHR206W | 1.2 | 0.2 | 23 |
| YHR084W | 1.4 | 0.4 | 34 |
| YMR112C | 28.3 | 4.8 | 116 |
| YDR049W | 4.5 | 1 | 37 |
| YOL109W | 52.7 | 10.8 | -674 |
| YDR256C | 1.9 | 0.3 | 31 |
| YJR048W | 126.7 | 11.2 | 61 |
| YCR036W | 21.3 | 2.6 | 61 |
| YNL135C | 8.1 | 1.1 | 196 |
| YCR044C | 71.8 | 4 | -77 |
| YAL047C | 4 | 0.6 | -191 |
| YNR035C | 8.2 | 1 | 43 |
| YBL076C | 3.9 | 0.4 | 88 |
| YCR024C | 8.6 | 0.7 | 27 |
| YBR234C | 4 | 0.3 | 66 |
| YDR252W | 3.9 | 0.5 | 262 |
| YOR229W | 1 | 0.2 | 52 |
| YML007W | 0.8 | 0.3 | 23 |
| YBR024W | 5.1 | 0.5 | 13 |
| YEL007W | 0.7 | 0.2 | 8 |
| YNL291C | 4.7 | 0.4 | 10 |
| YNL259C | 1002.8 | 47.8 | 39 |
| YKL091C | 36.5 | 3.6 | 170 |
| YDR140W | 50.7 | 3.5 | 239 |
| YNL251C | 1.4 | 0.6 | 53 |
| YML100W | 1.4 | 0.2 | 39 |
| YNL279W | 2.7 | 0.3 | 2 |
| YJL006C | 7.4 | 0.8 | 28 |
| YPL048W | 5 | 0.5 | 45 |
| YDL234C | 3.2 | 0.3 | 77 |
| YLR370C | 34.8 | 2.7 | 51 |
| YER158C | 0.9 | 0.3 | 3 |
| YFR037C | 3.1 | 0.5 | 74 |
| YHR144C | 8.4 | 0.5 | 41 |
| YIL161W | 1.1 | 0.5 | 62 |
| YGR047C | 5.1 | 0.5 | 23 |
| YPR075C | 11.9 | 3.2 | 64 |
| YLR190W | 2.7 | 0.5 | 26 |
| YNL031C | 4070.8 | 128 | 223 |
| YGR282C | 32.6 | 2.6 | 27 |
| YPR138C | 3.9 | 0.8 | 1421 |
| YDR191W | 7.4 | 0.9 | 33 |
| YHR135C | 2.4 | 0.3 | 14 |
| YNL315C | 6.1 | 0.8 | 64 |
| YPL045W | 5.1 | 0.3 | 30 |
| YBR071W | 9.1 | 3.3 | 3 |
| YKR060W | 27.2 | 3.1 | 26 |
| YOR317W | 2.7 | 0.2 | -237 |
| YOL062C | 3.4 | 0.3 | 2 |
| YDR495C | 5 | 0.7 | 54 |
| YKL025C | 2.7 | 0.2 | 36 |
| YJL074C | 1.3 | 0.2 | 217 |
| YLR017W | 10.6 | 0.8 | 26 |
| YJL180C | 7.9 | 0.7 | 178 |
| YLR304C | 2.7 | 0.3 | 72 |
| YGL017W | 3.6 | 0.5 | 443 |
| YGR237C | 1.8 | 0.3 | 41 |
| YDR410C | 19.6 | 1.1 | 249 |
| YER141W | 67.4 | 4.6 | 50 |
| YMR214W | 3.3 | 0.5 | 67 |
| YOR144C | 2.8 | 0.4 | 2 |
| YGL019W | 13.2 | 1.1 | 17 |
| YPR025C | 1.6 | 0.5 | 84 |
| YBR003W | 3.8 | 0.5 | 64 |
| YJL058C | 4.3 | 0.5 | 28 |
| YLR151C | 4.6 | 0.4 | 31 |
| YCR063W | 20.2 | 1.1 | 26 |
| YLR214W | 12.2 | 0.6 | 220 |
| YLR348C | 5.6 | 0.9 | 398 |
| YML085C | 6.6 | 0.8 | 41 |
| YPR086W | 3.1 | 0.5 | 17 |
| YGR241C | 4.7 | 1.2 | 365 |
| YPL223C | 7.7 | 3.5 | 80 |
| YMR124W | 0.3 | 0.1 | 430 |
| YJL139C | 12.2 | 0.7 | 15 |
| YDR100W | 450.1 | 24.8 | 76 |
| YNL084C | 2.2 | 0.7 | 90 |
| YBL024W | 7 | 0.6 | 36 |
| YKL209C | 3.1 | 0.2 | 186 |
| YML004C | 1.6 | 0.3 | 85 |
| YKL040C | 24.5 | 2.8 | 117 |
| YLR127C | 1.7 | 0.2 | 30 |
| YBL095W | 6.9 | 0.9 | 46 |
| YGL202W | 26.2 | 2.8 | 59 |
| YGR179C | 2.2 | 0.5 | 30 |
| YGR250C | 0.8 | 0.1 | 24 |
| YLR289W | 7 | 0.5 | -82 |
| YGR263C | 14.1 | 1.8 | 26 |
| YOR181W | 0.8 | 0.3 | 20 |
| YNL191W | 6.6 | 0.5 | 53 |
| YNL110C | 51.2 | 6 | 155 |
| YMR107W | 14.6 | 4.9 | 27 |
| YOR127W | 1 | 0.2 | 37 |
| YPL234C | 38.6 | 1.2 | 75 |
| YAL060W | 7 | 0.9 | 68 |
| YBR089C-A | 26.6 | 8.9 | 103 |
| YNL123W | 2.8 | 0.2 | 72 |
| YBR233W-A | 20 | 12 | 228 |
| YOR208W | 1 | 0.2 | 3 |
| YEL029C | 61.1 | 3 | 17 |
| YIL145C | 10.4 | 0.9 | -104 |
| YHR098C | 1.1 | 0.1 | 15 |
| YIL074C | 4.7 | 0.7 | 350 |
| YBR247C | 6.5 | 0.8 | 35 |
| YOR163W | 11.1 | 4.1 | -132 |
| YPL166W | 5.5 | 0.9 | 48 |
| YLR033W | 1.1 | 0.2 | 46 |
| YCL032W | 2.1 | 0.5 | 39 |
| YDR456W | 3.9 | 0.4 | 147 |
| YJR104C | 3 | 0.6 | -404 |
| YDR517W | 20.2 | 3.5 | 61 |
| YCR005C | 12.3 | 0.8 | 30 |
| YNL103W | 0.3 | 0.1 | 9 |
| YDL182W | 11.3 | 0.8 | 186 |
| YGR255C | 8 | 0.7 | 184 |
| YPR033C | 7.9 | 0.8 | 45 |
| YKR075C | 9.4 | 1.6 | 8 |
| YKL192C | 104.8 | 8.7 | -3127 |
| YOR207C | 2 | 0.2 | 25 |
| YGL083W | 7.1 | 1.1 | 36 |
| YKR077W | 2.1 | 0.4 | 35 |
| YDL134C | 8.6 | 1.5 | 45 |
| YMR020W | 4.4 | 0.4 | 54 |
| YML104C | 1.4 | 0.2 | 3 |
| YKR096W | 0.8 | 0.1 | 39 |
| YGR143W | 1.2 | 0.2 | 3 |
| YMR181C | 15.8 | 2.9 | 26 |
| YER129W | 0.7 | 0.2 | 240 |
| YER032W | 1.1 | 0.3 | 3 |
| YAR019C | 3.9 | 0.4 | 109 |
| YDR441C | 2676.6 | 168 | 28 |
| YDR089W | 1.8 | 0.3 | 3 |
| YLR299W | 6 | 0.4 | 18 |
| YIL055C | 0.6 | 0.2 | 16 |
| YJL096W | 79.3 | 9.6 | -67 |
| YOL142W | 15 | 0.9 | 242 |
| YOR262W | 5.1 | 0.4 | -370 |
| YDR364C | 2.9 | 0.8 | 38 |
| YMR058W | 2 | 0.2 | 46 |
| YBR175W | 3.3 | 0.2 | 1181 |
| YMR132C | 10 | 0.9 | 38 |
| YGL145W | 1.3 | 0.2 | 40 |
| YFR031C-A | 13.9 | 4.2 | 257 |
| YPR113W | 15 | 2.6 | 17 |
| YMR060C | 11.5 | 0.8 | 21 |
| YMR257C | 5.3 | 0.5 | 26 |
| YKL145W | 2.7 | 0.8 | 9 |
| YML081C-A | 111.6 | 18.4 | 63 |
| YGR142W | 3.7 | 0.6 | 13 |
| YGR077C | 25.6 | 1.3 | 26 |
| YOL148C | 0.7 | 0.2 | 25 |
| YMR043W | 1.9 | 0.7 | -991 |
| YDR439W | 4.5 | 1.3 | 38 |
| YPL140C | 5.1 | 0.7 | 29 |
| YMR066W | 3.9 | 0.3 | 38 |
| YBL067C | 1.3 | 0.3 | 32 |
| YDR300C | 5.2 | 0.8 | -654 |
| YBL059W | 27.9 | 4.4 | 71 |
| YCR069W | 10.5 | 0.9 | 41 |
| YDL111C | 3.5 | 0.4 | -489 |
| YDR477W | 3 | 0.6 | 34 |
| YDR034W-B | 79.5 | 41.4 | -83 |
| YPL269W | 5.9 | 1 | 30 |
| YJR067C | 35 | 4.9 | -385 |
| YLL060C | 1.9 | 0.6 | 24 |
| YIL061C | 12.5 | 3.1 | 49 |
| YEL048C | 164.6 | 11.4 | 607 |
| YJR016C | 3.8 | 0.5 | 45 |
| YOR106W | 9.7 | 0.9 | 13 |
| YNL189W | 1.4 | 0.7 | 39 |
| YDR125C | 15.6 | 1.3 | 33 |
| YMR075W | 1.2 | 0.2 | 37 |
| YEL066W | 3.1 | 0.9 | 3 |
| YPL093W | 2.9 | 0.4 | 28 |
| YLR113W | 3.5 | 0.9 | 273 |
| YPL028W | 4 | 0.5 | 117 |
| YKR072C | 2.2 | 0.5 | 38 |
| YER091C | 3.2 | 0.2 | 38 |
| YIL092W | 1.6 | 0.4 | 3 |
| YDR297W | 28.8 | 3.1 | -129 |
| YOL033W | 5.5 | 0.3 | 108 |
| YAL035W | 4.1 | 0.4 | 321 |
| YDL189W | 0.9 | 0.3 | 25 |
| YJL166W | 33.5 | 1 | 7 |
| YER095W | 0.8 | 0.2 | 60 |
| YCL025C | 4.3 | 0.3 | 19 |
| YBR245C | 0.9 | 0.1 | 37 |
| YGL157W | 12.5 | 0.8 | 56 |
| YDR519W | 10302.6 | 318 | -1164 |
| YNL047C | 2.6 | 0.3 | 49 |
| YKR062W | 10.9 | 2.2 | 30 |
| YKR018C | 5.9 | 0.5 | 19 |
| YHL002W | 3.3 | 0.7 | 23 |
| YOL124C | 6.2 | 0.7 | 50 |
| YLR453C | 23.1 | 1.8 | 58 |
| YCR031C | 102.6 | 10.2 | 40 |
| YDR040C | 1.9 | 0.3 | -345 |
| YPL094C | 4.9 | 0.9 | 157 |
| YPL191C | 2.4 | 0.3 | -537 |
| YOR159C | 53.8 | 8.3 | -80 |
| YCL052C | 9.7 | 0.7 | -225 |
| YHR062C | 3.8 | 0.5 | 46 |
| YHR013C | 3.1 | 0.9 | 244 |
| YDL056W | 1.3 | 0.2 | 20 |
| YLR274W | 1.7 | 0.3 | 43 |
| YJL063C | 55.2 | 4.7 | 105 |
| YLR364W | 8.6 | 1.1 | 16 |
| YPR105C | 5.3 | 0.4 | 35 |
| YBL068W | 7 | 0.8 | 116 |
| YIL153W | 5.4 | 0.5 | 131 |
| YPL193W | 3.8 | 1.1 | 30 |
| YIL050W | 9.3 | 0.8 | 19 |
| YNL261W | 6 | 0.9 | 34 |
| YML120C | 11.2 | 1.5 | 43 |
| YDR196C | 16.2 | 0.9 | -855 |
| YLR085C | 4.5 | 0.8 | 21 |
| YDR362C | 3.6 | 0.4 | 11 |
| YGR146C | 24.9 | 3.8 | 18 |
| YER149C | 4.3 | 0.5 | 79 |
| YPR163C | 1.9 | 0.5 | 67 |
| YGL066W | 0.3 | 0.1 | 20 |
| YGL022W | 6.8 | 0.8 | 19 |
| YML061C | 1.6 | 0.2 | 166 |
| YKR084C | 1.2 | 0.3 | 43 |
| YDR424C | 85.8 | 18.9 | 10 |
| YIL035C | 12.8 | 0.9 | 62 |
| YPL074W | 0.8 | 0.2 | 40 |
| YLR114C | 0.3 | 0.1 | 112 |
| YGR014W | 2.2 | 0.4 | 38 |
| YLR265C | 7.8 | 0.8 | 28 |
| YDR354W | 32.7 | 2.5 | 24 |
| YGL169W | 24.4 | 2.5 | 26 |
| YGR157W | 2 | 0.2 | 43 |
| YHR159W | 1.1 | 0.3 | 29 |
| YHR198C | 9.9 | 0.7 | 625 |
| YBL038W | 6.1 | 0.5 | 276 |
| YKR031C | 0.4 | 0.1 | 19 |
| YNL082W | 1.6 | 0.2 | 2 |
| YPL096W | 5 | 0.8 | 108 |
| YMR097C | 8.3 | 0.7 | 69 |
| YNR075W | 13.7 | 0.9 | 548 |
| YGL150C | 0.3 | 0.1 | 88 |
| YPL270W | 2.1 | 0.6 | 57 |
| YDR498C | 34.9 | 2.9 | 39 |
| YJR043C | 2 | 0.8 | 76 |
| YBR179C | 1.9 | 0.2 | 24 |
| YEL062W | 5.2 | 0.8 | 14 |
| YNL190W | 4.2 | 1.4 | 17 |
| YPR190C | 11.4 | 1.4 | 27 |
| YBR056W | 7.3 | 0.8 | 3 |
| YDR533C | 4.9 | 0.6 | -84 |
| YDL029W | 4.9 | 0.6 | -1039 |
| YBR242W | 67.8 | 7.3 | 33 |
| YER152C | 5.6 | 0.5 | -320 |
| YGR193C | 8 | 0.8 | 46 |
| YER159C | 17.1 | 5 | -1047 |
| YNL167C | 0.6 | 0.2 | 18 |
| YAL053W | 1.7 | 0.2 | 29 |
| YPR144C | 9.1 | 0.5 | 39 |
| YBR256C | 12.6 | 0.9 | 280 |
| YOR099W | 17.7 | 2.9 | 3 |
| YJL145W | 9.7 | 1 | 331 |
| YML111W | 1.4 | 0.3 | 2 |
| YHR205W | 0.4 | 0.1 | 32 |
| YGR281W | 1.7 | 0.2 | 65 |
| YDR481C | 2.3 | 0.3 | 3 |
| YPR148C | 5.7 | 0.7 | 38 |
| YBR028C | 3.1 | 0.5 | 64 |
| YPR187W | 1.5 | 0.6 | -3704 |
| YOR084W | 7.6 | 0.8 | 63 |
| YDL146W | 6.8 | 0.6 | 3 |
| YML098W | 17 | 3.1 | 32 |
| YBL032W | 2.7 | 0.7 | -283 |
| YMR114C | 8.6 | 2.5 | 49 |
| YBR084C-A | 6 | 1 | 985 |
| YDR122W | 0.8 | 0.2 | 487 |
| YDL137W | 55.8 | 4.5 | 113 |
| YDR097C | 1.4 | 0.3 | 31 |
| YLR390W | 74.5 | 10.9 | 190 |
| YDR399W | 30.2 | 4.3 | -74 |
| YNL180C | 1 | 0.3 | 29 |
| YHR104W | 10.1 | 0.9 | 152 |
| YDR316W | 15.9 | 1.8 | 24 |
| YDR176W | 0.9 | 0.2 | 28 |
| YJR063W | 60.3 | 8.2 | 90 |
| YLL036C | 9.9 | 0.8 | 253 |
| YNL247W | 2.7 | 0.3 | 39 |
| YLR251W | 102.7 | 4.1 | 44 |
| YOR188W | 0.6 | 0.2 | 41 |
| YAL019W | 1.6 | 0.3 | 28 |
| YOR108W | 4.7 | 0.4 | 38 |
| YMR064W | 17.5 | 1.3 | 84 |
| YOL049W | 7 | 0.8 | 75 |
| YOR224C | 43.9 | 5.8 | -5096 |
| YBR148W | 2.2 | 0.4 | 18 |
| YKL125W | 8.6 | 1.7 | 31 |
| YGR257C | 8.2 | 0.7 | 32 |
| YMR079W | 72.7 | 7.3 | 149 |
| YNL134C | 4.2 | 0.3 | 104 |
| YFR047C | 9.9 | 0.5 | 239 |
| YER019W | 2.6 | 0.3 | 58 |
| YML091C | 2 | 0.1 | 2 |
| YMR073C | 11.5 | 0.9 | 114 |
| YIR022W | 30.1 | 2.5 | 22 |
| YBR265W | 116.8 | 5.6 | 206 |
| YGR056W | 1.5 | 0.2 | 32 |
| YER044C | 385.9 | 12.7 | 3 |
| YLR006C | 1.6 | 0.3 | 11 |
| YDR173C | 24.1 | 4.4 | 367 |
| YPR162C | 7 | 0.5 | 67 |
| YCR077C | 1.4 | 0.2 | 15 |
| YDL201W | 10.1 | 0.9 | 9287 |
| YDL088C | 0.7 | 0.2 | 32 |
| YDR194C | 3.4 | 0.8 | 162 |
| YFR015C | 2 | 0.3 | 36 |
| YOL144W | 3.7 | 0.3 | 268 |
| YNL039W | 1.6 | 0.4 | 13 |
| YJL149W | 3.4 | 0.5 | 3 |
| YOL113W | 3.1 | 0.4 | 21 |
| YKR088C | 3 | 0.5 | 98 |
| YOR308C | 1.6 | 0.4 | 22 |
| YDR119W | 3 | 0.6 | 9 |
| YGR203W | 961.5 | 126.5 | 70 |
| YHR132W-A | 27.5 | 7.1 | 734 |
| YNL131W | 31.3 | 5.6 | -197 |
| YLR285W | 10 | 0.5 | -229 |
| YFR052W | 11.2 | 0.7 | -171 |
| YLR001C | 2.6 | 0.2 | 36 |
| YOR367W | 97.6 | 8.2 | 1300 |
| YBR267W | 11.7 | 2.5 | 116 |
| YFR009W | 3.2 | 0.2 | 47 |
| YBR115C | 1.1 | 0.1 | 117 |
| YKL194C | 13.1 | 0.7 | 134 |
| YOL009C | 10.5 | 2.4 | 24 |
| YDL090C | 15.5 | 0.9 | 65 |
| YKR038C | 4.1 | 0.3 | 279 |
| YPR023C | 1 | 0.4 | 70 |
| YDL147W | 28.9 | 2.1 | 87 |
| YDR124W | 18.6 | 0.9 | 65 |
| YHR108W | 1.5 | 0.4 | 58 |
| YOL146W | 28.1 | 2.6 | 19 |
| YDR283C | 1.4 | 0.2 | 29 |
| YGR277C | 42.5 | 4.4 | -587 |
| YLR077W | 2.4 | 0.3 | 43 |
| YOR329C | 0.4 | 0.1 | 13 |
| YKR002W | 3.4 | 0.5 | 23 |
| YDR457W | 0.7 | 0.1 | 46 |
| YPL210C | 8.8 | 0.7 | 53 |
| YMR303C | 3.5 | 0.3 | 41 |
| YLR418C | 1.4 | 0.3 | 130 |
| YHL041W | 185.3 | 10.9 | -255 |
| YPR102C | 44.4 | 7.8 | 71 |
| YBR169C | 7.1 | 0.6 | 5252 |
| YOL034W | 3 | 0.3 | 453 |
| YIL069C | 22.6 | 7.2 | -392 |
| YER029C | 2.4 | 0.9 | -179 |
| YMR234W | 4.5 | 0.4 | 25 |
| YIL108W | 1.8 | 0.2 | 19 |
| YPL055C | 0.4 | 0.2 | 15 |
| YPR106W | 4.8 | 0.5 | 14 |
| YDR178W | 42.7 | 4.9 | 481 |
| YJL133W | 10.5 | 3.4 | 37 |
| YBR143C | 5.8 | 0.4 | 62 |
| YDR272W | 19.6 | 3.1 | 298 |
| YNR026C | 3.1 | 0.4 | 61 |
| YGL194C | 6.9 | 0.7 | 125 |
| YLR212C | 4.4 | 0.6 | 41 |
| YOR046C | 10 | 0.8 | 70 |
| YJR007W | 87.6 | 11.7 | 42 |
| YOL068C | 5.1 | 0.7 | 56 |
| YKR045C | 5.9 | 2.4 | 39 |
| YDR262W | 4 | 0.3 | 83 |
| YPL229W | 9.9 | 2.5 | 84 |
| YHR136C | 87.6 | 9 | 39 |
| YPL117C | 10.4 | 2.2 | 136 |
| YPR152C | 6.1 | 0.8 | 1214 |
| YCR065W | 1.7 | 0.4 | 15 |
| YJR145C | 16.8 | 1 | 53 |
| YMR093W | 8.9 | 1.5 | 122 |
| YHR115C | 1.8 | 0.3 | 24 |
| YDR017C | 0.9 | 0.2 | 19 |
| YBL019W | 2.7 | 0.3 | 40 |
| YJL035C | 10 | 0.8 | 14 |
| YOR250C | 31.5 | 2.2 | 9 |
| YJL187C | 1.3 | 0.2 | 88 |
| YMR196W | 1.3 | 0.1 | 346 |
| YLR350W | 11 | 0.9 | 76 |
| YBR260C | 3.8 | 0.8 | 45 |
| YGR243W | 15.3 | 1 | 44 |
| YJL167W | 82.3 | 4 | 13 |
| YER163C | 7.5 | 1 | 67 |
| YOR118W | 12.9 | 1.3 | 86 |
| YER063W | 1.8 | 0.8 | 63 |
| YNL094W | 5.5 | 1.4 | 64 |
| YBR188C | 19.6 | 4 | -268 |
| YOR090C | 2.2 | 0.4 | 27 |
| YDR463W | 1.6 | 0.3 | 23 |
| YKL154W | 14.5 | 1 | 51 |
| YFL045C | 6.1 | 0.6 | 39 |
| YNL216W | 0.9 | 0.2 | 42 |
| YGL028C | 3.1 | 0.4 | -242 |
| YOL021C | 2.2 | 0.2 | 27 |
| YHR047C | 3.1 | 0.3 | 28 |
| YIL133C | 20.2 | 1.1 | 1308 |
| YPL067C | 42.9 | 5.4 | 45 |
| YPL026C | 3.5 | 0.4 | 879 |
| YLR254C | 28.9 | 3.9 | 45 |
| YDR228C | 2.8 | 0.3 | 34 |
| YDL019C | 0.9 | 0.2 | 15 |
| YPR116W | 12.7 | 0.9 | 960 |
| YMR262W | 11.9 | 0.9 | 39 |
| YCR090C | 101.4 | 10.7 | 8 |
| YGL211W | 42.9 | 2.8 | -292 |
| YML012W | 83 | 6.3 | 215 |
| YNL093W | 21.5 | 4.1 | 28 |
| YGR152C | 15.9 | 4.6 | 11 |
| YGR262C | 20.3 | 1 | -142 |
| YPL104W | 2.5 | 0.2 | 65 |
| YDL012C | 35.7 | 11 | 320 |
| YLR436C | 0.6 | 0.1 | 40 |
| YBR066C | 5.2 | 0.9 | 10 |
| YMR286W | 1390.2 | 250 | -131 |
| YPR016C | 35.1 | 3.3 | 69 |
| YBR025C | 40.1 | 2.9 | 136 |
| YFL044C | 9.9 | 0.8 | 69 |
| YDR409W | 0.6 | 0.2 | 7 |
| YDR004W | 4.2 | 0.4 | 34 |
| YAL005C | 3.3 | 0.4 | 77 |
| YDR248C | 116.8 | 14.5 | 44 |
| YMR199W | 1.5 | 0.2 | 6 |
| YDL098C | 36.8 | 4.3 | 35 |
| YGR038W | 14.4 | 1 | 151 |
| YOR001W | 1.3 | 0.4 | 19 |
| YOR112W | 2.8 | 0.6 | 39 |
| YMR005W | 1.9 | 0.6 | 23 |
| YDR382W | 309.1 | 18.1 | -7587 |
| YLR011W | 64.1 | 4.5 | 84 |
| YML053C | 3 | 0.8 | 12 |
| YOR330C | 4.3 | 0.4 | 17 |
| YGR085C | 44.2 | 7.8 | -77 |
| YPL112C | 34 | 3.2 | 180 |
| YMR201C | 2 | 0.5 | 31 |
| YJR105W | 6.7 | 0.9 | 60 |
| YMR304W | 2.9 | 0.3 | 20 |
| YMR086W | 0.3 | 0.1 | 40 |
| YKL217W | 16 | 1 | 29 |
| YDR385W | 5.1 | 0.5 | -626 |
| YHR120W | 3.5 | 0.4 | 78 |
| YBL093C | 3.1 | 0.9 | 123 |
| YPL209C | 4.6 | 0.7 | 78 |
| YOR018W | 1.7 | 0.3 | 16 |
| YPL274W | 20.4 | 1.9 | 16 |
| YDL067C | 1916.8 | 58.9 | -957 |
| YML114C | 5.4 | 1.2 | 24 |
| YNL322C | 76.5 | 8.5 | 14 |
| YMR019W | 1.2 | 0.2 | 3 |
| YML013W | 2.7 | 0.4 | 51 |
| YBR101C | 6.6 | 0.5 | 154 |
| YJL134W | 17 | 3.1 | 12 |
| YDL235C | 8.3 | 1 | 107 |
| YFR033C | 1.2 | 1.1 | 146 |
| YOR299W | 1.8 | 0.2 | 62 |
| YER171W | 5.6 | 0.5 | 31 |
| YDR063W | 41.2 | 9.4 | -511 |
| YNL022C | 12.4 | 1 | 49 |
| YCR059C | 55.3 | 5.9 | 42 |
| YBR059C | 0.5 | 0.1 | 131 |
| YDL018C | 30.1 | 3.2 | -102 |
| YBL052C | 1.7 | 0.2 | 7 |
| YKL103C | 2.9 | 0.3 | 119 |
| YPR168W | 1.2 | 1 | 55 |
| YPR120C | 4.3 | 0.7 | 44 |
| YJR136C | 47 | 2.5 | 151 |
| YAL008W | 27.6 | 4.1 | 26 |
| YMR251W-A | 11.5 | 1 | 22 |
| YPL063W | 3.3 | 0.5 | 39 |
| YLL027W | 5.1 | 0.9 | 85 |
| YML020W | 2.6 | 0.3 | 19 |
| YML126C | 25.5 | 2 | 46 |
| YIL033C | 12 | 1.9 | 65 |
| YIL126W | 0.8 | 0.1 | -166 |
| YOL088C | 4.8 | 0.5 | 35 |
| YDL241W | 41.3 | 5.5 | 13 |
| YKL189W | 22.8 | 2.3 | 19 |
| YDR448W | 3.2 | 0.4 | 59 |
| YGR003W | 10.7 | 0.7 | 19 |
| YDL233W | 1.2 | 0.4 | 45 |
| YIL041W | 8.8 | 0.8 | 87 |
| YPR083W | 0.6 | 0.2 | 33 |
| YDR397C | 39.2 | 5.7 | 99 |
| YGR247W | 13.5 | 1 | 21 |
| YMR284W | 13.2 | 1.7 | 20 |
| YJL196C | 41.6 | 6.1 | 17 |
| YLR268W | 63.6 | 5.8 | 17 |
| YOL045W | 2.3 | 0.3 | 45 |
| YNL273W | 2 | 0.2 | 103 |
| YMR258C | 4.4 | 0.3 | 53 |
| YJL107C | 1.4 | 0.4 | 100 |
| YDR072C | 3.3 | 0.7 | 23 |
| YLR105C | 3.8 | 0.5 | 28 |
| YOR071C | 26.4 | 1.7 | 11 |
| YPL100W | 0.8 | 0.2 | 56 |
| YGL128C | 30.5 | 2.5 | 46 |
| YOR238W | 10.3 | 0.9 | 23 |
| YNR061C | 12.6 | 1.1 | 7 |
| YNL015W | 76.3 | 11.4 | 106 |
| YFR013W | 2.9 | 0.4 | 87 |
| YIL051C | 32.4 | 3.6 | 809 |
| YJR015W | 9.1 | 0.6 | 13 |
| YDR476C | 132.6 | 8.7 | 50 |
| YAL055W | 4.1 | 1.1 | 70 |
| YMR253C | 12.8 | 2.2 | 17 |
| YGL008C | 2.6 | 0.3 | 45 |
| YJL165C | 0.3 | 0.1 | -196 |
| YJL171C | 2.1 | 0.2 | 22 |
| YDL149W | 0.4 | 0.1 | 2 |
| YMR301C | 4.1 | 0.3 | 34 |
| YGR149W | 9.8 | 0.7 | 38 |
| YEL047C | 1.8 | 0.3 | 201 |
| YOR279C | 3.4 | 0.7 | 21 |
| YEL043W | 0.6 | 0.1 | 23 |
| YDR070C | 54.5 | 13.2 | 43 |
| YGL048C | 9.2 | 1 | 36 |
| YHR137W | 10.9 | 0.7 | 56 |
| YLR091W | 7.4 | 0.7 | 171 |
| YDL176W | 1.1 | 0.2 | 3 |
| YGR261C | 3.7 | 0.5 | 54 |
| YBL036C | 13 | 0.9 | -80 |
| YBL007C | 0.7 | 0.2 | 41 |
| YEL032W | 2.5 | 0.4 | 31 |
| YOR362C | 1.6 | 0.3 | 53 |
| YDR132C | 5 | 0.5 | 11 |
| YBR156C | 1.8 | 0.5 | 13 |
| YDR505C | 0.3 | 0.1 | 21 |
| YFL017W-A | 5.5 | 0.5 | 297 |
| YGL068W | 71 | 11.2 | 59 |
| YLL032C | 0.8 | 0.2 | 27 |
| YNL207W | 1 | 0.3 | 61 |
| YJL069C | 0.8 | 0.2 | 60 |
| YCL055W | 4.6 | 0.7 | 17 |
| YDL117W | 0.9 | 0.2 | 41 |
| YHR063C | 2.2 | 0.2 | 205 |
| YDR492W | 82.3 | 5.8 | 49 |
| YGL241W | 3.8 | 0.3 | 32 |
| YFR048W | 3.7 | 1 | 2 |
| YGL231C | 8.3 | 0.9 | 17 |
| YLL043W | 0.6 | 0.3 | 30 |
| YOR267C | 1.6 | 0.3 | 24 |
| YDR325W | 1.4 | 0.2 | 23 |
| YOL115W | 1.8 | 0.4 | 43 |
| YMR164C | 0.4 | 0.1 | 19 |
| YHR035W | 6.4 | 0.6 | 38 |
| YMR315W | 3.2 | 0.4 | 53 |
| YBL057C | 30 | 5.1 | 34 |
| YPR159W | 2.4 | 0.3 | 324 |
| YOL020W | 7.9 | 0.7 | 26 |
| YNR029C | 3.2 | 0.3 | 40 |
| YKL182W | 1.1 | 0.1 | -66 |
| YOR209C | 3.2 | 0.3 | 176 |
| YDR267C | 5.4 | 0.4 | 97 |
| YPR153W | 27.2 | 1 | 19 |
| YBR207W | 2.6 | 0.3 | 60 |
| YJL076W | 0.3 | 0.1 | 43 |
| YER172C | 1.6 | 0.2 | 8 |
| YGR173W | 27.5 | 2.9 | 150 |
| YMR289W | 4.1 | 0.3 | -456 |
| YBR026C | 7 | 0.5 | 23 |
| YGR034W | 9.2 | 1.2 | -120 |
| YPL213W | 3.5 | 0.5 | 175 |
| YDR061W | 1.8 | 0.2 | 37 |
| YMR139W | 15.1 | 3 | 21 |
| YGR078C | 18.5 | 3 | 38 |
| YBR246W | 4.9 | 0.5 | 83 |
| YMR208W | 6.7 | 0.7 | 43 |
| YPL203W | 8.3 | 0.6 | 25 |
| YGR091W | 5.5 | 0.8 | 33 |
| YKL001C | 193.3 | 17.4 | 5488 |
| YDL121C | 6.7 | 1.1 | -289 |
| YKL124W | 2.3 | 0.4 | 12 |
| YKL038W | 0.3 | 0.1 | 14 |
| YNR032W | 10.4 | 1.7 | 39 |
| YBR194W | 4.7 | 1.1 | 152 |
| YNL278W | 0.6 | 0.1 | 2 |
| YDR372C | 5.2 | 0.9 | 127 |
| YDL078C | 6.5 | 0.5 | 64 |
| YLR426W | 6.8 | 0.5 | 51 |
| YKL019W | 52 | 3.2 | 26 |
| YIL075C | 5.2 | 0.4 | 400 |
| YGR008C | 3.4 | 1 | 75 |
| YDL240W | 4 | 0.5 | 62 |
| YNL232W | 2.6 | 0.4 | 53 |
| YKL063C | 14.1 | 3.7 | 12 |
| YGL064C | 4.9 | 0.6 | 121 |
| YIL172C | 3.4 | 0.4 | 35 |
| YOL093W | 11 | 1.9 | -118 |
| YLL045C | 37.1 | 3.7 | 80 |
| YFR051C | 2 | 0.5 | 49 |
| YFL013C | 2.6 | 0.4 | 24 |
| YKL164C | 3.5 | 1 | 2 |
| YGR279C | 3.2 | 0.5 | 23 |
| YDL116W | 11.5 | 0.6 | 230 |
| YPR178W | 2.8 | 0.5 | 57 |
| YMR319C | 6.5 | 0.8 | 116 |
| YNL023C | 2 | 0.2 | 89 |
| YEL017W | 2.8 | 0.7 | 77 |
| YJL155C | 14.9 | 1.8 | 41 |
| YLR179C | 55.1 | 5 | 66 |
| YKL055C | 132.6 | 8.4 | 49 |
| YDR217C | 1.3 | 0.3 | -562 |
| YHR040W | 18.6 | 3.3 | 2 |
| YEL001C | 5.3 | 0.5 | 237 |
| YDL204W | 5 | 1.6 | 193 |
| YNL004W | 2.1 | 0.4 | 3 |
| YJR127C | 0.7 | 0.1 | 61 |
| YOR023C | 2 | 0.5 | 24 |
| YJL072C | 24.6 | 3.9 | -115 |
| YGR278W | 4.7 | 0.6 | 63 |
| YLR121C | 1.4 | 0.3 | 7 |
| YGL220W | 190.1 | 20.9 | 45 |
| YPL049C | 3.8 | 1.2 | 26 |
| YJR047C | 9.5 | 1.1 | 12 |
| YDL047W | 10.3 | 0.8 | 34 |
| YOR157C | 8.6 | 1 | 254 |
| YLR414C | 70.9 | 3.8 | -1810 |
| YNR021W | 9.2 | 0.7 | 24 |
| YHR074W | 4.3 | 0.3 | 94 |
| YNL217W | 32.7 | 2.3 | 41 |
| YKR014C | 3.7 | 0.9 | 773 |
| YLR401C | 1.4 | 0.3 | 19 |
| YNL081C | 32.7 | 11.5 | 15 |
| YGR019W | 10.6 | 0.8 | 67 |
| YOR008C | 1.6 | 0.4 | 25 |
| YKR001C | 7.1 | 0.6 | 79 |
| YNR038W | 4.2 | 0.5 | 74 |
| YBL085W | 1.1 | 0.3 | 3 |
| YBR196C | 3.9 | 0.4 | 540 |
| YMR237W | 1.9 | 0.4 | 46 |
| YGL151W | 1.1 | 0.1 | -496 |
| YNL104C | 4.2 | 0.4 | 42 |
| YGR264C | 3 | 0.3 | 65 |
| YML112W | 26.8 | 3.2 | 94 |
| YLR234W | 3.3 | 0.3 | 27 |
| YOL066C | 7.2 | 1.3 | 42 |
| YOL047C | 35.7 | 2.9 | 35 |
| YNL313C | 12.2 | 0.7 | 47 |
| YGL062W | 1.7 | 0.2 | -345 |
| YLR270W | 2.2 | 0.3 | 38 |
| YJL034W | 2.9 | 0.3 | 124 |
| YDL174C | 6.3 | 0.6 | 61 |
| YLR191W | 1.7 | 0.3 | 44 |
| YPL153C | 1.1 | 0.2 | 2 |
| YOL038W | 9.9 | 4 | -115 |
| YPR137W | 3.6 | 0.3 | 79 |
| YDR490C | 1.5 | 0.3 | 13 |
| YBL011W | 5.2 | 0.7 | 205 |
| YOR293W | 177.7 | 19.5 | -123 |
| YOR295W | 44.4 | 5.4 | 826 |
| YPR122W | 1.3 | 0.2 | 53 |
| YGL207W | 1.3 | 0.2 | 26 |
| YOR266W | 4.7 | 0.4 | 34 |
| YNL009W | 12.6 | 0.9 | 13 |
| YLR146C | 5.1 | 0.5 | 82 |
| YKL149C | 6.9 | 0.7 | 20 |
| YMR134W | 12 | 0.9 | 141 |
| YBR094W | 1.2 | 0.2 | 2 |
| YML006C | 0.8 | 0.2 | 50 |
| YER101C | 4.5 | 0.4 | 19 |
| YGR283C | 37.3 | 3.4 | 40 |
| YEL003W | 98.7 | 15.5 | -308 |
| YDL028C | 1.6 | 0.4 | 3 |
| YGL040C | 9.2 | 0.9 | -96 |
| YOR197W | 1.3 | 0.3 | 19 |
| YBL033C | 2.6 | 0.5 | -497 |
| YJR001W | 2.5 | 0.7 | 25 |
| YDL177C | 11.2 | 3.8 | 110 |
| YOR110W | 3.3 | 0.5 | 32 |
| YNL316C | 6.5 | 0.5 | 53 |
| YKL006W | 712.6 | 55.8 | -266 |
| YNL096C | 597.5 | 29.5 | -170 |
| YDR460W | 4.2 | 0.5 | 82 |
| YNL073W | 2.3 | 0.2 | 50 |
| YML027W | 5.8 | 1.8 | 3 |
| YDR427W | 3.2 | 0.7 | 116 |
| YMR076C | 5.6 | 0.4 | 35 |
| YKR092C | 30.3 | 7.3 | 206 |
| YBL016W | 23.8 | 3.6 | 43 |
| YPL052W | 11.4 | 0.7 | 73 |
| YDR371W | 3.3 | 0.4 | 62 |
| YGR269W | 5.2 | 1.1 | 39 |
| YDR320C | 2.3 | 0.3 | 22 |
| YFR018C | 5.8 | 0.5 | 642 |
| YOR201C | 8.5 | 0.8 | 25 |
| YNL088W | 1.2 | 0.1 | 81 |
| YML019W | 13.5 | 0.9 | 24 |
| YJL083W | 1.3 | 0.3 | 23 |
| YER006W | 2.5 | 0.5 | 738 |
| YHR046C | 9.4 | 1 | 57 |
| YCR083W | 373.3 | 20.6 | 175 |
| YAL034W-A | 6.5 | 0.9 | -599 |
| YNL095C | 7.2 | 1.4 | 83 |
| YDL043C | 4.4 | 0.5 | 108 |
| YMR157C | 83.6 | 8.2 | 26 |
| YJR122W | 1.9 | 0.3 | 46 |
| YDR243C | 3.4 | 0.7 | 31 |
| YJR066W | 1.6 | 0.2 | 5 |
| YGR076C | 53.6 | 7.7 | 64 |
| YOR077W | 2.4 | 0.6 | 38 |
| YGR101W | 52.5 | 3.9 | 56 |
| YBL037W | 4 | 0.3 | 47 |
| YLR215C | 11.8 | 1.9 | 93 |
| YPL134C | 7.7 | 0.5 | 181 |
| YML131W | 31.8 | 4.2 | -212 |
| YMR223W | 3.2 | 0.3 | 11 |
| YIL066C | 2.7 | 0.3 | 64 |
| YOR205C | 4 | 0.7 | 53 |
| YIL063C | 1.2 | 0.5 | 22 |
| YHR192W | 37.1 | 2.6 | 1175 |
| YLR362W | 2.9 | 0.4 | 79 |
| YBR141C | 8.5 | 0.7 | 13 |
| YBL059C-A | 14.4 | 4.3 | 58 |
| YJR003C | 14.1 | 0.8 | 59 |
| YPR043W |  |  | 264 |
| YIL020C | 10.7 | 1 | -344 |
| YDR067C | 14.2 | 0.9 | 126 |
| YLR055C | 0.9 | 0.2 | -541 |
| YOR244W | 22.4 | 2.9 | 208 |
| YNL218W | 3.2 | 0.3 | 22 |
| YJR135C | 40.1 | 2.7 | -150 |
| YOL151W | 7.4 | 0.5 | 90 |
| YDL022W | 7.5 | 0.5 | 250 |
| YNL124W | 0.7 | 0.3 | 82 |
| YGL026C | 2.1 | 0.2 | 29 |
| YPR135W | 3.1 | 0.3 | 49 |
| YNL243W | 6.2 | 0.5 | 480 |
| YDR143C | 0.3 | 0.1 | 7 |
| YKR004C | 30.6 | 1.8 | 53 |
| YGL018C | 17.5 | 5.3 | 46 |
| YKL026C | 4.6 | 0.4 | 50 |
| YOR206W | 3 | 0.4 | 224 |
| YDR357C | 9.9 | 0.9 | 22 |
| YOL094C | 31.3 | 2.8 | -79 |
| YGL185C | 42.2 | 3 | 52 |
| YOR037W | 10.5 | 0.8 | 147 |
| YJR131W | 17.7 | 1.7 | 84 |
| YGL056C | 2 | 0.4 | 63 |
| YGR252W | 8.3 | 0.7 | 44 |
| YFL048C | 1.9 | 0.3 | 13 |
| YOL001W | 16.7 | 2.2 | 208 |
| YOR252W | 11.2 | 1 | 62 |
| YDR226W | 7.5 | 1.1 | 217 |
| YIL136W | 3.8 | 0.8 | 115 |
| YHR216W | 6.9 | 0.5 | 54 |
| YBR139W | 4.6 | 0.4 | 18 |
| YFR046C | 8.9 | 2.3 | 59 |
| YFR004W | 24.7 | 2.9 | 138 |
| YDR245W | 9.1 | 1.6 | 84 |
| YOR156C | 0.9 | 0.2 | 19 |
| YGR027C |  |  | 82 |
| YBL101C | 0.5 | 0.1 | 68 |
| YER079W | 2 | 0.7 | 42 |
| YPR037C | 126.5 | 12.7 | 33 |
| YCR046C | 204.2 | 18.3 | 234 |
| YPL082C | 1.1 | 0.2 | 60 |
| YDR251W | 0.4 | 0.1 | 112 |
| YGL162W | 23.8 | 3.6 | 21 |
| YHL029C | 1.3 | 0.3 | 26 |
| YGL227W | 0.8 | 0.2 | 62 |
| YDR486C | 7.9 | 1.6 | 191 |
| YLR347C | 7.6 | 0.6 | 36 |
| YCL043C | 4.3 | 0.4 | 8 |
| YDL020C | 1.6 | 0.4 | 3 |
| YKL069W | 71 | 5.2 | 25 |
| YOR063W | 3.4 | 0.5 | 120 |
| YOL004W | 0.5 | 0.1 | 17 |
| YDL065C | 1 | 0.3 | 56 |
| YMR143W | 15.1 | 1.2 | 131 |
| YPL019C | 3 | 0.5 | 17 |
| YJL190C | 14.9 | 1 | 78 |
| YHR121W | 40.7 | 4.4 | 38 |
| YJL085W | 3.6 | 0.4 | 12 |
| YJL065C | 14.6 | 4.9 | 29 |
| YJL186W | 4 | 0.3 | 13 |
| YHR156C | 5.5 | 1.2 | 7 |
| YNL297C | 2.3 | 0.1 | 22 |
| YMR078C | 3.7 | 0.3 | 35 |
| YNR034W-A | 157.2 | 10.8 | -78 |
| YOR231W | 1.6 | 0.2 | 18 |
| YNL245C | 54.8 | 8 | 8 |
| YJL143W | 72.6 | 4.7 | 91 |
| YPL066W | 2 | 0.5 | 20 |
| YNR018W | 36.6 | 3.8 | -70 |
| YLR005W | 4 | 0.4 | 16 |
| YNL260C | 31.4 | 3.6 | -116 |
| YCR034W | 7.2 | 0.6 | -73 |
| YER071C | 29 | 3.6 | 67 |
| YNL250W | 2.9 | 0.3 | -924 |
| YPL273W | 28.7 | 1.9 | 129 |
| YPR125W | 20.2 | 2.9 | 44 |
| YOR109W | 1.7 | 0.2 | 68 |
| YPR056W | 9.2 | 0.8 | 13 |
| YMR090W | 9.9 | 0.9 | 109 |
| YLR130C | 4.9 | 0.4 | 55 |
| YOR092W | 5 | 1.3 | 3 |
| YIL057C | 56.3 | 7.6 | 16 |
| YAL021C | 0.8 | 0.2 | 224 |
| YGL005C | 25.8 | 3.5 | -320 |
| YPL233W | 57.1 | 5.3 | 26 |
| YOL116W | 1.2 | 0.4 | 9 |
| YGL219C | 5.6 | 0.7 | 45 |
| YNR058W | 2.7 | 0.3 | 56 |
| YIL151C | 1.6 | 0.2 | 13 |
| YDR156W | 28.9 | 6.1 | -72 |
| YJR057W | 15.6 | 1 | -18395 |
| YMR061W | 1.9 | 0.3 | 7 |
| YJR094W-A |  |  | 64 |
| YGL030W | 255.4 | 12.7 | 146 |
| YER007W | 3.4 | 0.3 | 22 |
| YPL030W | 2.1 | 0.3 | 16 |
| YLL001W | 1 | 0.3 | 31 |
| YCL009C | 26.2 | 2.9 | 105 |
| YIL096C | 3.1 | 0.4 | 25 |
| YPR191W | 56.1 | 3.6 | 352 |
| YDL150W | 0.8 | 0.4 | 31 |
| YIR037W | 10.5 | 1.1 | 44 |
| YGL147C | 9.6 | 1 | 53 |
| YIR026C | 11.6 | 2.7 | 40 |
| YDL084W | 18.2 | 2.8 | 17 |
| YOL051W | 0.3 | 0.1 | 3 |
| YJL101C | 3.5 | 0.4 | 2 |
| YOR230W | 1.8 | 0.3 | 62 |
| YDR532C | 17.1 | 1.8 | 145 |
| YPL226W | 1.2 | 0.2 | 33 |
| YBR138C | 2.4 | 0.4 | 5 |
| YER088C | 1.5 | 0.4 | 18 |
| YBL056W | 1.7 | 0.5 | 54 |
| YMR022W | 11.6 | 1.1 | 31 |
| YDR407C | 3.7 | 0.4 | -978 |
| YIL038C | 1.3 | 0.4 | 64 |
| YNL231C | 32.9 | 3.6 | 69 |
| YMR255W | 4.9 | 2.1 | 66 |
| YBR211C | 6.8 | 0.7 | 68 |
| YGR102C | 50.8 | 6.1 | 65 |
| YDR322W | 29.9 | 2.7 | 1206 |
| YKL135C | 3.8 | 0.6 | 71 |
| YER173W | 1.8 | 0.3 | 15 |
| YBR290W | 6.9 | 1.8 | 48 |
| YDR497C | 10.5 | 0.8 | 59 |
| YJR022W | 18.8 | 1 | -685 |
| YNL280C | 5 | 0.3 | 218 |
| YNR047W | 0.6 | 0.2 | 295 |
| YKL009W | 46.5 | 7.4 | 73 |
| YNL064C | 5.2 | 0.5 | 34 |
| YMR266W | 1.1 | 0.2 | 42 |
| YGR284C | 33.8 | 2.8 | 46 |
| YLR248W | 4.2 | 0.8 | 72 |
| YOR354C | 12.5 | 0.9 | 80 |
| YFR010W | 4.7 | 0.7 | 83 |
| YOR372C | 0.7 | 0.2 | 6 |
| YER078C | 4.5 | 0.6 | 28 |
| YML128C | 4.1 | 0.4 | 34 |
| YMR032W | 2.4 | 0.4 | 32 |
| YDR062W | 23.6 | 2.2 | 29 |
| YDR527W | 2.1 | 0.5 | 61 |
| YGR055W | 3 | 0.3 | 35 |
| YLR301W | 4 | 0.5 | -944 |
| YDR458C | 3.7 | 0.3 | 28 |
| YML051W | 2.4 | 0.3 | 2856 |
| YDR510W | 43.7 | 18.1 | 7 |
| YHR073W | 1 | 0.3 | 39 |
| YDR405W | 23.4 | 3.7 | 5525 |
| YKL005C | 2.3 | 0.8 | 22 |
| YGL238W | 4 | 0.2 | 141 |

**References**

1. Robinson NE, Robinson AB (2001) Prediction of protein deamidation rates from primary and three-dimensional structure. Proc Natl Acad Sci U S A 98: 4367-4372.

2. Solstad T, Carvalho RN, Andersen OA, Waidelich D, Flatmark T (2003) Deamidation of labile asparagine residues in the autoregulatory sequence of human phenylalanine hydroxylase. Eur J Biochem 270: 929-938.

3. Shi Y, Rhodes NR, Abdolvahabi A, Kohn T, Cook NP, et al. (2013) Deamidation of asparagine to aspartate destabilizes Cu, Zn superoxide dismutase, accelerates fibrillization, and mirrors ALS-linked mutations. J Am Chem Soc 135: 15897-15908.

4. Johnson BA, Shirokawa JM, Hancock WS, Spellman MW, Basa LJ, et al. (1989) Formation of isoaspartate at two distinct sites during in vitro aging of human growth hormone. J Biol Chem 264: 14262-14271.

5. Vanbelle C, Halgand F, Cedervall T, Thulin E, Akerfeldt KS, et al. (2005) Deamidation and disulfide bridge formation in human calbindin D28k with effects on calcium binding. Protein Sci 14: 968-979.

6. di Salvo ML, Delle Fratte S, Maras B, Bossa F, Wright HT, et al. (1999) Deamidation of asparagine residues in a recombinant serine hydroxymethyltransferase. Arch Biochem Biophys 372: 271-279.

7. Mikkat S, Kischstein T, Kreutzer M, Glocker MO (2013) Mass spectrometric peptide analysis of 2DE-separated mouse spinal cord and rat hippocampus proteins suggests an NGxG motif of importance for in vivo deamidation. Electrophoresis 34: 1610-1618.

8. Zomber G, Reuveny S, Garti N, Shafferman A, Elhanany E (2005) Effects of spontaneous deamidation on the cytotoxic activity of the Bacillus anthracis protective antigen. J Biol Chem 280: 39897-39906.

9. Robinson NE, Robinson ML, Schulze SE, Lai BT, Gray HB (2009) Deamidation of alpha-synuclein. Protein Sci 18: 1766-1773.

10. Nellis DF, Michiel DF, Jiang MS, Esposito D, Davis R, et al. (2012) Characterization of recombinant human IL-15 deamidation and its practical elimination through substitution of asparagine 77. Pharm Res 29: 722-738.

11. Fukuda M, Takao T (2012) Quantitative analysis of deamidation and isomerization in beta2-microglobulin by 18O labeling. Anal Chem 84: 10388-10394.

12. Nonaka Y, Aizawa T, Akieda D, Yasui M, Watanabe M, et al. (2008) Spontaneous asparaginyl deamidation of canine milk lysozyme under mild conditions. Proteins 72: 313-322.

13. Deverman BE, Cook BL, Manson SR, Niederhoff RA, Langer EM, et al. (2002) Bcl-xL deamidation is a critical switch in the regulation of the response to DNA damage. Cell 111: 51-62.

14. Belle A, Tanay A, Bitincka L, Shamir R, O'Shea EK (2006) Quantification of protein half-lives in the budding yeast proteome. Proc Natl Acad Sci U S A 103: 13004-13009.
